# The spectrum of bleeding in Wiskott-Aldrich syndrome: a systematic review and meta-analysis of incidence and mortality

**DOI:** 10.3389/fimmu.2026.1854833

**Published:** 2026-07-06

**Authors:** Zhicheng Yang, Ziyun Tang, Lina Zhou, Yunfei An, Xiaodong Zhao, Rongxin Dai

**Affiliations:** 1 Department of Rheumatology & Immunology, Children's Hospital of Chongqing Medical University, National Clinical Research Center for Children and Adolescents' Health and Diseases, Ministry of Education Key Laboratory of Child Development and Disorders, Chongqing Key Laboratory of Child Rare Diseases in Infection and Immunity, Chongqing, China; 2 Basic Medicine Research and Innovation Center for Novel Target and Therapeutic Intervention, Ministry of Education, College of Pharmacy, Chongqing Medical University, Chongqing, China

**Keywords:** hemorrhage, inborn error of immunity (IEI), incidence, mortality, Wiskott-Aldrich syndrome (WAS)

## Abstract

**Background:**

Bleeding is the predominant clinical manifestation and one of the leading causes of death of Wiskott-Aldrich syndrome (WAS). However, significant discrepancies in reported bleeding phenotypes persist.

**Objective:**

This systematic review and meta-analysis aim to provide a comprehensive characterization of the bleeding phenotype and elucidate the resultant mortality burden among patients with WAS.

**Methods:**

Observational studies reporting either the cumulative incidence of bleeding manifestations or the occurrence of fatal hemorrhagic events in patients with WAS were included. The Joanna Briggs Institute critical appraisal tool was used to assess the risk of bias. A generalized linear mixed model with a binomial-normal distribution was employed for the meta-analysis. Subgroup analyses, stratified by clinical stage at data collection, and meta-regression analyses were conducted to explore heterogeneity.

**Results:**

A total of 40 studies involving 1865 patients were identified. The pooled cumulative incidences of overall and site-specific bleeding (excluding gastrointestinal bleeding), increased significantly from disease onset to diagnosis and through to the end of follow-up. At the end of follow-up, the pooled cumulative incidence of multisystem bleeding and severe bleeding was 57% (95% CI 41-72) and 19% (95% CI 12-28), respectively. Both sample size and proportion of patients with a WAS score of 5 were associated with cumulative incidence of severe bleeding at the end of follow-up (*p* = 0.0042 and 0.0423, respectively). The cause-specific mortality rate from hemorrhage was 9.3% (95% CI: 6.1-13.8; *I*^2^ = 0%) in non-curatively treated patients and 1.0% (95% CI: 0.4-2.4; *I*^2^ = 0%) in curatively treated patients. Curative treatment was associated with a significantly lower risk of fatal hemorrhage compared to non-curative treatment (RR = 0.07, 95% CI 0.01-0.49). The proportional mortality ratio due to hemorrhage was 30% (95% CI 22-39) in non-curatively treated patients. Fatal hemorrhages were highly skewed toward early childhood (median age-at-death: 22 months), with intracranial hemorrhage being the predominant cause of death (76.1%).

**Conclusions:**

Bleeding complications are nearly universal and progressive in patients with WAS. Given that most fatal hemorrhages occur in early childhood and curative treatment significantly mitigates this risk, prompt implementation of such therapy is imperative.

**Systematic review registration:**

https://www.crd.york.ac.uk/prospero/, identifier CRD420261277891.

## Introduction

Wiskott-Aldrich syndrome (WAS) is a rare, X-linked primary immunodeficiency disorder characterized by recurrent infections, eczema, microthrombocytopenia, and an increased risk of autoimmune diseases and lymphoid malignancies. The clinical manifestations of WAS span a broad continuum, stratified by a standardized scoring system: the mild phenotype (WAS score 1-2) is characterized primarily by thrombocytopenia and/or eczema; the classic phenotype (WAS score 3-4) is accompanied by recurrent infections; and the severe phenotype (WAS score 5) is defined by the development of autoimmune disease or malignancy (1–3).

Pathogenetically, WAS results from *WAS* gene mutations that disrupt WAS protein (WASP) expression. As WASP is indispensable for actin polymerization, its deficiency leads to profound cytoskeletal derangements that inherently impair megakaryocyte maturation and proplatelet formation, leading to insufficient platelet production. Meanwhile, structurally abnormal platelets also exhibit impaired adhesion and aggregation, and are prone to accelerated peripheral clearance (2, 4). Consequently, microthrombocytopenia, the hallmark of the disease, predisposes patients to a spectrum of hemorrhagic manifestations, which range from minor petechiae to life-threatening visceral hemorrhages (5). Accordingly, standard supportive care for bleeding prophylaxis has been widely adopted in routine clinical practice, including trauma avoidance, local hemostatic management, and transfusion support for severe thrombocytopenia and acute hemorrhage.

Extensive clinical evidence has characterized the hemorrhagic profile and its associated mortality risks within this patient population. Bleeding is the predominant presenting feature of WAS, with historical data indicating that over 80% of patients have a history of bleeding at the time of diagnosis (3, 5). Critically, this hemorrhagic diathesis dictates clinical outcomes, representing the second leading cause of mortality, surpassed only by severe infections (6).

Extant studies demonstrate marked inconsistencies in WAS bleeding phenotype. The reported incidence of site-specific bleeding manifestations varies considerably due to heterogeneity in study design and in disease stage and severity at assessment. Furthermore, evidence regarding fatal hemorrhagic events is often hampered by limited sample size and cohort-specific bias, precluding precise mortality estimates and a systematic profiling of specific characteristics, such as site of bleeding and patient age at the event.

To address these critical knowledge gaps, this study comprehensively characterizes the bleeding phenotype and the resultant mortality burden in patients with WAS. Specifically, we pooled the cumulative incidences of overall, site-specific, severe, and multisystem bleeding across different clinical stages. Furthermore, mortality risk was rigorously evaluated through the quantitative synthesis of the cause-specific mortality rate from hemorrhage (CSMR-H) and the proportional mortality ratio due to hemorrhage (PMR-H) across stratified cohorts receiving either curative or non-curative treatment. Finally, we performed a descriptive analysis of the age and anatomical site distributions of fatal hemorrhagic events in non-curatively treated patients. These findings provide a robust evidence base to enhance clinical awareness and risk stratification for hemorrhagic manifestations in WAS.

## Methods

This systematic review and meta-analysis was conducted in accordance with a pre-registered protocol (PROSPERO ID: *CRD420261277891*) and adhered to the Preferred Reporting Items for Systematic Reviews and Meta-Analyses (PRISMA) and Meta-analysis of Observational Studies in Epidemiology (MOOSE) guidelines (**Supplementary Table 1**) (7, 8).

### Search strategy

We comprehensively searched PubMed, Embase, Scopus, Web of science, and the Cochrane Library (from inception to December 26, 2025) with subject headings and free-text terms for “Wiskott-Aldrich syndrome” and “Hemorrhage”, supplemented by manual backward citation searching (**Supplementary Table 2**). This process was independently conducted by two trained pediatric immunology researchers (Z.Y. and Z.T.).

### Study selection

Retrieved references were imported into EndNote for both automated and manual deduplication; after title/abstract screening by two independent reviewers (Z.Y. and Z.T.), eligible records were transferred to Zotero for full-text retrieval and eligibility assessment.

Studies were eligible if they met both of the following criteria (1): original observational studies involving patients with a genetically or clinically confirmed diagnosis of WAS; and (2) reporting either the cumulative incidence of bleeding manifestations or the occurrence of fatal hemorrhagic events. Conversely, exclusion was based on any of the following: (1) publication date prior to January 1, 1976; (2) interventional studies; (3) overlapping data with other included studies; (4) no data of interest (either did not report the outcomes of interest or provided incomplete reporting of these outcomes) or (5) data of questionable reliability. The year 1976 was selected as the start date because earlier publications are frequently inaccessible in full-text form. Accordingly, this restriction to the subsequent five decades ensures a consistent and verifiable evidence base for analysis. Interventional studies were excluded as their stringent eligibility criteria and protocol-mandated management may introduce methodological bias, potentially yielding estimates that do not represent real-world patient populations. No language restrictions were applied, with professional machine translation tools (DeepL Translator) employed to process non-English literatures. To ensure data independence, we prioritized the inclusion of multicenter studies or those with the largest sample sizes, unless overlapping reports provided unique data for distinct outcomes of interest. Conflicts were first resolved by discussion between the two screeners, and any persisting conflicts were then adjudicated by the senior reviewers (X.Z. and R.D.).

### Data extraction

Two independent reviewers (Z.Y. and Z.T.) extracted data from included studies using a standardized, validated Microsoft Excel form. The predefined data extraction items, covering study characteristics, patient baseline characteristics, and core clinical outcomes are detailed in **Supplementary Table 3**. In this analysis, end of follow-up was defined as the end of available observation recorded in primary studies. The median and range of follow-up duration were also calculated based on this unified endpoint. Severe bleeding was defined on the basis of original study reports: it corresponded to the highest bleeding grade in studies with formal grading systems, or was regarded as hemorrhage requiring urgent medical intervention in studies without predefined grading criteria. Unreported outcomes of interest were recorded as missing data without assumptions. The two reviewers achieved an initial agreement of 80%, with discrepancies resolved by consensus. All extracted data were subsequently verified by all other review authors.

### Quality assessment

The methodological quality of included studies was independently evaluated by three authors (Z.Y., Z.T., and R.D.) applying the Joanna Briggs Institute (JBI) critical appraisal tool specific to each study design (cohort, case-control, case series, and case report) (9). Based on the total percentage of “yes” responses, the overall quality of evidence for each study was categorized as follows: high quality (>80%), moderate quality (60-80%), or low quality (<60%). Any disagreements were resolved through consensus discussion with the wider author group.

### Statistical analysis

All Statistical analyses were conducted in R software (version 4.5.2), using the metaprop function within the meta package. Given the rarity of WAS, our synthesis included a substantial number of small-scale studies (sample sizes ranging from 5 to 10). To address the statistical challenges inherent in sparse data and extreme proportions (approaching 0 or 1), a generalized linear mixed model with a binomial-normal distribution was employed (10). To account for the substantial between-study heterogeneity inherent in meta-analyses of single proportions from observational studies, which was further exacerbated by extreme proportions, we adopted the random-effects model for all pooled analyses to ensure conservative statistical inference (11).

Between-study heterogeneity was assessed using the Cochran’s Q test and quantified by the *I*^2^ statistic, with *I*^2^ ≥ 50% considered indicative of substantial heterogeneity. Where appropriate, heterogeneity was explored through pre-specified subgroup analyses stratified by the clinical stage at data collection (disease onset, diagnosis, and the end of follow-up). Furthermore, the moderating effects of sample size and overall disease severity (the proportion of patients with a WAS score of 5, indicating complications of autoimmunity or malignancy) on the cumulative incidence of severe bleeding at the end of follow-up were assessed using meta-regression analyses.

For meta-analyses containing at least ten studies, potential publication bias was evaluated by visual assessment of funnel plot asymmetry and Egger’s regression test; Peters’ test was supplemented for analyses with extreme proportions as it is more robust against mathematical artifacts that cause false-positive Egger’s test results in this setting (12, 13). The stability of the findings was assessed via leave-one-out sensitivity analyses, where each study was sequentially excluded to determine its influence on the summary estimate. The pooled result was considered robust if no material change in the effect size was observed upon study removal. The strategy to address participant overlap across included studies is presented in **Supplementary Table 4**.

## Results

### Search results

**Figure 1:**
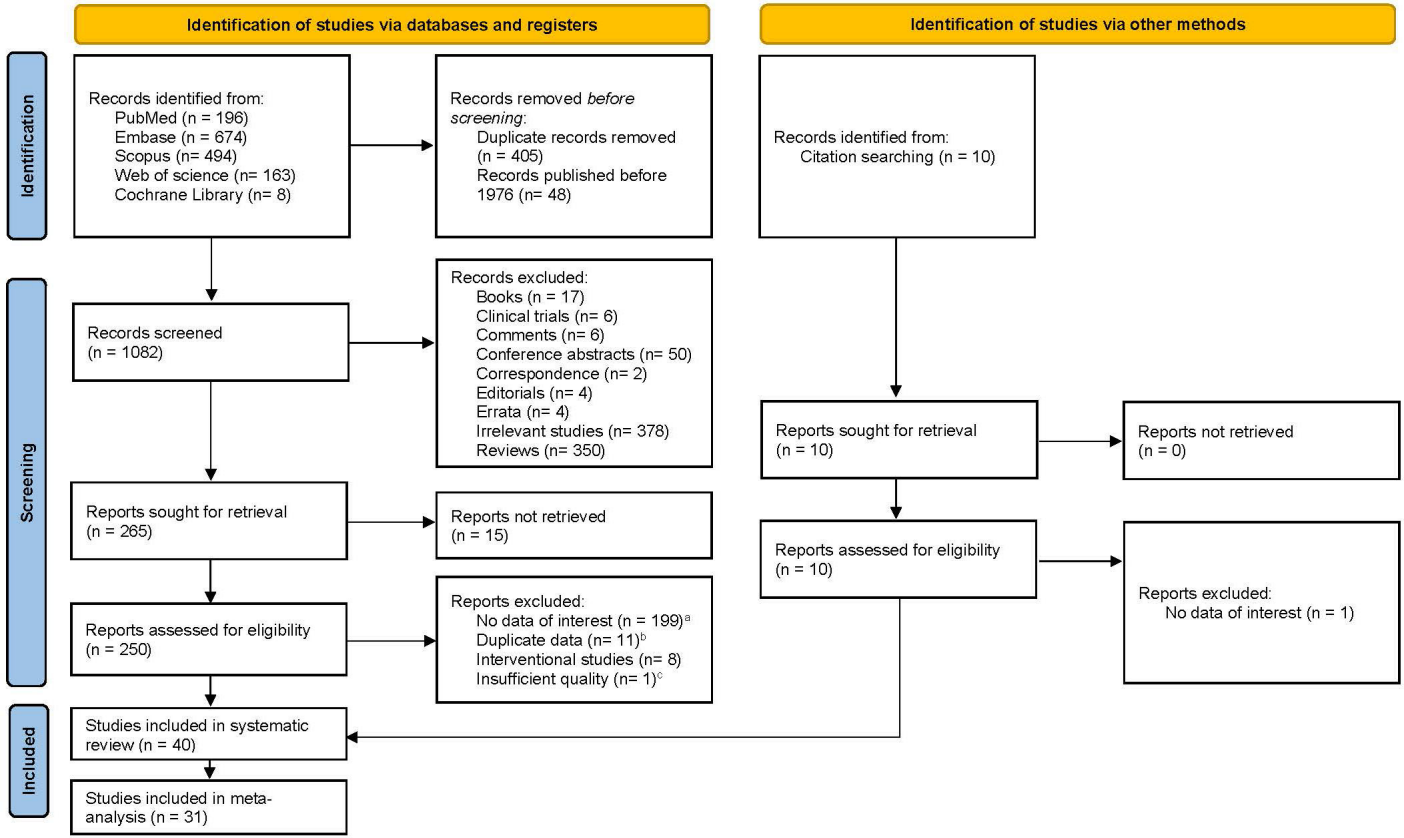
PRISMA flow chart showing the study selection process. (a) Three large-scale HSCT-related observational studies (Moratto 2011, Albert 2022) were excluded due to incomplete reporting of mortality data; Vallée et al. (2024), a global multicenter study, was excluded as it failed to stratify mortality data according to whether patients received definitive treatment. (b) Mallebranche et al. (2025), a large-scale cohort from France was excluded due to potential patient overlap with another global multicenter study (Albert 2010) and incomplete reporting of mortality data. (c) Espejo et al. (2023), a large-scale cohort from Spain was excluded due to questionable data reliability (specifically, a high proportion of non-genetically confirmed female patients).

The initial systematic search yielded 1,535 records. After removing duplicates and records published before 1976, 1,082 titles and abstracts were screened, of which 817 were excluded. Of the 265 articles retrieved for full-text assessment, 15 were unavailable. Following a rigorous full-text review of the remaining 250 studies, 219 were excluded for not meeting the eligibility criteria (see Method). Manual reference tracking of the included studies identified an additional ten records, nine of which were eligible for inclusion. Ultimately, 40 studies were included in this systematic review (3, 5, 14–51), of which 31 provided sufficient data for the quantitative meta-analysis (3, 5, 14–16, 18, 20–35, 37–42, 45, 47, 48), while the remaining nine studies were exclusively included in the qualitative synthesis (**Figure 1**) (17, 19, 36, 43, 44, 46, 49–51).

### Characteristics of included studies

Geographically, the 40 included studies originated from diverse regions: 20 in Asia, seven in the Americas, six in the Middle East, five in Europe, and one in Australia; only one was a global multicenter study, with cohorts primarily from North America and Western Europe (Albert, 2010). Demographically, A marked male predominance was observed; only 12 female patients were identified in six studies (**Table 1**) (15, 16, 27, 28, 41, 52). The participating study institutions of included studies were documented in **Supplementary Table 5**.

**Table 1:** Characteristics of included studies.

Author, publication year	Country	Enrollment period	Study design	Sample size (fm^a^)	Patients receiving CT, n	Classic/mild WAS^b^, n	Age at onset, months (median; mean; range)	Age at diagnosis, years (median; mean; range)	Age at last follow-up, years (median; mean; range)	Follow-up duration, years (median; mean; range)
Somerville et al., 1993 (47)	Australia	1960-1990	Case series	9	1	7/25	2; 21.1; 0-144	—	8; 7.7; 0.25-15	—
Santos et al., 2025 (14)	Brazil	—	Retrospective cohort	22	12	20/2	—	1.3; —; 0.2-22.6	—	—
Chen et al., 2015 (34)	China	2004-2014	Retrospective cohort	53	8	47/6	—; —; 0-16	—; 1; 0.1-12.3	—	—
Huang et al., 2023 (21)	China	2016-2022	Case series	11	6	8/3	—; —; 0-0.9	—	—	—
Jiang et al., 2011 (39)	China	2006-2010	Case series	6	1	6/0	—	2; —; 0.5-5.0	—	—; —;NA-4
Jiang et al., 2022 (23)	China	2018-2020	Case series	5	1	4/1	0.3; 3.7; 0-17	1.8; 3.7; 0.3-9.9	—	—
Jin et al., 2019 (32)	China	2004-2016	Retrospective cohort	42	8	39/3	1; —; 0-10	—	—	—
Lee et al., 2008^c,e^ (43)	China	1980-2006	Retrospective cohort	11	5	11/0	0.8; 1.5; 0.1-5	—	3; —; 0.3-24.5	—
Lee et al., 2009 (42)	China	1991-2008	Retrospective cohort	35	9	35/0	0; 1.3; 0-8	0.9; —; 0.1-3.5	—	2; —; 0.4-7
Lee et al., 2010 (40)	China	1993-2009	Retrospective cohort	16	6	12/4	2; 2.6; 0.1-12	0.4; 3.5; 0-23	4.1; 6.9; 0.9-25.8	—
Li et al., 2015 (33)	China	2000-2015	Retrospective cohort	132	36	112/20	0.5; -; 0-52	0.8; —; 0.1-22.1	—; —; NA-25.6	—
Luo et al., 2021 (26)	China	2007-2020	Case-control	165	90	—	0.1(D), 1(S); —; —	0.5(D), 0.8(S); —; —	1.6(D), 5(S); —; —	—
Luo et al., 2023 (20)	China	2018-2021	Case series	10	10	10/0	—	0.3; 0.3; 0.1-0.5	1.1; —; 0.4-1.9	1.2; 1.3; 0.1-3.3
Wang et al., 2020 (28)	China	2015-2019	Retrospective cohort	23 (1)	9	14/9	1; 1.4; 0-6	0.5; —; 0.2-10.2	—; —; NA-9	—
Zheng et al., 2019 (31)	China	2013-2018	Retrospective cohort	31	24	25/6	1; —; 0-83	0.3; —; 0.1-8	—	1.7; —; 0.4-3.1
Zhou et al., 2023 (18)	China	2006-2020	Retrospective cohort	60	60	35/19^f^	—	0.4; —; —	—	—
Mahlaoui et al., 2013 (35)	France	1983-2010	Retrospective cohort	26	22	26/0	6.2; —; 2-22.5	0.8; —; —	7(S); —; 2-28(S)	—
David et al., 2012 (38)	India	—	Case series	9	2	6/3	2.5; —; 1-12	—	—	—
Suri et al., 2021 (25)	India	—	Retrospective cohort	95	25	81/14	3; —; 0-168	1; —; 0.2-32	7.2; —; 0.8-34.8	3; —; 0-12
Esmaeilzadeh et al., 2025 (16)	Iran	2014-2024	Retrospective cohort	41 (5)	—	—	—	11.6; —; 0.25-37	—	—; —; NA-10
Palevski et al., 2023^c,d^ (19)	Israel	—	Case series	4	2	4/0	—	0.6; 0.6; 0.5-0.9	—	—
Soresina et al, 2025 (5)	Italy	2004-2018	Prospective cohort	117	81	92/25	—	0.7(C), 6.5(M); —; 0-20(C), 0-54(M)	18(M); 22.4(M); 2.4-57.7	6; 9.5; 0-30
Imai et al., 2004 (45)	Japan	—	Retrospective cohort	50	15	33/17	—; —; 0.1-18	—; —; 0-1.5	—; —; 1-61	—; 9.3; —
Albert et al., 2010 (41)	Multiple	—	Retrospective cohort	173 (2)	25	0/173	—	—	11.5; —;2.0-74.6	—; 16.4; —
Khoreva et al., 2021 (27)	Russia	2012-2019	Retrospective cohort	67 (1)	0	54/13	—	—	1.3; —; 0-15	0.7; —; 0.1-1
Harfi et al., 1992 (48)	Saudi Arabia	1981-1990	Case series	5	2	5/0	Within 1^st^ week	0.3; 0.5; 0-1.1	3.5; 5.5; 1.5-15	3.3; 5.0; 1.4-14
Udomkittivorakul et al., 2022 (22)	Thailand	2008-2021	Case series	7	1	3/4	0.1; 17.0; 0-94.8	0.1; 1.5; 0.1-8	3.8; 5.0; 0.9-13.6	3.0; —; 0.1-13.0
Radl et al., 1976^c,d^ (51)	The Netherlands	—	Case series	3	0	3/0	—	0.6; 0.6; 0.4-0.8	3.5; 3.5; 3-4	1.5; 1.5; 1-2
Bildik et al., 2022 (24)	Turkey	1989-2014	Retrospective cohort	23	9	22/1	3; —; 0.5-117	1.3; —; 0.2-14.3	—	6.8; —; 0.8-15.2
Gök et al., 2025 (15)	Turkey	—	Retrospective cohort	16 (1)	12	12/4	—	0.7; 3.6; 0.2-15.5	—	—
Haskoloğlu et al., 2020 (29)	Turkey	1982-2019	Retrospective cohort	23	11	23/0	0.5; —; 0-7	2; —; 0.1-11	7; —; 2-26	8.5; —; 0.7-20
Sullivan et al., 1994 (3)	USA	—	Retrospective cohort	154	47	—	—	—; 1.8; 0-24.8	—; 9.6; 1-35	—
Lum et al., 1980^c,e^ (50)	USA	—	Case series	16	0	16/0	—	—	9.2; 10.2; 1.4-22	2.4; 5.7; 0.1-16.5
Mathew et al., 1995^c,d^ (46)	USA	1990-1994	Case series	12 (1)	—	—	—	1; —; 0.3-6.3	—	2.5; —; 0.5-4.5
Shin et al., 2012 (37)	USA	1990-2009	Retrospective cohort	47	47	47/0	—	—	—	5.4; —; 0.5-16.6
Burroughs et al., 2020 (30)	USA Canada	2005-2015	Retrospective cohort	129	129	86/43	—	—	—	4.5; —; 0.4-12.1
Perry et al., 1980^d,g^ (49)	USA Canada	1892-1979	Retrospective cohort	214	—	214/0	—	—	10; —; 1-36	—
Sun et al., 2024^c,d^ (17)	China	—	Case report	1	0	WAS Score: 3	0.5	—	1^h^	—
Lee et al., 2013^c,d^ (36)	South Korea	—	Case report	1	0	WAS Score: 2	At birth	—	4^h^	—
Faganello et al., 2008^c,d^ (44)	UK	—	Case report	1	0	WAS Score: 5		—	27^h^	—

CT, curative therapy; NA, not available; (C), patients with classic Wiskott-Aldrich syndrome phenotype (WAS score 3-5); (D), deceased patients; (M), patients with mild WAS phenotype (WAS score 1-2); (S), surviving patients.

^a^ Female patients.

^b^ Classic WAS, classic Wiskott-Aldrich syndrome phenotype (WAS score 3-5); mild WAS, mild Wiskott-Aldrich syndrome phenotype (WAS score 1-2).

^c^ Data from these studies were exclusively used for the qualitative analysis of age distribution of fatal hemorrhagic events.

^d^ Data from these studies were exclusively used for the qualitative analysis anatomical site distribution of fatal hemorrhagic events.

^e^ Although Lee (2008) (43) and Lum (1980) (50) overlapped with larger subsequent studies (Lee 2010 (40) and Perry 1980 (49), respectively), both were retained to inform the age distribution of fatal hemorrhage as they documented specific ages at death that were not recorded in their larger corresponding studies.

^f^ The subgroup numbers do not sum to the total due to a subset of patients with unclear classification.

^g^ Perry (1980) (49) was included for qualitative synthesis of anatomical sites of fatal hemorrhage but deemed ineligible for quantitative meta-analysis, as its insufficient granularity precluded the treatment-specific stratification essential for mortality estimates.

^h^ For case reports involving only a single patient, age at the last follow-up was documented as age at the time of the fatal hemorrhagic event.

Substantial variation was observed in the median age at diagnosis across included studies, with value ranging from birth to 11.7 years. The diagnosis of WAS was significantly delayed in regions (e.g., the Middle East and Southern Asia) with limited healthcare access and poor clinical recognition of the disease. Among the 31 included studies reporting the number of patients who received curative therapy, hematopoietic stem cell transplantation (HSCT) accounted for the vast majority of curative interventions, with only the study by Soresina et al. documenting 10 patients cured by gene therapy (**Table 1**) (5).

### Methodological quality

We used the JBI Critical Appraisal Checklist for cohort studies, case series, case reports, and case-control studies to assess the methodological quality of the included studies. Of the 24 cohort studies, 17 were classified as high quality, 7 as moderate quality (**Supplementary Table 6**). Among the 12 case series, 4 were rated as high quality and 8 as moderate quality (**Supplementary Table 7**). Of the 3 case reports, 2 were of high quality and 1 was of moderate quality (**Supplementary Table 8**). The single case-control study, with a 90% “yes” response on the JBI checklist, was judged to be of high quality (**Supplementary Table 9**).

### Cumulative incidence of bleeding in WAS patients

The pooled cumulative incidence of overall bleeding (18 studies, *n* = 681) was 93% (95% CI: 81-97), with substantial between-study heterogeneity (*I*^2^ = 57.2%, *τ*^2^ = 2.892, *Qp* = 0.001). Subgroup analysis identified clinical stage at data collection as a significant source of heterogeneity (test for subgroup differences: *p* = 0.0005), with proportions increasing from onset (66%, 95% CI: 56–75) to diagnosis (82%, 95% CI: 59–94) and end of follow-up (98%, 95% CI: 90–100) (**Table 2**; **Supplementary Figure 1**).

**Table 2:** Summary of meta-analytic results: characterization of bleeding phenotype and hemorrhage-related mortality in WAS.

Outcome	No. of studies (*k*)	No. of patients (*n*)	Pooled estimate (95% CI)	*I*^2^, %	τ^2^	*Q_p_* ^a^	*p*-value for subgroup differences
Cum Inc of overall bleeding	18	681	93% (81-97)	57.2	2.892	0.001	0.0005
Onset	4	97	66% (56-75)	0	0	0.644	
Diagnosis	4	332	82% (59-94)	79.5	0.992	0.002	
Follow-up	10	252	98% (90-100)	13	1.885	0.324	
Cum Inc of cutaneous bleeding	19	871	71% (52-85)	78.8	2.593	< 0.0001	0.0002
Onset	5	229	35% (29-41)	16.9	0	0.307	
Diagnosis	5	389	64% (44-80)	89.1	0.759	< 0.0001	
Follow-up	9	253	92% (64-98)	0	4.321	0.962	
Cum Inc of epistaxis	16	768	21% (13-33)	69.8	1.124	< 0.0001	0.0002
Onset	1	132	2% (0-5)	–	–	–	
Diagnosis	4	329	17% (12-23)	58.7	0.065	0.064	
Follow-up	11	307	28% (18-42)	56.8	0.697	0.010	
Cum Inc of GIB	20	725	40% (32-49)	75.1	0.442	< 0.0001	0.750
Onset	4	179	48% (18-80)	57.9	1.463	0.068	
Diagnosis	3	175	34% (18-56)	88.9	0.466	0.0001	
Follow-up	13	371	41% (32-52)	67.7	0.313	0.0002	
Cum Inc of ICH	17	859	8% (5-12)	64.9	0.621	0.0001	0.0008
Diagnosis	4	329	3% (1-5)	0	0	0.6491	
Follow-up	13	530	10% (7-16)	58.0	0.394	0.005	
Cum Inc of multisystem bleeding^b^	8	201	57% (41-72)	36.8	0.362	0.135	–
Cum Inc of severe bleeding^b^	10	636	19% (12-28)	81.5	0.556	< 0.0001	–
CSMR-H	–	–	–	–	–	–	–
Non-curative group	16	551	9.3% (6.1-13.8)	22.6	0.270	0.197	
Curative group	18	495	1.0% (0.4-2.4)	0	< 0.0001	1.000	
RR	12	666	0.07 (0.01-0.49)	3.3	0	0.992	
PMR-H in non-curative group	8	115	30% (22-39)	1.0	0	0.421	–

CSMR-H, cause-specific mortality rate from hemorrhage; Cum Inc, cumulative incidence; GIB, gastrointestinal bleeding; ICH, intracranial hemorrhage; PMR-H, proportional mortality ratio due to hemorrhage; RR, relative risk of fatal hemorrhage between curative and non-curative group; WAS, Wiskott-Aldrich syndrome.

^a^ *Q_p_* refers to *p*-value for Cochrane’s Q test.

^b^ The cumulative incidences of multisystem and severe bleeding were pooled exclusively at the final follow-up time point.

**Figure 2:**
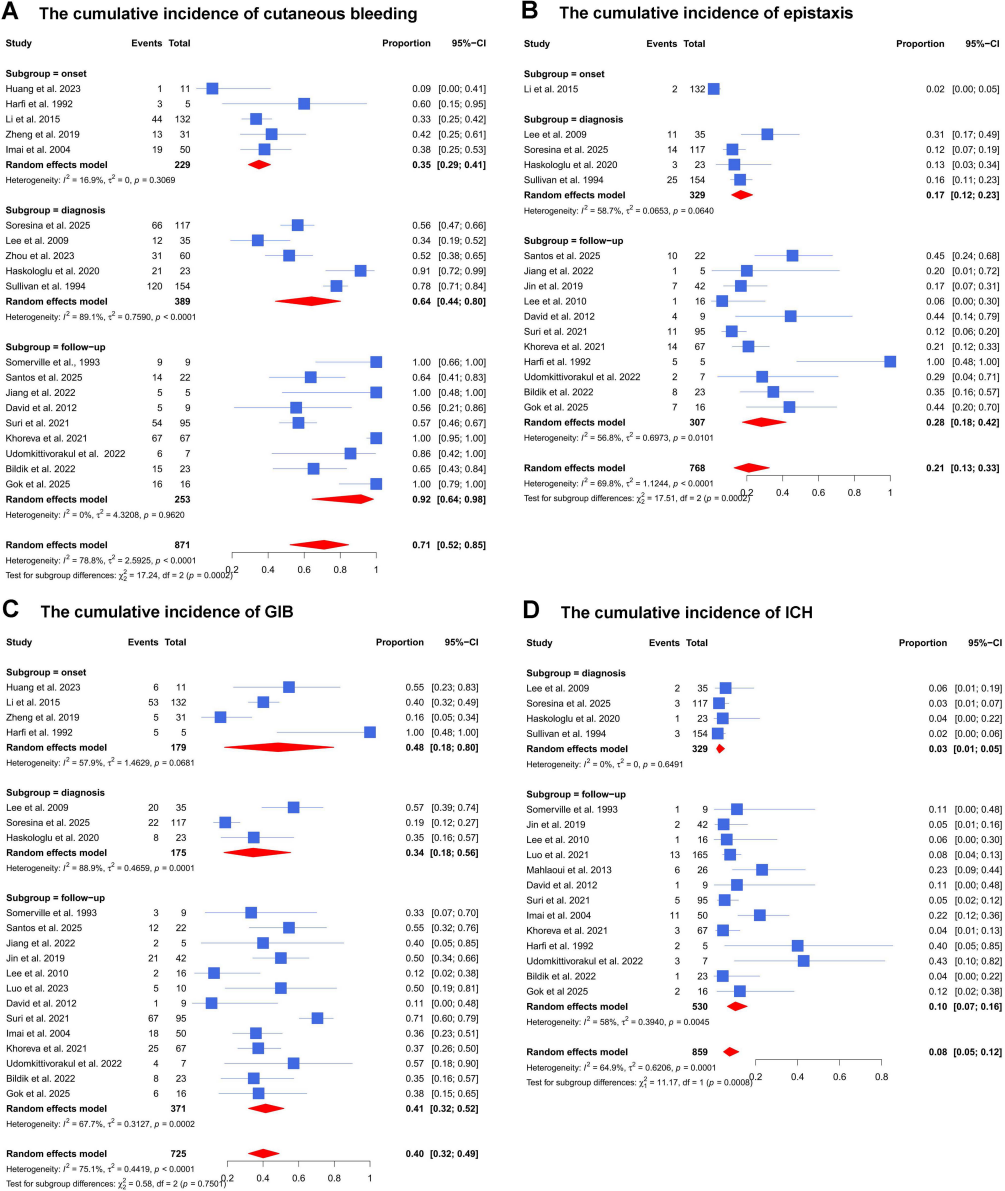
Meta-analyses on the cumulative incidence of site-specific bleeding in patients with WAS. Forest plots illustrate the pooled estimates for **(A)** cutaneous bleeding, **(B)** epistaxis, **(C)** gastrointestinal bleeding, and **(D)** intracranial hemorrhage.

The pooled cumulative incidences of site-specific bleeding manifestations were as follows: cutaneous bleeding (71%, 95% CI: 52-85), epistaxis (21%, 95% CI: 13-33), gastrointestinal bleeding (GIB) (40%, 95% CI: 32-49), and intracranial hemorrhage (ICH) (8%, 95% CI: 5-12). Significant between-study heterogeneity was observed for all outcomes (*Qp* ≤ 0.0001). Subgroup analysis demonstrated that clinical stage significantly influenced the pooled estimates of cutaneous bleeding, epistaxis, and ICH (i.e., the cumulative incidence increased significantly from onset to diagnosis, and through to the end of follow-up; test for subgroup differences: *p* < 0.001), but not GIB (*p* = 0.750). Notably, 66% of patients (95% CI: 56–75) presented with bleeding symptoms at onset, with cutaneous bleeding (35%, 95% CI: 29–41) and GIB (48%, 95% CI: 18-80) being the most common initial bleeding manifestations. At the end of follow-up, cutaneous bleeding had the highest pooled cumulative incidence (92%, 95% CI: 64-98), followed by GIB (41%, 95% CI: 32-52), epistaxis (28%, 95% CI: 18-42), and ICH (10%, 95% CI: 7-16) (**Table 2**; **Figures 2A–D**).

At the end of follow-up, the pooled cumulative incidences of multisystem bleeding (8 studies, *n* = 201) and severe bleeding (10 studies, *n* = 636) were 57% (95% CI: 41–72) and 19% (95% CI: 12–28), respectively (**Table 2**; **Supplementary Figures 2**, **3**). Significant between-study heterogeneity was observed for the cumulative incidence of severe bleeding (*I*^2^ = 81.5%, *τ*^2^ = 0.556, *Qp* < 0.0001). Meta-regression analyses revealed that the pooled estimate of severe bleeding was positively associated with the proportion of patients with a WAS score of 5 (*β* = 1.69, *p* = 0.0423) (**Supplementary Figure 4**) and negatively associated with study sample size (*β* = -0.011, *p* = 0.0042) (**Supplementary Figure 5**), although substantial residual heterogeneity persisted (*I*^2^ > 60%, *p* < 0.001) (**Supplementary Tables 10**, **11**).

Less frequent bleeding manifestations, including hematuria, hemoptysis, subgaleal or articular hematomas, and oral, otic, ocular, and renal hemorrhages, were also identified. However, the paucity of studies and insufficient patient numbers precluded a formal quantitative synthesis of these findings.

### Hemorrhage-related mortality in WAS patients

**Figure 3:**
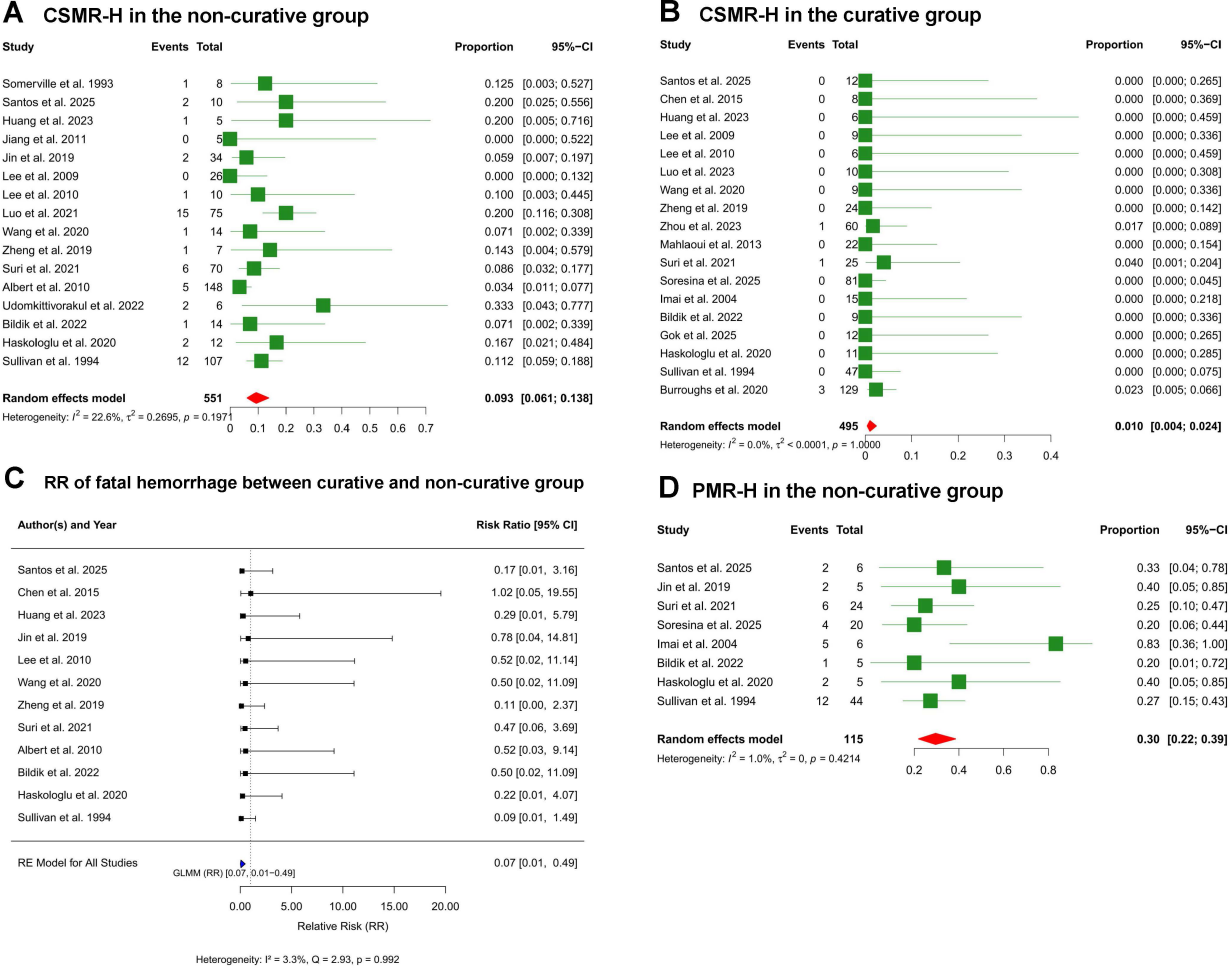
Meta-analyses on the mortality burden of hemorrhage in patients with WAS. Forest plots illustrate the pooled estimates for **(A)** cause-specific mortality rate from hemorrhage (CSMR-H) in the non-curative group, **(B)** CSMR-H in the curative group, **(C)** relative risk (RR) of fatal hemorrhage between curative and non-curative groups (estimated from 12 included studies that reported the CSMR-H in both groups), and **(D)** the proportional mortality ratio due to hemorrhage (PMR-H) in the non-curative group.

A total of 16 studies (*n* = 551) and 18 studies (*n* = 495) evaluated CSMR-H in non-curatively and curatively treated patients, respectively. In the non-curative group, the pooled proportion was 9.3% (95% CI: 6.1–13.8) (**Figure 3A**), whereas it was 1.0% (95% CI: 0.4–2.4) in the curative group (**Figure 3B**). Both analyses showed low heterogeneity (*I*^2^ = 22.6% and 0%; *Qp* = 0.197 and 1.000, respectively). 12 studies (*n* = 666) provided evaluable data to compare the risk of fatal hemorrhage between curative and non-curative group (**Supplementary Table 12**). The pooled results demonstrated that curative treatment was associated with a significantly lower risk of fatal hemorrhage compared with non-curative treatment (RR = 0.07, 95% CI: 0.01-0.49), with low heterogeneity observed across studies (*I*^2^ = 3.3%, *Qp* = 0.992) (**Figure 3C**). A total of 8 studies (*n* = 115) reported the PMR-H in non-curatively treated patients. The pooled estimate was 30% (95% CI: 22–39), with negligible heterogeneity observed across studies (*I*^2^ = 1.0%, *Qp* = 0.421) (**Table 2**; **Figure 3D**).

Detailed results of publication bias assessments and sensitivity analyses for all pooled estimates are provided in **Tables 3**, **4**, respectively (**Supplementary Figures 6**-**24**). Corresponding references for each pooled analysis are presented in **Supplementary Table 13**.

**Table 3:** Publication bias assessment results for pooled estimates of bleeding incidence and hemorrhage-related mortality in WAS.

Outcome	Evaluated group (no. of studies, *k*)	Funnel plot (visual inspection)	Egger’s test (*p*-value)	Interpretation
Cum Inc of overall bleeding	Follow-up subgroup (*k* = 10)^a^	Asymmetrical (**Supplementary Figure 6**)	0.044	Asymmetry likely a statistical artifact due to high extreme event rates (approaching 1), supported by non-significant Peter’s test (*p* = 0.575).
Cum Inc of cutaneous bleeding	Global/Subgroups	N/A	N/A	Not evaluated due to significant inter-subgroup heterogeneity and paucity of studies in each subgroup.
Cum Inc of epistaxis	Follow-up subgroup (*k* = 11)^a^	Symmetrical (**Supplementary Figure 7**)	0.248	No evidence of publication bias.
Cum Inc of GIB	Global (*k* = 20)	Symmetrical (**Supplementary Figure 8**)	0.987	No evidence of publication bias.
Cum Inc of ICH	Follow-up subgroup (*k* = 13)^a^	Asymmetrical (**Supplementary Figure 9**)	0.045	Asymmetry likely reflects small-study effects (smaller studies yielding higher estimates) rather than genuine bias.
Cum Inc of multisystem bleeding^b^	Global (*k* = 8)	N/A	N/A	Not evaluated due to the limited number of included studies (*k* < 10)
Cum Inc of severe bleeding^b^	Global (*k* = 10)	Symmetrical (**Supplementary Figure 10**)	0.583	No evidence of publication bias.
CSMR-H in curative group	Global (*k* = 18)	Asymmetrical (**Supplementary Figure 11**)	0.214	Asymmetry likely a statistical artifact due to high extreme event rates (approaching 0), supported by non-significant Peter’s test (*p* = 0.483).
CSMR-H in non-curative group	Global (*k* = 16)	Symmetrical (**Supplementary Figure 12**)	0.229	No evidence of publication bias.
RR	Global (*k* = 12)	Symmetrical (**Supplementary Figure 13**)	0.723	No evidence of publication bias.
PMR-H in non-curative group	Global (*k* = 8)	N/A	N/A	Not evaluated due to the limited number of included studies (*k* < 10)

CSMR-H, cause-specific mortality rate from hemorrhage; Cum Inc, cumulative incidence; Figure S, supplementary figure; GIB, gastrointestinal bleeding; ICH, intracranial hemorrhage; N/A, not applicable; PMR-H, proportional mortality ratio due to hemorrhage; RR, relative risk of fatal hemorrhage between curative and non-curative group; WAS, Wiskott-Aldrich syndrome.

^a^ Global test for publication bias was not performed for these estimates due to significant inter-subgroup difference; evaluations were restricted to subgroups with an adequate number of studies (*k* ≥ 10).

^b^ The cumulative incidences of multisystem and severe bleeding were pooled exclusively at the final follow-up time point.

**Table 4:** Leave-one-out sensitivity analysis results for pooled estimates of bleeding incidence and hemorrhage-related mortality in WAS.

Outcome	Subgroup	Overall robustness	Influential study identified	Effect on pooled proportion (95% CI) after exclusion	Effect on heterogeneity (*I*^2^) after exclusion
Cum Inc of overall bleeding (**Supplementary Figure 14**)	Onset	Robust^a^	None	Stable	Not significantly altered
	Diagnosis	Robust^a^	Zhou (2023) (18)	Stable	Decreased from 79.5% to 33.1%^b^
	Follow-up	Robust^a^	None	Stable	Not significantly altered
Cum Inc of cutaneous bleeding (**Supplementary Figure 15**)	Onset	Robust^a^	None	Stable	Not significantly altered
	Diagnosis	Robust^a^	None	Stable	Not significantly altered
	Follow-up	Sensitive	Suri (2021) (25) Khoreva (2021) (27)	Fluctuated between 67% (60-73) and 87% (80-91)^b^	Not significantly altered
Cum Inc of epistaxis (**Supplementary Figure 16**)	Diagnosis	Robust^a^	Lee (2009) (42)	Stable	Decreased from 58.7% to 0%^c^
	Follow-up	Robust^a^	None	Stable	Not significantly altered
Cum Inc of GIB (**Supplementary Figure 17**)	Onset	Robust^a^	None	Stable	Not significantly altered
	Diagnosis	Sensitive	Soresina (2025) (5)	Fluctuated between 21% (15-29) and 48% (36-61)^d^	Not significantly altered
	Follow-up	Robust^a^	Suri (2021) (25)	Stable	Decreased from 67.7% to 11.9%^b^
Cum Inc of ICH (**Supplementary Figure 18**)	Diagnosis	Robust^a^	None	Stable	Not significantly altered
	Follow-up	Robust^a^	None	Stable	Not significantly altered
Cum Inc of multisystem bleeding (**Supplementary Figure 19**)	Global	Robust^a^	None	Stable	Not significantly altered
Cum Inc of severe bleeding (**Supplementary Figure 20**)^e^	Global	Robust^a^	None	Stable	Not significantly altered
CSMR-H in curative group (**Supplementary Figure 21**)^e^	Global	Sensitive	Burroughs (2020) (30)	Decreased from 1.0% (0.4-2.4) to 0.5% (0.1-2.2)^f^	Not significantly altered
CSMR-H in non-curative group (**Supplementary Figure 22**)	Global	Robust^a^	None	Stable	Not significantly altered
RR (**Supplementary Figure 23**)	Global	Robust^a^	None	Stable	Not significantly altered
PMR-H in non-curative group (**Supplementary Figure 24**)	Global	Robust^a^	None	Stable	Not significantly altered

CSMR-H, cause-specific mortality rate from hemorrhage; Cum Inc, cumulative incidence; Figure S, supplementary figure; GIB, gastrointestinal bleeding; ICH, intracranial hemorrhage; PMR-H, proportional mortality ratio due to hemorrhage; RR, relative risk of fatal hemorrhage between curative and non-curative group; WAS, Wiskott-Aldrich syndrome.

^a^ “Robust” indicates that the omission of any single study did not significantly alter the pooled estimate or its statistical significance.

^b^ Interpretations are presented in “Discussion”.

^c^ A result potentially exaggerated by the paucity of studies available for this subgroup.

^d^ Driven largely by the small number of studies in this subgroup and the disproportionate weight of large-sample study by Soresina et al.

^e^ The cumulative incidences of multisystem and severe bleeding were pooled exclusively at the final follow-up time point.

^f^ Burroughs (2020) (30) was a large-sample study which potentially overestimated the mortality for this cohort.

### Age and anatomical site distributions of fatal hemorrhagic events

**Figure 4:**
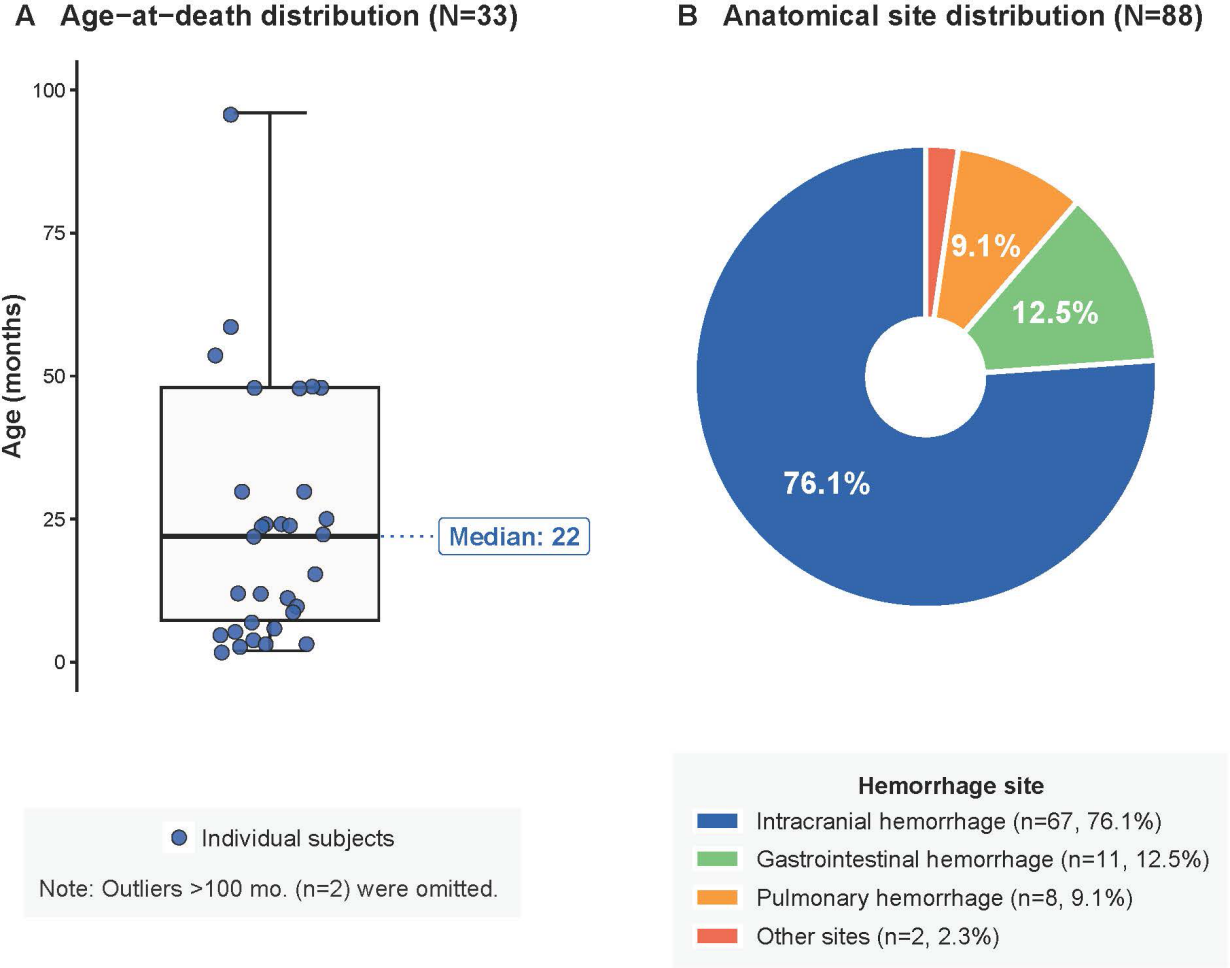
Characteristics of fatal hemorrhagic events. **(A)** Box plot illustrating the age-at-death distribution (*n* = 33). **(B)** Doughnut chart showing the anatomical site distribution (*n* = 88).

A total of 33 non-curatively treated patients who succumbed to hemorrhage were identified across 21 studies with documented age-at-death, which ranged widely from 2 months to 74.6 years. The age distribution of fatal hemorrhagic events was markedly skewed toward early childhood, with a median age-at-death of 22 months (**Figure 4A**). Although the preponderance of fatalities occurred within the first few years of life, late-onset events were documented in two notable outliers aged 27 and 74.6 years (**Supplementary Table 14**).

Analysis of 88 fatal hemorrhagic events among non-curatively treated patients across 18 studies revealed that ICH was the predominant cause of death, accounting for over three-quarters of these cases (n=67, 76.1%) (**Supplementary Table 15**). GIB and pulmonary hemorrhages followed, accounting for 12.5% (n = 11) and 9.1% (n = 8), respectively. Rare fatal hemorrhagic events at other sites (retroperitoneal hemorrhage and aortic rupture) were documented in only 2 patients (**Figure 4B**) (44, 46).

## Discussion

This study represents the most comprehensive systematic review and meta-analysis to date on bleeding incidence and hemorrhage-related mortality in WAS patients. Bleeding symptoms are present in most patients at onset and become nearly ubiquitous during follow-up, with almost all patients reporting a history of hemorrhagic events at the end of available observation. Notably, we provide the first pooled estimate of CSMR-H in non-curatively treated patients: approximately 9%, accounting for nearly one-third of all-cause mortality. Critically, curative intervention brings the CSMR-H down to around 1%, corresponding to a striking RR of 0.07.

These findings have important clinical implications for WAS diagnosis and management. Bleeding manifestations are the initial symptom in over half of patients, with cutaneous bleeding and GIB being the most common. WAS should therefore be considered in patients presenting with isolated cutaneous bleeding or GIB to facilitate early recognition. Clinicians should remain vigilant for hemorrhagic complications, particularly ICH, even in patients without bleeding at diagnosis, given the lifelong risk. In addition, infants and young children should be identified a high-risk population, as fatal hemorrhages occur early in non-curatively treated patients. Intensified monitoring and proactive intervention are warranted in this cohort. Furthermore, the dramatic reduction of CSMR-H in curatively treated patients underscores that non-curative modalities only serve as a temporary bridging strategy and fail to eliminate the risk of fatal hemorrhage. Consequently, timely implementation of curative therapy is imperative for high-risk patients.

Interestingly, no stepwise increase was observed in the pooled cumulative incidence of GIB across the disease trajectory, with a numerically higher pooled proportion at disease onset (48%) than that at the end of follow-up (41%). This unexpected finding is likely explained by age-specific pathophysiology and detection bias. First, WAS typically presents in infancy, when intestinal barrier immaturity and increased susceptibility to enteric infections and allergies, combined with severe thrombocytopenia, confer a high intrinsic GIB risk (53). This intrinsic susceptibility wanes with age as the intestinal barrier matures and atopic triggers diminish. Second, ascertainment bias plays a more critical role: universal diaper use in infancy ensures near-complete documentation of even minor hematochezia (54), whereas low-grade GIB becomes highly occult after toilet training. Studies in which cumulative GIB events were reported at the end of follow-up therefore predominantly capture severe, clinically overt GIB episodes, leading to an underestimation of the true GIB burden.

Our analysis identified a significant positive correlation between the proportion of patients with a WAS score of 5 and the cumulative incidence of severe bleeding (*p* = 0.0423). We hypothesize that autoantibody production and the associated vasculitis may trigger and exacerbate bleeding by compromising microvascular integrity (55). Given the limited number of studies available for this meta-regression (*k* = 10) and the variable ascertainment methods for severe bleeding across the included studies, this finding should be considered preliminary. Prospective studies be needed to confirm whether autoimmunity represents an independent risk factor for severe bleeding in WAS. Nevertheless, conventional phenotypic severity scoring for WAS has notable limitations, as it only reflects disease status at a single time point and cannot predict subsequent progression or late-onset life-threatening complications. Recently, a global multicenter study involving 577 patients by Vallée et al. demonstrating that genotype serves as a superior prognostic biomarker. The researchers classified *WAS* pathogenic variants into two groups: class I includes missense variants located in exons 1 and 2 or the intronic hotspot c.559 + 5G>A, associated with residual WASP expression and a milder disease phenotype; class II comprises all remaining pathogenic variants, which result in absent or non-functional WASP and markedly poorer clinical outcomes (6). While Vallée et al. demonstrated that class II variants carry a significantly elevated risk of severe bleeding, we were unable to perform a meta-regression between the class II/class I variant ratio and severe bleeding due to incomplete genotypic data in several original studies. We therefore recommend that all future clinical studies report detailed genotype information for all enrolled patients whenever possible, to facilitate comparative analysis using the standardized class I/II genotype classification.

In addition, we identified a significant negative correlation between sample size and the reported cumulative incidence of severe bleeding (*p* = 0.0042). This association reflects a systematic selection bias: small-sample studies often enrich for the severe, refractory cases encountered in tertiary care. In contrast, large-scale national registries capture a more balanced clinical spectrum, including patients with milder phenotype, and thus yield more accurate estimates (56). To avoid the overestimation bias of small-scale cohorts, future epidemiological studies of WAS should prioritize the use of multicenter, or national registry-based datasets. Regrettably, we were unable to analyze the correlation between severe bleeding events and platelet counts, as this dynamically fluctuating indicator was reported in highly heterogeneous formats across studies, precluding uniform quantification for meta-regression analysis. Future research should standardize platelet count reporting by clarifying measurement time points and adopting consistent statistical indices.

Subgroup analyses by clinical stage did not fully resolve the observed heterogeneity. Significant heterogeneity remained for overall bleeding at diagnosis (*I*^2^ = 79.5%), with sensitivity analysis identifying Zhou et al. (2023) as the primary source (*I*^2^ = 33.1% after exclusion). This discrepancy likely stems from variations in diagnostic delay: While Zhou et al. reported an early median age at diagnosis (0.4 years), other studies (Suri et al., Haskoloğlu et al., and Sullivan et al.) conducted in regions or eras with more limited healthcare access and lower clinical awareness of WAS showed much later diagnoses (1 to 2 years) (3, 25, 29). This delay also accounts for the heterogeneity seen in cutaneous bleeding, where late-diagnosed cohorts reported higher proportions. These findings underscore the critical importance of early diagnosis and the timely initiation of standardized care, as many new-onset patients experience bleeding events without standardized management due to delayed diagnosis.

Significant residual heterogeneity was also observed in the follow-up subgroup of GIB (*I*^2^ = 67.7%, *p* = 0.0002), primarily driven by Suri et al. (2021) (*I*^2^ = 11.9% after exclusion). The divergent bleeding proportions between Suri et al. (57% cutaneous bleeding vs. 71% GIB) and Khoreva et al. (100% cutaneous bleeding vs. 37% GIB) highlights the impact of study design and reporting bias. Khoreva et al. studied romiplostim efficacy in refractory bleeding patients, using the validated WHO bleeding scale to capture even minor cutaneous manifestations (27). In contrast, the multicenter retrospective study in India by Suri et al. suffered from under-reporting of minor pre-diagnostic symptoms such as petechiae due to poor awareness, while more overt GIB events were consistently documented (25). This initial reporting bias at diagnosis (20% cutaneous bleeding vs 49% GIB) carried over into follow-up data, resulting in a lower reported proportion of cutaneous bleeding (57%) but a higher reported proportion of GIB (71%). Additional sources of residual heterogeneity include ethnic differences and variable follow-up durations.

Eighty-five percent of fatal hemorrhages occurred before the age of four, a finding primarily attributable to the fact that most high-risk patients undergo HSCT by this age, thereby significantly reducing the risk of fatal bleeding thereafter. Nevertheless, we identified two fatal events in adulthood: one in a 27-year-old (aortic rupture secondary to vasculitis) and another in a 74-year-old (unspecified site). These cases underscore that fatal complications can emerge throughout life, even in those with milder phenotypes, reinforcing the necessity of lifelong regular follow-up for all WAS patients, regardless of early-life disease severity (5, 57).

Several limitations should be considered when interpreting our findings. First, as many studies failed to report the median age of patients corresponding to their findings, the pooled cumulative incidence was only coarsely categorized by clinical milestones (i.e., at onset, at diagnosis, and at the end of follow-up). This precluded quantitative assessment of the heterogeneity arising from variations in disease duration and evaluation of how cumulative incidence evolves with disease progression. To improve the comparability of future evidence, time-to-event data in longitudinal studies of WAS is strongly recommended. Second, the inconsistent grading systems for bleeding events across studies may have introduced heterogeneity into the analysis of severe bleeding. Merely 3 studies (Li et al., 2015; Khoreva et al., 2021; Gök et al., 2025) recorded the specific number of patients with mild, moderate and severe bleeding events respectively. We were therefore unable to perform a meta-analysis of the cumulative incidence of mild and moderate bleeding events. Third, as the median follow-up duration (or median age at last follow-up) across most included studies was under a decade, our pooled estimates represent medium-term outcomes. To better understand the evolution of bleeding manifestations and the long-term survival into adulthood, especially among those with the attenuated phenotype, multi-institutional prospective cohorts with extended follow-up are warranted. Finally, limited to observational studies, this meta-analysis is unable to assess the performance of currently popular interventions such as thrombopoietin receptor agonists and gene therapy for WAS. Research on these approaches consists predominantly of clinical trials, with only a small body of published primary studies to date. We anticipate that these key clinical questions can be fully explored in future meta-analyses of interventional studies once a larger dataset has been accumulated.

## Conclusions

In conclusion, this systematic review and meta-analysis demonstrates that bleeding complications are virtually ubiquitous and exhibit a progressive trajectory in patients with WAS; thus, heightened clinical vigilance and continuous monitoring for hemorrhagic complications are critical. Since fatal hemorrhages occur primarily in early childhood and are largely preventable through curative intervention, prompt initiation of curative therapy is essential to improve survival in high-risk patients.

## Data Availability

The original contributions presented in the study are included in the article/**Supplementary Material**. Further inquiries can be directed to the corresponding authors.
