# Supplementary material for: The spectrum of bleeding in Wiskott-Aldrich syndrome: a systematic review and meta-analysis of incidence and mortality
Source: Front Immunol. 2026 Jul 6;17:1854833. doi: 10.3389/fimmu.2026.1854833 (PMC13381193; doi:10.3389/fimmu.2026.1854833)
Supplement: Supplementary file 1 [file DataSheet1.docx]

**Supplementary data**

**Table of Contents**

[Supplementary Table 1 Meta-analysis of observational studies in epidemiology (MOOSE) checklist. 2](#_Toc231078443)

[Supplementary Table 2 Detailed search strategies and search results. 3](#_Toc231078444)

[Supplementary Table 3 Predefined data extraction items and operational definitions of key clinical outcomes. 4](#_Toc231078445)

[Supplementary Table 4 Detailed protocol for handling overlapping data. 5](#_Toc231078446)

[Supplementary Table 5 Participating Institutions/Collaborating Centers of included studies. 7](#_Toc231078447)

[Supplementary Table 6 Quality assessment of included cohort studies. 9](#_Toc231078448)

[Supplementary Table 7 Quality assessment of included case series. 9](#_Toc231078449)

[Supplementary Table 8 Quality assessment of included case reports. 10](#_Toc231078450)

[Supplementary Table 9 Quality assessment of included case-control study. 10](#_Toc231078451)

[Supplementary Table 10 Meta-regression results for heterogeneity sources of cumulative incidence of severe bleeding. 11](#_Toc231078452)

[Supplementary Table 11 Raw data of studies included in the meta-regression analysis of severe bleeding incidence. 11](#_Toc231078453)

[Supplementary Table 12 Original data from 12 included studies for relative risk analysis of fatal hemorrhage by treatment group in WAS. 11](#_Toc231078454)

[Supplementary Table 13 Corresponding references for each pooled analysis. 12](#_Toc231078455)

[Supplementary Table 14 Summary of studies reporting age at fatal hemorrhagic event. 13](#_Toc231078456)

[Supplementary Table 15 Anatomical site distribution of fatal hemorrhagic events. 13](#_Toc231078457)

[Supplementary Figure 1 14](#_Toc231078458)

[Supplementary Figure 2 15](#_Toc231078459)

[Supplementary Figure 3 15](#_Toc231078460)

[Supplementary Figure 4 16](#_Toc231078461)

[Supplementary Figure 5 17](#_Toc231078462)

[Supplementary Figure 6 18](#_Toc231078463)

[Supplementary Figure 7 19](#_Toc231078464)

[Supplementary Figure 8 20](#_Toc231078465)

[Supplementary Figure 9 21](#_Toc231078466)

[Supplementary Figure 10 22](#_Toc231078467)

[Supplementary Figure 11 23](#_Toc231078468)

[Supplementary Figure 12 24](#_Toc231078469)

[Supplementary Figure 13 25](#_Toc231078470)

[Supplementary Figure 14 26](#_Toc231078471)

[Supplementary Figure 15 27](#_Toc231078472)

[Supplementary Figure 16 28](#_Toc231078473)

[Supplementary Figure 17 29](#_Toc231078474)

[Supplementary Figure 18 30](#_Toc231078475)

[Supplementary Figure 19 31](#_Toc231078476)

[Supplementary Figure 20 31](#_Toc231078477)

[Supplementary Figure 21 32](#_Toc231078478)

[Supplementary Figure 22 32](#_Toc231078479)

[Supplementary Figure 23 33](#_Toc231078480)

[Supplementary Figure 24 34](#_Toc231078481)

[References 35](#_Toc231078482)

# Supplementary Table 1 Meta-analysis of observational studies in epidemiology (MOOSE) checklist.

| **A Proposed Reporting Checklist for Authors, Editors, and Reviewers of Meta-analyses of Observational Studies** | | |
| --- | --- | --- |
| **Reporting criteria** | **Reported (Yes/No)** | **Location where item is reported** |
| **Reporting of background should include** | | |
| Problem definition | Yes | Page 3 |
| Hypothesis statement | Yes | Page 3 |
| Description of study outcome(s) | Yes | Page 3 |
| Type of exposure or intervention used | Yes | Page 3 |
| Type of study designs used | Yes | Page 4 |
| Study population | Yes | Page 4 |
| **Reporting of search strategy should include** | | |
| Qualifications of searchers (eg, librarians and investigators) | Yes | Page 4 |
| Search strategy, including time period included in the synthesis and keywords | Yes | Page 4 |
| Effort to include all available studies, including contact with authors | Yes | Page 4 |
| Databases and registries searched | Yes | Page 4 |
| Search software used, name and version, including special features used (eg, explosion) | Yes | Page 4 |
| Use of hand searching (eg, reference lists of obtained articles) | Yes | Page 4 |
| List of citations located and those excluded, including justification | Yes | Page 7 |
| Method of addressing articles published in languages other than English | Yes | Page 5 |
| Method of handling abstracts and unpublished studies | Yes | Page 7 |
| Description of any contact with authors | No | - |
| **Reporting of methods should include** | | |
| Description of relevance or appropriateness of studies assembled for assessing the hypothesis to be tested | Yes | Page 4 and 7 |
| Rationale for the selection and coding of data (eg, sound clinical principles or convenience) | Yes | Page 4-5 |
| Documentation of how data were classified and coded (eg, multiple raters, blinding, and interrater reliability) | Yes | Page 5 |
| Assessment of confounding (eg, comparability of cases and controls in studies where appropriate) | Yes | Page 5 |
| Assessment of study quality, including blinding of quality assessors; stratification or regression on possible predictors of study results | Yes | Page 5 |
| Assessment of heterogeneity | Yes | Page 6 |
| Description of statistical methods (eg, complete description of fixed or random effects models, justification of whether the chosen models account for predictors of study results, dose-response models, or cumulative meta-analysis) in sufficient detail to be replicated | Yes | Page 6 |
| Provision of appropriate tables and graphics | Yes | Page 22-29 |
| **Reporting of results should include** | | |
| Graphic summarizing individual study estimates and overall estimate | Yes | Page 8-10, and 29 |
| Table giving descriptive information for each study included | Yes | Page 22-25 |
| Results of sensitivity testing (eg, subgroup analysis) | Yes | Page 10 and 28 |
| Indication of statistical uncertainty of findings | Yes | Page 8-10, and 26 |
| **Reporting of discussion should include** | | |
| Quantitative assessment of bias (eg, publication bias) | Yes | Page 10 and 27 |
| Justification for exclusion (eg, exclusion of non–English-language citations) | Yes | Page 5, 7, and 29 |
| Assessment of quality of included studies | Yes | Page 7-8 |
| **Reporting of conclusions should include** | | |
| Consideration of alternative explanations for observed results | Yes | Page 11-15 |
| Generalization of the conclusions (ie, appropriate for the data presented and within the domain of the literature review) | Yes | Page 16 |
| Guidelines for future research | Yes | Page 12-15 |
| Disclosure of funding source | Yes | Page 16 |

# Supplementary Table 2 Detailed search strategies and search results.

| Search strategy | Search result |
| --- | --- |
| PubMed (Date: *26 December 2025*) | |
| ((Hemorrhage[MeSH Terms]) OR (Hemorrhages[Title/Abstract] OR Bleeding[Title/Abstract] OR Blood Loss[Title/Abstract] OR Epistaxis[Title/Abstract] OR Hematemesis[Title/Abstract] OR Melena[Title/Abstract] OR Hemarthrosis[Title/Abstract] OR Hematocele[Title/Abstract] OR Hematoma[Title/Abstract] OR Hematuria[Title/Abstract] OR Hemobilia[Title/Abstract] OR Hemoperitoneum[Title/Abstract] OR Hemoptysis[Title/Abstract] OR Hemothorax[Title/Abstract] OR petechia[Title/Abstract] OR ecchymosis[Title/Abstract])) AND ((Wiskott-Aldrich syndrome[MeSH Terms]) OR (Wiskott Aldrich Syndrome[Title/Abstract] OR Aldrich Syndrome[Title/Abstract] OR Eczema-Thrombocytopenia-Immunodeficiency Syndrome[Title/Abstract] OR Eczema Thrombocytopenia Immunodeficiency Syndrome[Title/Abstract] OR Eczema-Thrombocytopenia-Immunodeficiency Syndromes[Title/Abstract] OR Wiskott Syndrome[Title/Abstract] OR Wiskott Syndromes[Title/Abstract])) | n=196 |
| Embase (Date: 26 December 2025) | |
| ((‘wiskott aldrich syndrome’/exp) OR (‘Wiskott-Aldrich syndrome’:ab,ti OR ‘Aldrich Syndrome’:ab,ti OR ‘Eczema-Thrombocytopenia-Immunodeficiency Syndrome’:ab,ti OR ‘Eczema Thrombocytopenia Immunodeficiency Syndrome’:ab,ti OR ‘Eczema-Thrombocytopenia-Immunodeficiency Syndromes’:ab,ti OR ‘Wiskott Syndrome’:ab,ti OR ‘Wiskott Syndromes’:ab,ti  )) AND ((‘Bleeding’/exp) OR (‘Hemorrhage’:ab,ti OR ‘Hemorrhages’:ab,ti OR ‘Blood Loss’:ab,ti OR ‘Epistaxis’:ab,ti OR ‘Hematemesis’:ab,ti OR ‘Melena’:ab,ti OR ‘Hemarthrosis’:ab,ti OR ‘Hematocele’:ab,ti OR ‘Hematoma’:ab,ti OR ‘Hematuria’:ab,ti OR ‘Hemobilia’:ab,ti OR ‘Hemoperitoneum’:ab,ti OR ‘Hemoptysis’:ab,ti OR ‘Hemothorax’:ab,ti OR ‘petechia’:ab,ti OR ‘ecchymosis’:ab,ti)) | n=674 |
| Web of science (Date: 26 December 2025) | |
| (TS=(“Wiskott Aldrich syndrome” OR “Wiskott-Aldrich syndrome” OR “Aldrich Syndrome” OR “Eczema-Thrombocytopenia-Immunodeficiency Syndrome” OR “Eczema Thrombocytopenia Immunodeficiency Syndrome” OR “Eczema-Thrombocytopenia-Immunodeficiency Syndromes” OR “Wiskott Syndrome” OR “Wiskott Syndromes”)) AND (TS=(“Hemorrhage” OR “Hemorrhages” OR “Bleeding” OR “Blood Loss” OR “Epistaxis” OR “Hematemesis” OR “Melena” OR “Hemarthrosis” OR “Hematocele” OR “Hematoma” OR “Hematuria” OR “Hemobilia” OR “Hemoperitoneum” OR “Hemoptysis” OR “Hemothorax” OR “petechia” OR “ecchymosis”)) | n=163 |
| Cochrane library (Date: 26 December 2025) | |
| #1 MeSH descriptor: [Wiskott-Aldrich Syndrome] explode all trees  #2 (‘Aldrich Syndrome’ OR ‘Eczema Thrombocytopenia Immunodeficiency Syndrome’ OR ‘Wiskott Syndrome’ OR ‘Wiskott Syndromes’):ti,ab,kw  #3 MeSH descriptor: [Hemorrhage] explode all trees  #4 (‘Hemorrhages’ OR ‘Bleeding’ OR ‘Blood Loss’ OR ‘Epistaxis’ OR ‘Hematemesis’ OR ‘Melena’ OR ‘Hemarthrosis’ OR ‘Hematocele’ OR ‘Hematoma’ OR ‘Hematuria’ OR ‘Hemobilia’ OR ‘Hemoperitoneum’ OR ‘Hemoptysis’ OR ‘Hemothorax’ OR ‘petechia’ OR ‘ecchymosis’):ti,ab,kw  #5 (#1 OR #2) AND (#3 OR #4) | n=8 |
| Scopus (Date: 26 December 2025) | |
| ( TITLE-ABS-KEY ( "Wiskott-Aldrich syndrome" OR "Wiskott Aldrich syndrome" OR "Aldrich Syndrome" OR "Eczema-Thrombocytopenia-Immunodeficiency Syndrome" OR "Eczema Thrombocytopenia Immunodeficiency Syndrome" OR "Eczema-Thrombocytopenia-Immunodeficiency Syndromes" OR "Wiskott Syndrome" OR "Wiskott Syndromes" ) ) AND ( TITLE-ABS-KEY ( "Hemorrhage" OR "Hemorrhages" OR "Bleeding" OR "Blood Loss" OR "Epistaxis" OR "Hematemesis" OR "Melena" OR "Hemarthrosis" OR "Hematocele" OR "Hematoma" OR "Hematuria" OR "Hemobilia" OR "Hemoperitoneum" OR "Hemoptysis" OR "Hemothorax" OR "petechia" OR "ecchymosis" ) ) | n=494 |

# Supplementary Table 3 Predefined data extraction items and operational definitions of key clinical outcomes.

| Category No. | Data extraction category | Detailed items | Operational definitions |
| --- | --- | --- | --- |
| 1 | Study characteristics | 1. First author and year of publication  2. Enrollment period  3. Study location (country and participating institutions)  4. Study design  5. Follow-up period | **1. Site-specific bleeding manifestations**: including cutaneous bleeding, epistaxis, gastrointestinal bleeding (GIB), and intracranial hemorrhage (ICH).  **2. Severe bleeding**: defined according to the criteria specified in each original study, generally understood as hemorrhage requiring urgent medical intervention.  **3. Multisystem bleeding**: operationalized as bleeding involving two or more organ systems. For this variable, data were extracted directly if a study reported the number of patients with multisystem involvement. Alternatively, where studies provided detailed, patient-level listings of bleeding sites (e.g., documenting both cutaneous bleeding and GIB in an individual), the number of patients with multisystem involvement was manually calculated. |
| 2 | Patient Baseline Characteristics | 1. Total sample size of the study cohort  2. Number of female patients  3. Number of patients receiving curative treatment  4. Ages at key timepoints (age at onset, diagnosis, and last follow-up) |  |
| 3 | Core Clinical Outcomes | 1. number of patients with bleeding manifestations (including overall, site-specific, severe and multisystem bleeding)  2. Total number of fatal cases, including those attributable to hemorrhage  3. Patient age and anatomical site of each fatal hemorrhagic event. |  |

# Supplementary Table 4 Detailed protocol for handling overlapping data across included studies.

| Outcome | Overlap scenario | Handling approach | Overlapping studies | Selected for analysis | Removed due to overlap |
| --- | --- | --- | --- | --- | --- |
| Cumulative incidence of overall bleeding | Multiple publications from a single center with overlapping enrollment periods | The publication providing the largest sample size was prioritized for inclusion | Jin et al., 2019 (42)  Zhou et al, 2023 (60) | Zhou et al, 2023 (60) | Jin et al., 2019 (42) |
| Cumulative incidence of cutaneous bleeding | Multiple publications from a single center with overlapping enrollment periods | The publication providing the largest sample size was prioritized for inclusion | Jin et al., 2019 (42)  Zhou et al, 2023 (60) | Zhou et al, 2023 (60) | Jin et al., 2019 (42) |
| Cumulative incidence of epistaxis | - | - | - | - | - |
| Cumulative incidence of gastrointestinal bleeding | **-** | **-** | **-** | **-** | **-** |
| Cumulative incidence of intracranial hemorrhage | - | - | - | - | - |
| Cumulative incidence of severe bleeding | Multiple multicenter studies sharing one or more participating centers with overlapping enrollment periods | The publication with the largest number of participating centers and patients  was prioritized for inclusion | Albert et al., 2010 (173)  Imai et al., 2004 (50) | Albert et al., 2010 (173) | Imai et al., 2004 (50) |
|  | Multiple publications from a single center with overlapping enrollment periods | The publication providing the largest sample size was prioritized for inclusion | Lee et al., 2008 (11)  Lee et al., 2010 (16) | Lee et al., 2010 (16) | Lee et al., 2008 (11) |
| Cumulative incidence of multisystem bleeding | - | - | - | - | - |
| Cause-specific mortality rate from hemorrhage in curatively  treated patients | Multiple multicenter studies sharing one or more participating centers with overlapping enrollment periods | The publication with the largest number of participating centers and patients  was prioritized for inclusion | Albert et al., 2010 (25)  Burroughs et al, 2020 (129) | Burroughs et al, 2020 (129) | Albert et al., 2010 (25) |
|  | A multicenter study had overlapping enrollment periods with a single-center study from its collaborating centers | The multicenter study was prioritized for inclusion | Shin et al., 2012 (47)  Burroughs et al, 2020 (129) | Burroughs et al, 2020 (129) | Shin et al., 2012 (47) |
|  | Multiple publications from a single center with overlapping enrollment periods | The publication providing the largest sample size was prioritized for inclusion | Jin et al., 2019 (8)  Zhou et al., 2023 (60) | Zhou et al., 2023 (60) | Jin et al., 2019 (8) |
|  |  |  | Lee et al., 2008 (5)  Lee et al., 2010 (6) | Lee et al., 2010 (6) | Lee et al., 2008 (5) |
| Cause-specific mortality rate from hemorrhage in non-curatively  treated patients | Multiple multicenter studies sharing one or more participating centers with overlapping enrollment periods | The publication with the largest number of participating centers and patients  was prioritized for inclusion | Albert et al., 2010 (148)  Soresina et al, 2025 (36)  Imai et al., 2004 (35) | Albert et al., 2010 (148) | Soresina et al, 2025 (36)  Imai et al., 2004 (35) |
|  | Multiple publications from a single center with overlapping enrollment periods | The publication providing the largest sample size was prioritized for inclusion | Lee et al., 2008 (6)  Lee et al., 2010 (10) | Lee et al., 2010 (10) | Lee et al., 2008 (6) |
|  |  |  | Chen et al., 2015 (45)  Luo et al., 2021 (75) | Luo et al., 2021 (75) | Chen et al., 2015 (45) |
|  | A multicenter study had overlapping enrollment periods with a single-center study from its collaborating centers | The multicenter study was prioritized for inclusion | Sullivan et al., 1994 (107)  Lum et al., 1980 (16) | Sullivan et al., 1994 (107) | Lum et al., 1980 (16) |
| The pooled relative risk for fatal hemorrhage between the curative and non-curative groups | Multiple multicenter studies sharing one or more participating centers with overlapping enrollment periods | The publication with the largest number of participating centers and patients  was prioritized for inclusion | Albert et al., 2010 (173)  Soresina et al, 2025 (117)  Imai et al., 2004 (50) | Albert et al., 2010 (173) | Soresina et al, 2025 (117)  Imai et al., 2004 (50) |
| The proportional mortality ratio due to hemorrhage in non-curatively treated patients | Multiple multicenter studies sharing one or more participating centers with overlapping enrollment periods | The publication with the largest number of participating centers and patients  was prioritized for inclusion | Albert et al., 2010 (10)  Soresina et al, 2025 (20)  Imai et al., 2004 (6) | Soresina et al, 2025 (20)  Imai et al., 2004 (6) | Albert et al., 2010 (10) |
|  | A multicenter study had overlapping enrollment periods with a single-center study from its collaborating centers | The multicenter study was prioritized for inclusion | Sullivan et al., 1994 (42)  Lum et al., 1980 (10) | Sullivan et al., 1994 (42) | Lum et al., 1980 (10) |
| Anatomical site distribution of fatal hemorrhage  (pulmonary hemorrhage) | A global multicenter study had overlapping enrollment periods with several single-country studies conducted at its collaborating centers | The global multicenter study was prioritized for exclusion* | Albert et al., 2010 [1]  Perry et al., 1980 [3]  Mahlaoui et al., 2013 [1] | Mahlaoui et al., 2013 [1]  Perry et al., 1980 [3] | Albert et al., 2010 [1] |
|  | Multiple publications from a single center with overlapping enrollment periods | The publication documenting the largest number of fatal hemorrhagic events was prioritized for inclusion | Chen et al., 2015 [1]  Luo et al., 2021 [2] | Luo et al., 2021 [2] | Chen et al., 2015 [1] |
| Anatomical site distribution of fatal hemorrhage  (intracranial hemorrhage) | A global multicenter study had overlapping enrollment periods with several single-country studies conducted at its collaborating centers | The global multicenter study was prioritized for exclusion* | Albert et al., 2010 [3]  Perry et al., 1980 [31]  Imai et al., 2004 [5]  Mahlaoui et al., 2013 [3] | Perry et al., 1980 [3]  Imai et al., 2004 [5]  Mahlaoui et al., 2013 [3] | Albert et al., 2010 [3] |
|  | A multicenter study had overlapping enrollment periods with a single-center study from its collaborating centers | The multicenter study was prioritized for inclusion | Lum et al., 1980 [1]  Perry et al., 1980 [31] | Perry et al., 1980 [31] | Lum et al., 1980 [1] |
|  | Multiple publications from a single center with overlapping enrollment periods | The publication documenting the largest number of fatal hemorrhagic events was prioritized for inclusion | Chen et al., 2015 [1]  Luo et al., 2021 [10] | Luo et al., 2021 [10] | Chen et al., 2015 [1] |
| Anatomical site distribution of fatal hemorrhage  (gastrointestinal bleeding) | A global multicenter study had overlapping enrollment periods with a single-country study conducted at its collaborating centers | The global multicenter study was prioritized for exclusion* | Albert et al., 2010 [1]  Perry et al., 1980 [4] | Perry et al., 1980 [4] | Albert et al., 2010 [1] |

Note: Parentheses () indicate the total sample size for the corresponding analysis; brackets [] indicate the number of fatal hemorrhagic events at the respective site.

*The global multicenter study was excluded in this setting to avoid under-ascertainment of individual fatal cases.

# Supplementary Table 5 Participating Institutions/Collaborating Centers of included studies.

| Study,  publication year | Country | Enrollment period | Participating sites/Collaborating centers |
| --- | --- | --- | --- |
| Somerville et al., 1993 (1) | Australia | 1960-1990 | Department of Immunology, Princess Margaret Hospital for Children (Perth) |
| Santos et al.,  2025 (2) | Brazil | NA | Hematology Center (Hemocentro UNICAMP), University of Campinas; Brazilian Group for Immunodeficiency (BRAGID); Latin American Society for Immunodeficiencies (LASID) (**Nationwide**) |
| Chen et al.,  2015 (3) | China | 2004-2014 | Children’s Hospital of Chongqing Medical University (Chongqing) |
| Huang et al.,  2023 (4) | China | 2016-2022 | Xi'an Children's Hospital Affiliated to Xi'an Jiaotong University (Xi'an, Shanxi) |
| Jiang et al.,  2011 (5) | China | 2006-2010 | Jiangsu Institute of Hematology, the First Affiliated Hospital of Soochow University (Suzhou, Jiangsu) |
| Jiang et al.,  2022 (6) | China | 2018-2020 | Department of Pediatrics of the First Affiliated Hospital of Guangxi Medical University (Nanning, Guangxi); Xiamen Children's Hospital Affiliated to Fudan University (Xiamen, Fujian) |
| Jin et al.,  2019 (7) | China | 2004-2016 | Children’s National Medical Center, Shanghai Children’s Medical Center (Shanghai) |
| Lee et al.,  2008 (8) | China | 1980-2006 | Immunodeficiency Diagnosis and Research Institute; Chang Gung Memory Hospital and University College of Medicine (Taoyuan, Taiwan); National Taiwan University Hospital (Taipei, Taiwan) |
| Lee et al.,  2009 (9) | China | 1991-2008 | Department of Pediatrics and Adolescent Medicine, Queen Mary Hospital;  Department of Pediatrics and Adolescent Medicine, the University of Hong Kong (Hong Kong);  Department of Pediatrics, Guangdong Provincial People’s Hospital (Guangzhou, Guangdong). |
| Lee et al.,  2010 (10) | China | 1993-2009 | Chang Gung Memorial & Children's Hospital and University College of Medicine (Taoyuan, Taiwan);  China Medical University (Taichung, Taiwan); National Taiwan University Hospital (Taipei, Taiwan) |
| Li et al.,  2015 (11) | China | 2000-2015 | Children’s Hospital of Chongqing Medical University (Chongqing) |
| Luo et al.,  2021 (12) | China | 2007-2020 | Children’s Hospital of Chongqing Medical University (Chongqing) |
| Luo et al.,  2023 (13) | China | 2018-2021 | Xinhua Hospital, Affiliated to Shanghai Jiao Tong University School of Medicine (Shanghai) |
| Wang et al.,  2020 (14) | China | 2015-2019 | Beijing Children's Hospital, Capital Medical University (Beijing) |
| Zheng et al.,  2019 (15) | China | 2013-2018 | Department of Hematology, the Affiliated Children’s Hospital of Soochow University (Suzhou, Jiangsu) |
| Zhou et al.,  2023 (16) | China | 2006-2020 | Department of Hematology/Oncology, Shanghai Children's Medical Center, Shanghai Jiao Tong University School of Medicine (Shanghai) |
| Sun et al.,  2024 (17) | China | NA | Affiliated Hospital of Qingdao University (Qingdao, Shandong) |
| Mahlaoui et al.,  2013 (18) | France | 1983-2010 | The French National Center for Primary Immunodeficiencies (CEREDIH), a national network of 58 medical departments (**Nationwide**) |
| Suri et al.,  2021 (19) | India | NA | Postgraduate Institute of Medical Education and Research (PGIMER) (Chandigarh); Apollo Hospitals (Chennai); Bai Jerbai Wadia Hospital for Children (Mumbai); Aster CMI Hospital (Bengaluru); Kasturba Medical College, Manipal Academy of Higher Education; King George’s Medical University (Lucknow). |
| David et al.,  2012 (20) | India | NA | Department of Hematology, Christian Medical College (Vellore) |
| Esmaeilzadeh et al.,  2025 (21) | Iran | 2014-2024 | Allergy Research Center, Shiraz University of Medical Sciences (Shiraz); Department of Allergy and Clinical Immunology, Namazi Hospital, Shiraz University of Medical Sciences (Shiraz); Research Center for Immunodeficiencies, Pediatrics Center of Excellence, Children's Medical Center, Tehran University of Medical Sciences (Tehran); Department of Molecular and Cellular Biology, Azad University of Mashhad (Mashhad) |
| Palevski et al.,  2023 (22) | Israel | NA | Sheba Medical Center (Tel HaShomer) |
| Soresina et al.,  2025 (23) | Italy | 2004-2018 | The Italian Association of Pediatric Hematology and Oncology (AIEOP)-Italian Network for Primary Immunodeficiencies (IPINet) (**Nationwide**) |
| Imai et al.,  2004 (24) | Japan | NA | Tokyo Medical and Dental University; National Defense Medical College (Saitama) |
| Albert et al.,  2010 (25) | Multiple countries | NA | **Austria**: Krankenhaus St. Josef (Braunau) **France**: Centre Hospitalier Universitaire Angers (Angers); Hôpital Necker Enfants Malades (Paris) **Germany**: Dr von Haunersches Kinderspital, Ludwig-Maximilians-Universität (Munich); Charité Campus Virchow-Klinikum, Otto-Heubner-Zentrum für Kinder- und Jugendmedizin (Berlin); Universitätsklinikum Freiburg (Freiburg); Universitätsklinik für Kinder- und Jugendmedizin Ulm (Ulm); University Children’s Hospital, Technische Universität (Munich); Frauenklinik am Klinikum rechts der Isar, Technische Universität (Munich); Lehrstuhl für Genomorientierte Bioinformatik, Wissenschaftszentrum Weihenstephan, Technische Universität (Freising)  **Italy**: University of Brescia (Brescia)  **Japan**: National Defense Medical College (Tokorozawa); Tokyo Medical and Dental University (Tokyo) **The Netherlands**: St Radboud University Nijmegen Medical Centre (Nijmegen) **Spain**: Vall d’Hebron Hospital (Barcelona); Centre de Régulació Genòmica, Centro de Investigación Biomédica en Red de Enfermedades Raras (Barcelona)  **Sweden**: The Queen Silvia Children’s Hospital (Göteborg)  **UK**: University College London Institute of Child Health (London)  **USA**: University of Washington, Seattle Children’s Hospital (Seattle) |
| Khoreva et al., 2021 (26) | Russia | 2012-2019 | Dmitry Rogachev National Medical Research Center of Pediatric Hematology, Oncology and Immunology (Moscow) |
| Harfi et al., 1992 (27) | Saudi Arabia | 1981-1990 | King Faisal Specialist Hospital and Research Centre (Riyadh) |
| Lee et al., 2013 (28) | South Korea | NA | The Catholic University of Korea College of Medicine (Seoul) |
| [Udomkittivorakul et al., 2022](https://pubmed.ncbi.nlm.nih.gov/?size=100&term=Udomkittivorakul+N&cauthor_id=34705590) (29) | Thailand | 2008-2021 | Department of Pediatrics, Faculty of Medicine Ramathibodhi Hospital, Mahidol University (Bangkok) |
| Radl et al., 1976 (30) | The Netherlands | NA | Department of Pediatrics, University of Leiden (Leiden) |
| Gök et al., 2025 (31) | Turkey | NA | Department of Pediatrics, School of Medicine, Erciyes University (Kayseri); Kayseri City Hospital (Kayseri); Atatürk University (Erzurum); Gaziantep City Hospital (Gaziantep); and Medical Point Hospital (Gaziantep) |
| Bildik et al., 2022 (32) | Turkey | 1989-2014 | Division of Pediatric Immunology, Hacettepe University Ihsan Dogramaci Children’s Hospital (Ankara) |
| Haskologlu et al., 2020 (33) | Turkey | 1982-2019 | Ankara University Medical School’s Department of Pediatric Immunology and Allergy (Ankara) |
| Faganello et al., 2008 (34) | UK | NA | Bristol Heart Institute, Bristol Royal Infirmary (Bristol) |
| Sullivan et al., 1994 (35) | USA | NA | Johns Hopkins University School of Medicine (Baltimore, Maryland); Children's Hospital of Philadelphia (Philadelphia, Pennsylvania); National Cancer Institute, National Institutes of Health (Bethesda, Maryland) |
| Mathew et al., 1995 (36) | USA | 1990-1994 | St. Jude Children's Research Hospital (Memphis) |
| Lum et al., 1980 (37) | USA | NA | National Institutes of Health (Bethesda) |
| Shin et al., 2012 (38) | USA | 1990-2009 | Cincinnati Children’s Hospital Medical Center (CCHMC) (Cincinnati) |
| Perry et al., 1980 (39) | USA and Canada | 1892-1979 | University of Minnesota Hospital (Minneapolis)  Patients from 42 states in the United States, five provinces in Canada. |
| Burroughs et al, 2020 (40) | USA and Canada | 2005-2015 | 29 US and Canadian centers of the Primary Immune Deficiency Treatment Consortium (PIDTC) |

# Supplementary Table 6 Quality assessment of included cohort studies.

| No. | Author,  publication year | Questions assessing the included studies | | | | | | | | | | | Total  Yes% | Overall  quality |
| --- | --- | --- | --- | --- | --- | --- | --- | --- | --- | --- | --- | --- | --- | --- |
|  |  | **Q1** | **Q2** | **Q3** | **Q4** | **Q5** | **Q6** | **Q7** | **Q8** | **Q9** | **Q10** | **Q11** |  |  |
| 1 | Albert, 2010 | Yes | Yes | Yes | Yes | Yes | Yes | Yes | Yes | Yes | NA | Yes | 100% | High |
| 2 | Bildik, 2022 | Yes | Yes | Yes | Yes | Yes | Yes | Yes | Yes | Unclear | Yes | Yes | 91% | High |
| 3 | Burroughs, 2020 | NA | NA | Yes | Yes | Yes | Yes | Yes | Yes | Yes | NA | Yes | 100% | High |
| 4 | Chen, 2015 | Yes | Yes | Yes | Yes | Yes | Unclear | Yes | Unclear | Yes | NA | Yes | 80% | High |
| 5 | Esmaeilzadeh, 2025 | NA | NA | Yes | Yes | Unclear | Yes | Yes | Yes | No | No | Yes | 67% | Moderate |
| 6 | Gök, 2025 | NA | NA | Yes | Yes | Unclear | Yes | Yes | Yes | Unclear | No | Yes | 67% | Moderate |
| 7 | Haskoloğlu, 2020 | Yes | Yes | Yes | Yes | Yes | Yes | Yes | Yes | Yes | NA | Yes | 100% | High |
| 8 | Imai, 2004 | Yes | Yes | Yes | Yes | Yes | Yes | Yes | Yes | Yes | NA | Yes | 100% | High |
| 9 | Jin, 2019 | Yes | Yes | Yes | Yes | Yes | Yes | Yes | Unclear | No | Yes | Yes | 82% | High |
| 10 | Khoreva, 2021 | NA | NA | Yes | Yes | Yes | Yes | Yes | Yes | Unclear | No | Yes | 78% | Moderate |
| 11 | Lee, 2008 | Yes | Yes | Yes | Yes | Yes | Yes | Yes | Unclear | Yes | NA | Yes | 90% | High |
| 12 | Lee, 2009 | Yes | Yes | Yes | Yes | Yes | Yes | Yes | Yes | Yes | NA | Yes | 100% | High |
| 13 | Lee, 2010 | Yes | Yes | Yes | Yes | Yes | Yes | Yes | Yes | Yes | NA | Yes | 100% | High |
| 14 | Li, 2015 | NA | NA | Yes | Yes | Yes | Yes | Yes | Yes | No | Yes | Yes | 89% | High |
| 15 | Perry, 1980 | NA | NA | Unclear | Yes | Yes | Unclear | Yes | Yes | No | Yes | Yes | 67% | Moderate |
| 16 | Santos, 2025 | Yes | Yes | Yes | Yes | No | Yes | Yes | Unclear | Unclear | No | Yes | 64% | Moderate |
| 17 | Shin, 2012 | NA | NA | Yes | Yes | Yes | Yes | Yes | Yes | Yes | NA | Yes | 100% | High |
| 18 | Soresina, 2025 | Yes | Yes | Yes | Yes | Yes | Yes | Yes | Yes | Yes | NA | Yes | 100% | High |
| 19 | Sullivan, 1994 | NA | NA | Unclear | Yes | Yes | Unclear | Yes | Yes | No | Yes | Yes | 67% | Moderate |
| 20 | Suri, 2021 | Yes | Yes | Yes | Yes | Yes | Yes | Unclear | Yes | Unclear | Yes | Yes | 82% | High |
| 21 | Wang, 2020 | Yes | Yes | Yes | Yes | Yes | Yes | Yes | Unclear | No | No | Yes | 73% | Moderate |
| 22 | Zheng, 2019 | Yes | Yes | Yes | Unclear | No | Yes | Yes | Yes | Yes | NA | Yes | 80% | High |
| 23 | Zhou, 2023 | NA | NA | Yes | Yes | Yes | Yes | Yes | Unclear | Yes | NA | Yes | 88% | High |
| 24 | Mahlaoui, 2013 | Yes | Yes | Yes | Yes | Unclear | Yes | Yes | Yes | Yes | NA | Yes | 90% | High |

Q1: Were the two groups similar and recruited from the same population?

Q2: Were the exposures measured similarly to assign people to both exposed and unexposed groups?

Q3: Was the exposure measured in a valid and reliable way?

Q4: Were confounding factors identified?

Q5: Were strategies to deal with confounding factors stated?

Q6: Were the groups/participants free of the outcome at the start of the study (or at the moment of exposure)?

Q7: Were the outcomes measured in a valid and reliable way?

Q8: Was the follow up time reported and sufficient to be long enough for outcomes to occur?

Q9: Was follow up complete, and if not, were the reasons to loss to follow up described and explored?

Q10: Were strategies to address incomplete follow up utilized?

Q11: Was appropriate statistical analysis used?

NA, Not applicable

# Supplementary Table 7 Quality assessment of included case series.

| No. | Author,  publication year | Questions assessing the included studies | | | | | | | | | | Total  Yes% | Overall  quality |
| --- | --- | --- | --- | --- | --- | --- | --- | --- | --- | --- | --- | --- | --- |
|  |  | **Q1** | **Q2** | **Q3** | **Q4** | **Q5** | **Q6** | **Q7** | **Q8** | **Q9** | **Q10** |  |  |
| 1 | Lum, 1980 | Yes | Yes | Yes | Unclear | Yes | Yes | Yes | Yes | Unclear | Yes | 80% | High |
| 2 | Luo, 2023 | Yes | Yes | Yes | Unclear | Yes | Yes | Yes | Yes | Unclear | Yes | 80% | High |
| 3 | Huang, 2023 | Yes | Yes | Yes | Unclear | Unclear | Yes | Yes | Yes | Yes | Yes | 80% | High |
| 4 | Jiang, 2022 | Yes | Yes | Yes | Unclear | Unclear | Yes | Yes | Yes | Unclear | Yes | 70% | Moderate |
| 5 | David, 2012 | Yes | Yes | Yes | Unclear | Unclear | Yes | Yes | Yes | Unclear | Yes | 70% | Moderate |
| 6 | Jiang, 2011 | Yes | Yes | Yes | Unclear | Unclear | Yes | Yes | No | Yes | Yes | 70% | Moderate |
| 7 | Palevski, 2023 | No | Yes | Yes | Unclear | Unclear | Yes | Yes | Yes | Unclear | Yes | 60% | Moderate |
| 8 | Udomkittivorakul, 2022 | Yes | Yes | Yes | Unclear | Unclear | Yes | Yes | Yes | Unclear | Yes | 70% | Moderate |
| 9 | Mathew, 1995 | Yes | Yes | Yes | No | No | Yes | Yes | Yes | Unclear | Yes | 70% | Moderate |
| 10 | Harfi, 1992 | Yes | Yes | Yes | Unclear | Unclear | Yes | Yes | Yes | Unclear | Yes | 70% | Moderate |
| 11 | Radl, 1976 | Unclear | Yes | Yes | Unclear | Unclear | Yes | Yes | Yes | Unclear | Yes | 60% | Moderate |
| 12 | Somerville, 1993 | Yes | Yes | Yes | Yes | Yes | Yes | Yes | Yes | Yes | Yes | 100% | High |

Q1: Were there clear criteria for inclusion in the case series?

Q2: Was the condition measured in a standard, reliable way for all participants included in the case series?

Q3: Were valid methods used for identification of the condition for all participants included in the case series?

Q4: Did the case series have consecutive inclusion of participants?

Q5: Did the case series have complete inclusion of participants?

Q6: Was there clear reporting of the demographics of the participants in the study?

Q7: Was there clear reporting of clinical information of the participants?

Q8: Were the outcomes or follow up results of cases clearly reported?

Q9: Was there clear reporting of the presenting site(s)/clinic(s) demographic information?

Q10: Was statistical analysis appropriate?

# Supplementary Table 8 Quality assessment of included case reports.

| No. | Author,  publication year | Questions assessing the included studies | | | | | | | | Total  Yes% | Overall  quality |
| --- | --- | --- | --- | --- | --- | --- | --- | --- | --- | --- | --- |
|  |  | **Q1** | **Q2** | **Q3** | **Q4** | **Q5** | **Q6** | **Q7** | **Q8** |  |  |
| 1 | Sun, 2024 | Yes | Yes | Yes | Yes | Yes | Yes | Yes | Yes | 100% | High |
| 2 | Lee, 2013 | Yes | Yes | Yes | Yes | Yes | Yes | Yes | Yes | 100% | High |
| 3 | Faganello, 2008 | Unclear | Yes | Yes | Yes | Yes | Yes | Yes | Unclear | 75% | Moderate |

Q1: Were patient’s demographic characteristics clearly described?

Q2: Was the patient’s history clearly described and presented as a timeline?

Q3: Was the current clinical condition of the patient on presentation clearly described?

Q4: Were diagnostic tests or methods and the results clearly described?

Q5: Was the intervention(s) or treatment procedure(s) clearly described?

Q6: Was the post-intervention clinical condition clearly described?

Q7: Were adverse events (harms) or unanticipated events identified and described?

Q8: Does the case report provide takeaway lessons?

# Supplementary Table 9 Quality assessment of included case-control study.

| No. | Author,  publication year | Questions assessing the included studies | | | | | | | | | | Total  Yes% | Overall  quality |
| --- | --- | --- | --- | --- | --- | --- | --- | --- | --- | --- | --- | --- | --- |
|  |  | **Q1** | **Q2** | **Q3** | **Q4** | **Q5** | **Q6** | **Q7** | **Q8** | **Q9** | **Q10** |  |  |
| 1 | Luo, 2021 | Yes | Unclear | Yes | Yes | Yes | Yes | Yes | Yes | Yes | Yes | 90% | High |

1: Were the groups comparable other than the presence of disease in cases or the absence of disease in controls?

2: Were cases and controls matched appropriately?

3: Were the same criteria used for identification of cases and controls?

4: Was exposure measured in a standard, valid and reliable way?

5: Was exposure measured in the same way for cases and controls?

6: Were confounding factors identified?

7: Were strategies to deal with confounding factors stated?

8: Were outcomes assessed in a standard, valid and reliable way for cases and controls?

9: Was the exposure period of interest long enough to be meaningful?

10: Was appropriate statistical analysis used?

# Supplementary Table 10 Meta-regression results for heterogeneity sources of cumulative incidence of severe bleeding.

| Moderator variable | $\boldsymbol{\beta}$coefficient (95% CI) | $\boldsymbol{Q}_{\boldsymbol{M}}$ statistic | $\boldsymbol{p}$ value | Residual heterogeneity after adjustment |
| --- | --- | --- | --- | --- |
| Overall disease severity | 1.69 (0.06-3.32) | 4.12 | 0.0423 | $I^{2}$= 70.69%, $\tau^{2}$= 0.34, $p$< 0.0001 |
| Study sample size | -0.011 (-0.018 to -0.003) | 8.20 | 0.0042 | $I^{2}$= 61.99%, $\tau^{2}$= 0.25, $p$= 0.0002 |

CI, confidence interval; Overall disease severity, the proportion of patients with a WAS score of 5; $Q_{M}$, Q statistic for meta-regression; $I^{2}$, percentage of between-study heterogeneity; $\tau^{2}$, between-study variance.

# Supplementary Table 11 Raw data of studies included in the meta-regression analysis of severe bleeding incidence.

| First, publication year | Total sample size ($\boldsymbol{n}$) | No. of patients with severe bleeding history | Cumulative incidence of severe bleeding | Proportion of patients with a WAS score of 5 |
| --- | --- | --- | --- | --- |
| Lee et al., 2010 | 16 | 4 | 0.25 | 0.25 |
| Li et al., 2015 | 132 | 8 | 0.06 | 0.17 |
| Wang et al., 2020 | 23 | 6 | 0.26 | 0.30 |
| Mahlaoui et al., 2013 | 26 | 13 | 0.50 | 1.00 |
| Suri et al., 2021 | 95 | 7 | 0.07 | 0.41 |
| Esmaeilzadeh et al., 2025 | 41 | 10 | 0.24 | 0.56 |
| Albert et al., 2010 | 173 | 24 | 0.14 | 0.00 |
| Khoreva et al., 2021 | 67 | 9 | 0.13 | 0.40 |
| Gök et al., 2025 | 16 | 4 | 0.25 | 0.06 |
| Shin et al., 2012 | 47 | 17 | 0.36 | 0.34 |

# Supplementary Table 12 Original data from 12 included studies for relative risk analysis of fatal hemorrhage by treatment group in WAS.

| First author | Publication year | Curative group | | Non-curative group | |
| --- | --- | --- | --- | --- | --- |
|  |  | **Event¶ (No.)** | **Total (No.)** | **Event¶ (No.)** | **Total (No.)** |
| Santos et al. (2) | 2025 | 0 | 12 | 2 | 10 |
| Chen et al. (3) | 2015 | 0 | 8 | 2 | 45 |
| Huang et al. (4) | 2023 | 0 | 6 | 1 | 5 |
| Jin et al. (7) | 2019 | 0 | 8 | 2 | 34 |
| Lee et al. (10) | 2010 | 0 | 6 | 1 | 10 |
| Wang et al. (14) | 2020 | 0 | 9 | 1 | 14 |
| Zheng et al. (15) | 2019 | 0 | 24 | 1 | 7 |
| Suri et al. (19) | 2021 | **1** | 25 | 6 | 70 |
| Albert et al. (25) | 2010 | 0 | 25 | 5 | 148 |
| Bildik et al. (32) | 2022 | 0 | 9 | 1 | 14 |
| Haskoloğlu et al. (33) | 2020 | 0 | 11 | 2 | 12 |
| Sullivan et al. (35) | 1994 | 0 | 47 | 12 | 107 |

¶Fatal cases attributable to hemorrhage.

WAS, Wiskott-Aldrich syndrome.

# Supplementary Table 13 Corresponding references for each pooled analysis.

| Outcome | Number of included studies | References |
| --- | --- | --- |
| Cumulative incidence of overall bleeding | 18 | Harfi et al. (1992); Huang et al. (2023); Imai et al. (2004); Zheng et al. (2019); Zhou et al. (2023); Suri et al. (2021); Haskologlu et al. (2020); Sullivan et al. (1994); Somerville et al. (1993); Santos et al. (2025); Jiang et al. (2011); Jiang et al. (2022); Li et al. (2015); Wang et al. (2020); David et al. (2012); Udomkittivorakul et al. (2022); Bidik et al. (2022); Gök et al. (2025). |
| Cumulative incidence of cutaneous bleeding | 19 | Huang et al. (2023); Harfi et al. (1992); Li et al. (2015); Zheng et al. (2019); Imai et al. (2004); Soresina et al. (2025); Lee et al. (2009); Zhou et al. (2023); Haskologlu et al. (2020); Sullivan et al. (1994); Somerville et al. (1993); Santo et al. (2025); Jiang et al. (2022); David et al. (2012); Suri et al. (2021); Khoreva et al. (2021); Haskologlu et al. (2020); Bidik et al. (2022); Gök et al. (2025) |
| Cumulative incidence of epistaxis | 16 | Li et al. (2015); Lee et al. (2009); Soresina et al. (2025); Haskologlu et al. (2020); Sullivan et al. (1994); Santo et al. (2025); Jiang et al. (2022); Jin et al. (2019); Lee et al. (2010); David et al. (2012); Suri et al. (2021); Khoreva et al. (2021); Harfi et al. (1992); Udomkittivorakul et al. (2022); Bidik et al. (2022); Gök et al. (2025). |
| Cumulative incidence of gastrointestinal bleeding | 20 | Huang et al. (2023); Li et al. (2015) ; Zheng et al. (2019); Harfi et al. (1992); Lee et al. (2009); Soresina et al. (2025); Haskologlu et al. (2020); Somerville et al. (1993); Santo et al. (2025); Jiang et al. (2022); Jin et al. (2019); Lee et al. (2010); Luo et al. (2023); David et al. (2012); Suri et al. (2021); Imai et al. (2004); Khoreva et al. (2021); Udomkittivorakul et al. (2022); Bidik et al. (2022); Gök et al. (2025). |
| Cumulative incidence of intracranial hemorrhage | 17 | Lee et al. (2009); Soresina et al. (2025); Haskologlu et al. (2020); Sullivan et al. (1994); Somerville et al. (1993); Jin et al. (2019); Lee et al. (2010); Luo et al. (2021); Mahlaoui et al. (2013); David et al. (2012); Suri et al. (2021); Imai et al. (2004); Lee et al. (2010); Khoreva et al (2021); Harfi et al. (1992); Udomkittivorakul et al. (2022); Bidik et al. (2022); Gök et al. (2025). |
| Cumulative incidence of severe bleeding | 10 | Lee et al. (2010); Li et al. (2015); Wang et al. (2020); Mahlaoui et al. (2013); Suri et al. (2021); Esmaeilzadeh et al. (2025); Albert et al. (2010); Khoreva et al (2021); Gök et al. (2025); Shin et al. (2012). |
| Cumulative incidence of multisystem bleeding | 8 | Santos et al. (2025); Jiang et al. (2022); Jin et al. (2019); David et al. (2012); Suri et al. (2021); Harfi et al. (1992); Udomkittivorakul et al. (2022); Gök et al. (2025). |
| Cause-specific mortality rate from hemorrhage in curatively treated patients | 18 | Santos et al. (2025); Chen et al. (2015); Huang et al. (2023); Lee et al. (2009); Lee et al. (2010); Luo et al. (2023); Wang et al. (2020); Zheng (2019); Zhou et al. (2023); Mahlaoui et al. (2013); Suri et al. (2021); Soresina et al. (2025); Imai et al. (2004); Bidik et al. (2022); Gök et al. (2025); Haskologlu et al. (2020); Sullivan et al. (1994); Burroughs et al. (2020). |
| Cause-specific mortality rate from hemorrhage in non-curatively treated patients | 16 | Somerville et al. (1993); Santos et al. (2025); Huang et al. (2023); Jiang et al. (2011); Jin et al. (2019); Lee et al. (2009); Lee et al. (2010); Luo et al. (2021); Wang et al. (2020); Zheng et al. (2019); Suri et al. (2021); Albert et al. (2010); Udomkittivorakul et al. (2022); Bidik et al. (2022); Haskologlu et al. (2020); Sullivan et al. (1994). |
| The pooled relative risk for fatal hemorrhage between the curative and non-curative groups | 12 | Santos et al. (2025); Chen et al. (2015); Huang et al. (2023); Jin et al. (2019); Jin et al. (2019); Wang et al. (2020); Zheng (2019); Suri et al. (2021); Albert et al. (2010); Bidik et al. (2022); Haskologlu et al. (2020); Sullivan et al. (1994). |
| The proportional mortality ratio due to hemorrhage in non-curatively treated patients | 8 | Santos et al. (2025); Jin et al. (2019); Suri et al. (2021); Soresina et al. (2025); Imai et al. (2004); Bidik et al. (2022); Haskologlu et al. (2020); Sullivan et al. (1994). |

# Supplementary Table 14 Summary of studies reporting age at fatal hemorrhagic event.

| Country | Author, year of publication | Age at fatal hemorrhagic event |
| --- | --- | --- |
| Multiple  Countries | Albert et al., 2010 (25) | P1: 2 y (24 m)  P2: 4.9 y (58.8 m)  P3: 74.6 y (985.2 m) |
| Australia | Somerville et al., 1993 (1) | P4: 3 m |
| Brazil | Santos et al., 2025 (2) | P5: 3 m  P6: 3 m |
| China | Chen et al., 2015 (3) | P7: 7.3 m  P8: 5 m |
|  | Huang et al., 2023 (4) | P9: 22 m |
|  | Jin et al., 2019 (7) | P10: 2.5 y (30 m)  P11: 2 m |
|  | Lee et al., 2008 (8) | P12: 25 m |
|  | Lee et al., 2010 (10) | P13: 2 y (24 m) |
|  | Sun et al., 2024 (17) | P14: 12 m |
| France | Mahlaoui et al., 2013(18) | P15: 48 m  P16: 4 m  P17: 15 m  P18: 6 m |
| Israel | Palevski et al., 2023 (22) | P19: 10 m |
| Japan | Imai et al., 2004 (24) | P20: 8 y (96 m) |
| Saudi Arabia | Harfi et al., 1992 (27) | P21: 30 m |
| South Korea | Lee et al., 2013 (28) | P22: 48 m |
| Thailand | Udomkittivorakul et al., 2022 (29) | P23: 12 m  P24: 11 m |
| The Netherlands | Radl et al., 1976 (30) | P25: 48 m |
| Turkey | Gök et al., 2025 (31) | P26: 9 m  P27: 24 m |
|  | Haskoloğlu et al., 2020 (33) | P28: 4 y (48 m)  P29: 4.5 y (54 m) |
| UK | Faganello et al., 2008 (34) | P30: 324 m |
| USA | Lum et al., 1980 (37) | P31: 22 m  P32: 24 m |
|  | Mathew et al., 1995 (36) | P33: 5 m |

# Supplementary Table 15 Anatomical site distribution of fatal hemorrhagic events.

| Site of hemorrhage | Number of studies | Number of patients | Proportion（%） |
| --- | --- | --- | --- |
| Pulmonary hemorrhage  (12,14,18,19,39) | 5 | 8 | 9.09 |
| Intracranial hemorrhage  (1,7,12,15,17–19,22,24,27–29,31,33,39) | 15 | 67 | 76.14 |
| Gastrointestinal bleeding  (12,19,30–32,39) | 6 | 11 | 12.50 |
| Other sites^*^  (34,36) | 2 | 2 | 2.27 |
| Total | 18 | 88 | 100.00 |

***** Retroperitoneal hemorrhage and aortic rupture with hemorrhage.


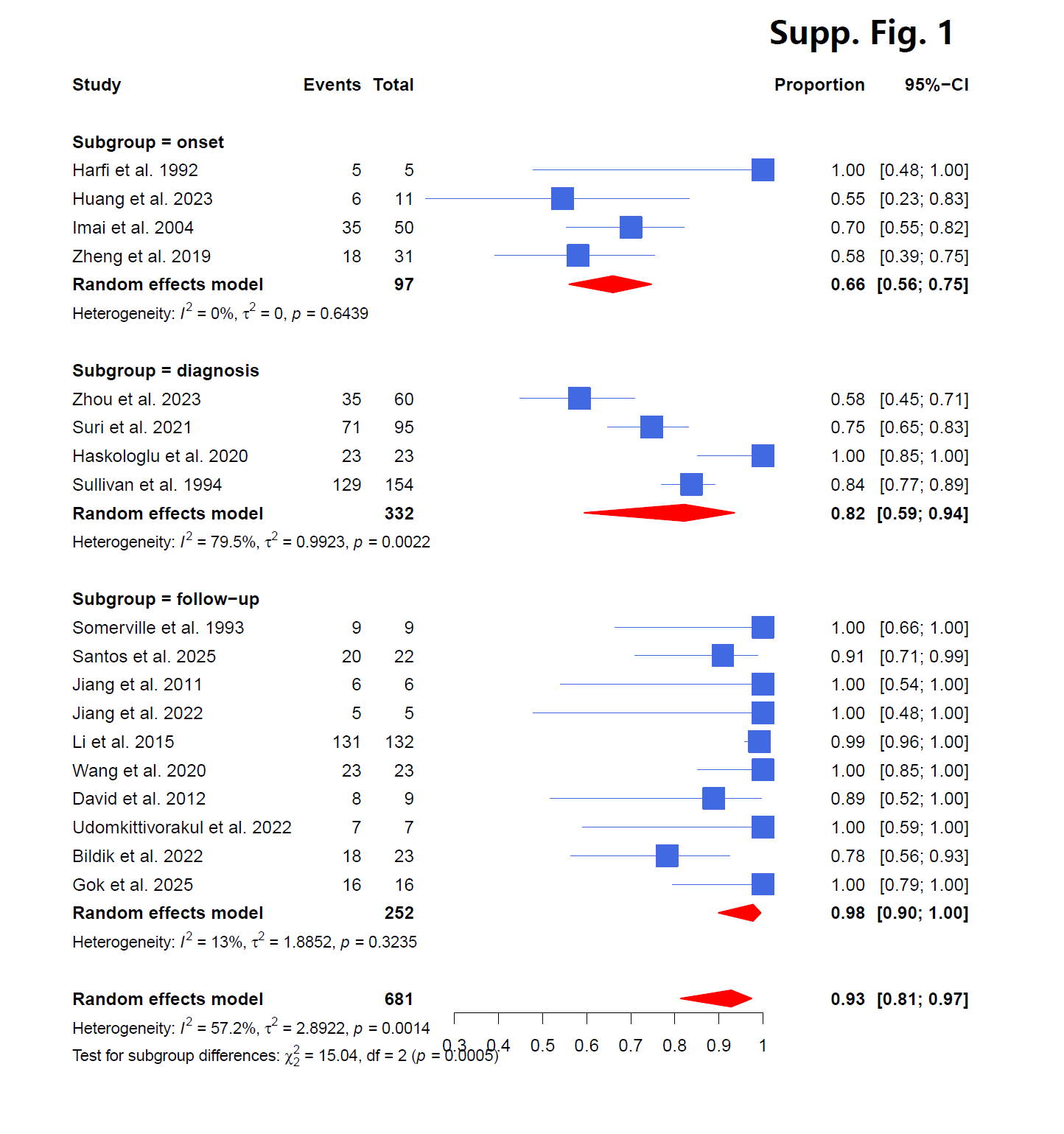


Supplementary Figure 1 Forest plot of the meta-analysis on the cumulative incidence of overall bleeding.


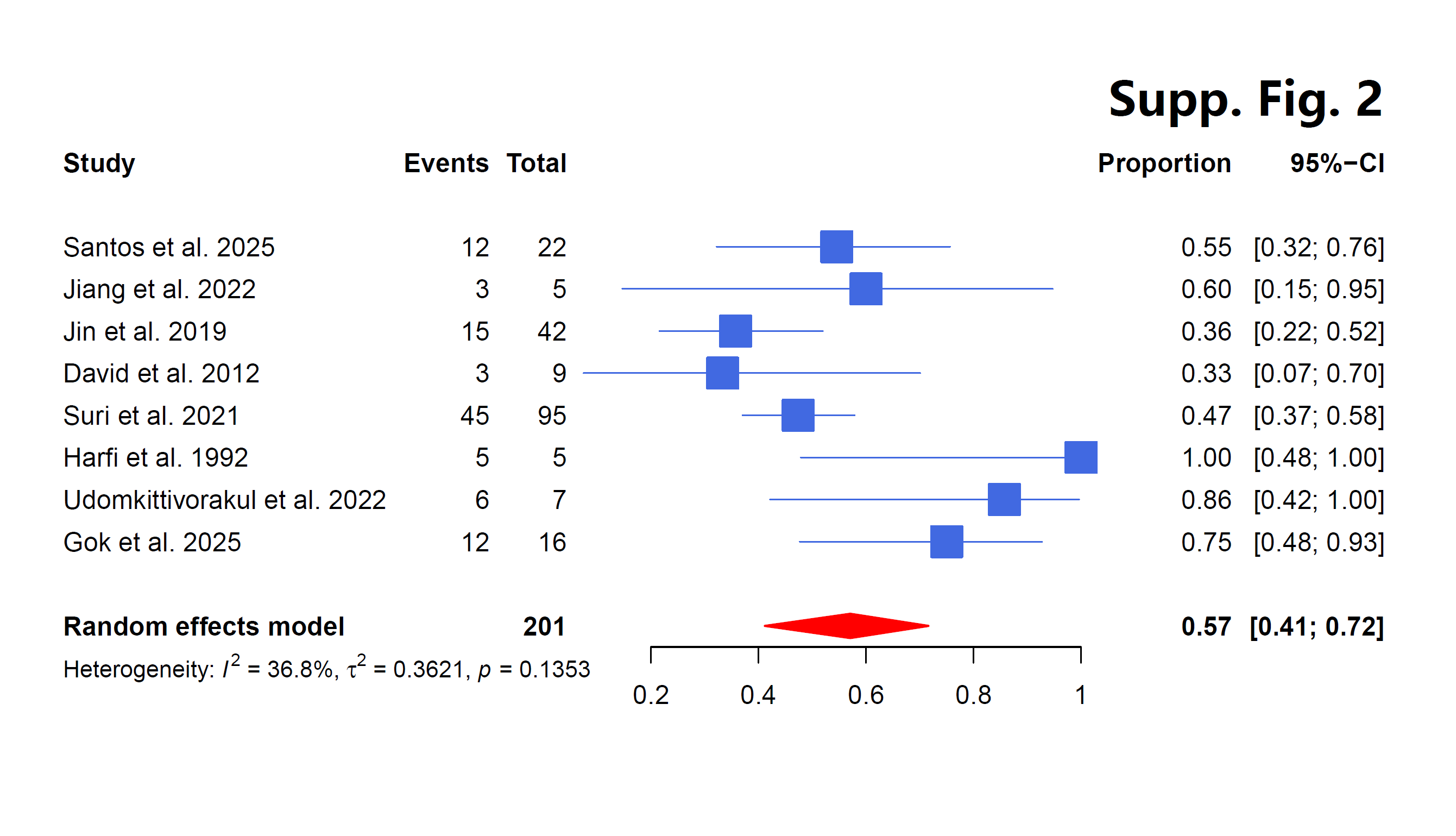


Supplementary Figure 2 Forest plot of the meta-analysis on the cumulative incidence of multisystem bleeding at the end of follow-up.


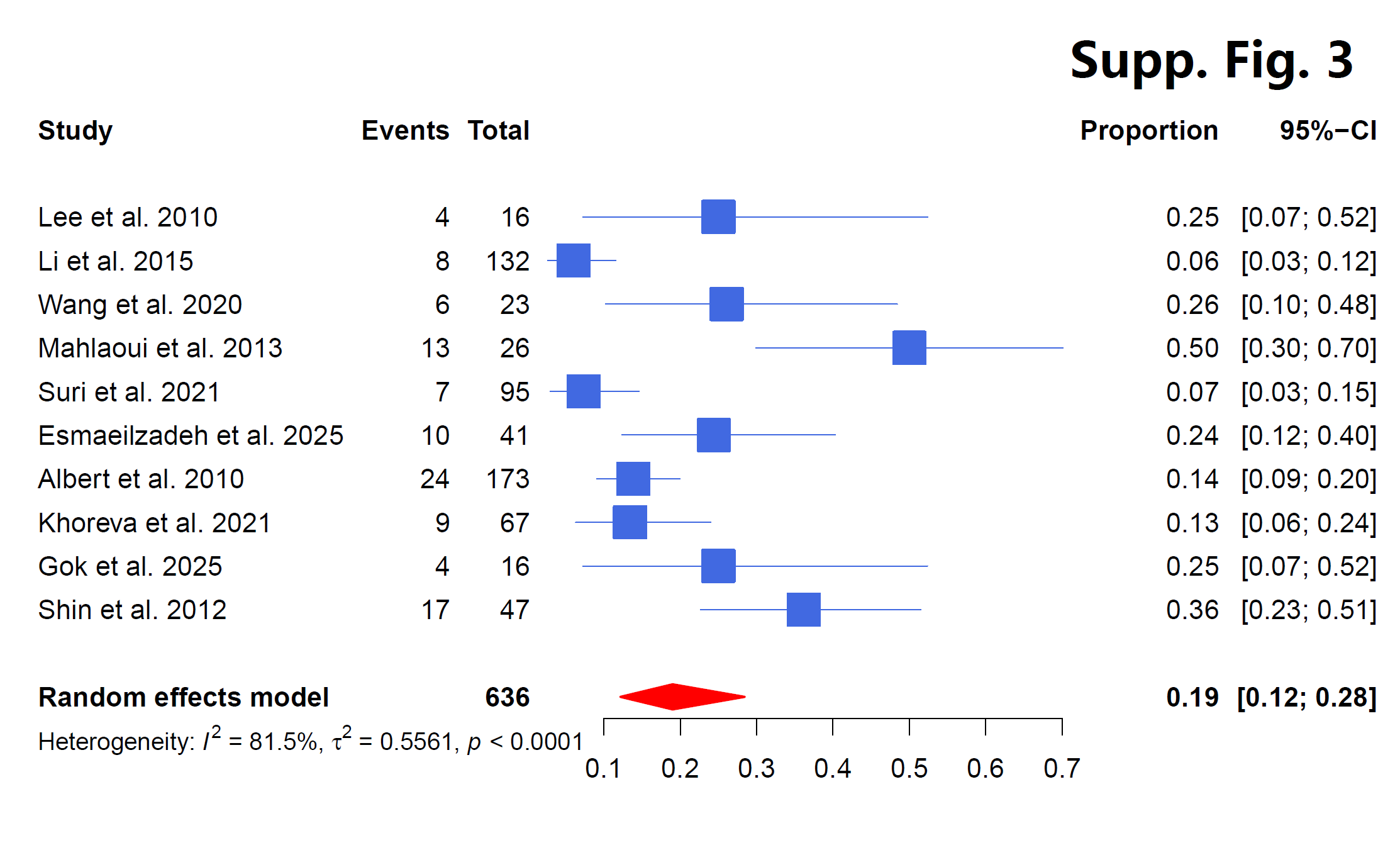


Supplementary Figure 3 Forest plot of meta-analysis on the cumulative incidence of severe bleeding at the end of follow-up.


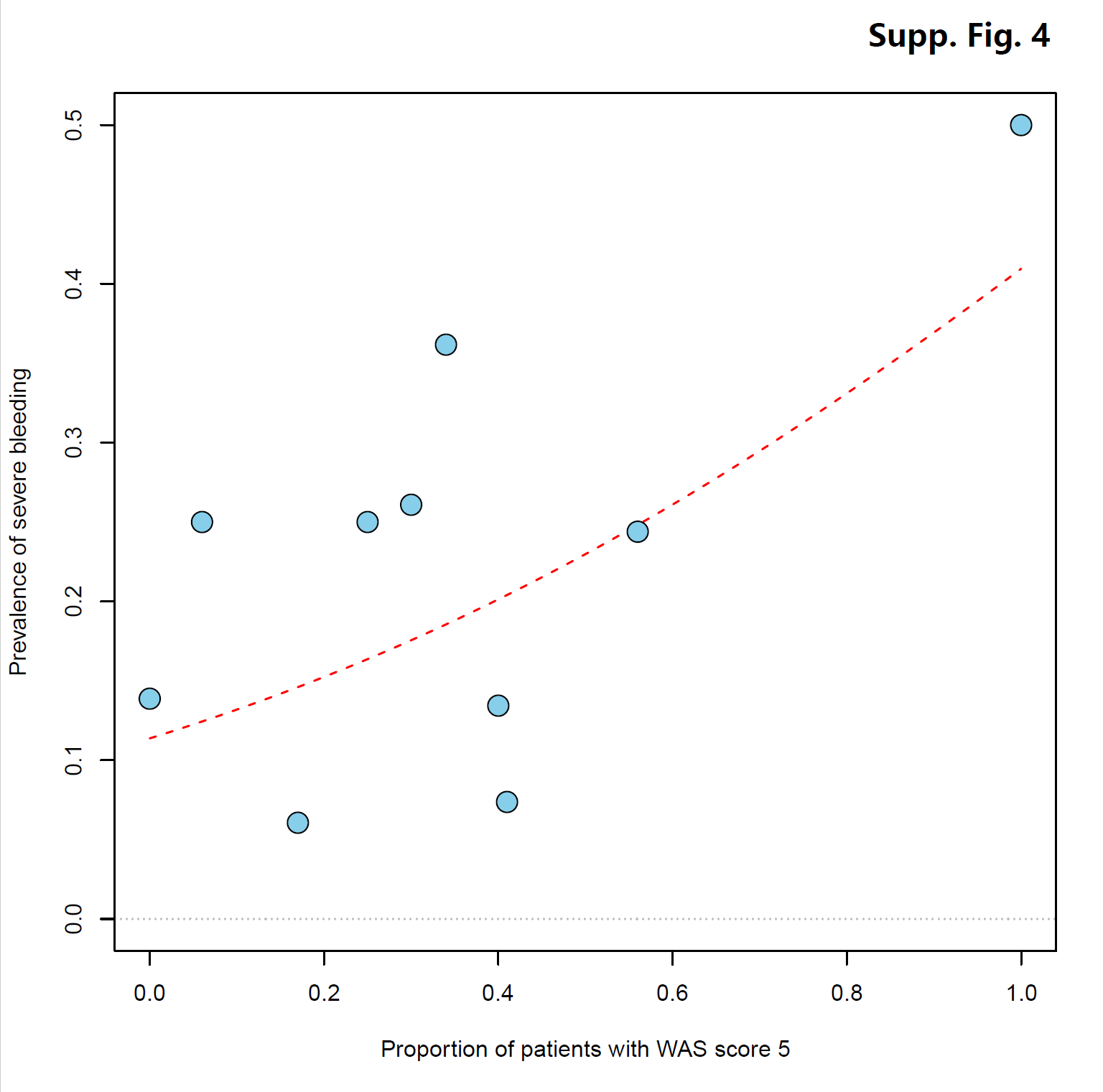


Supplementary Figure 4 Bubble plot showing the association of overall disease severity (the proportion of patients with a WAS score of 5) with the cumulative incidence of severe bleeding at the end of follow-up.


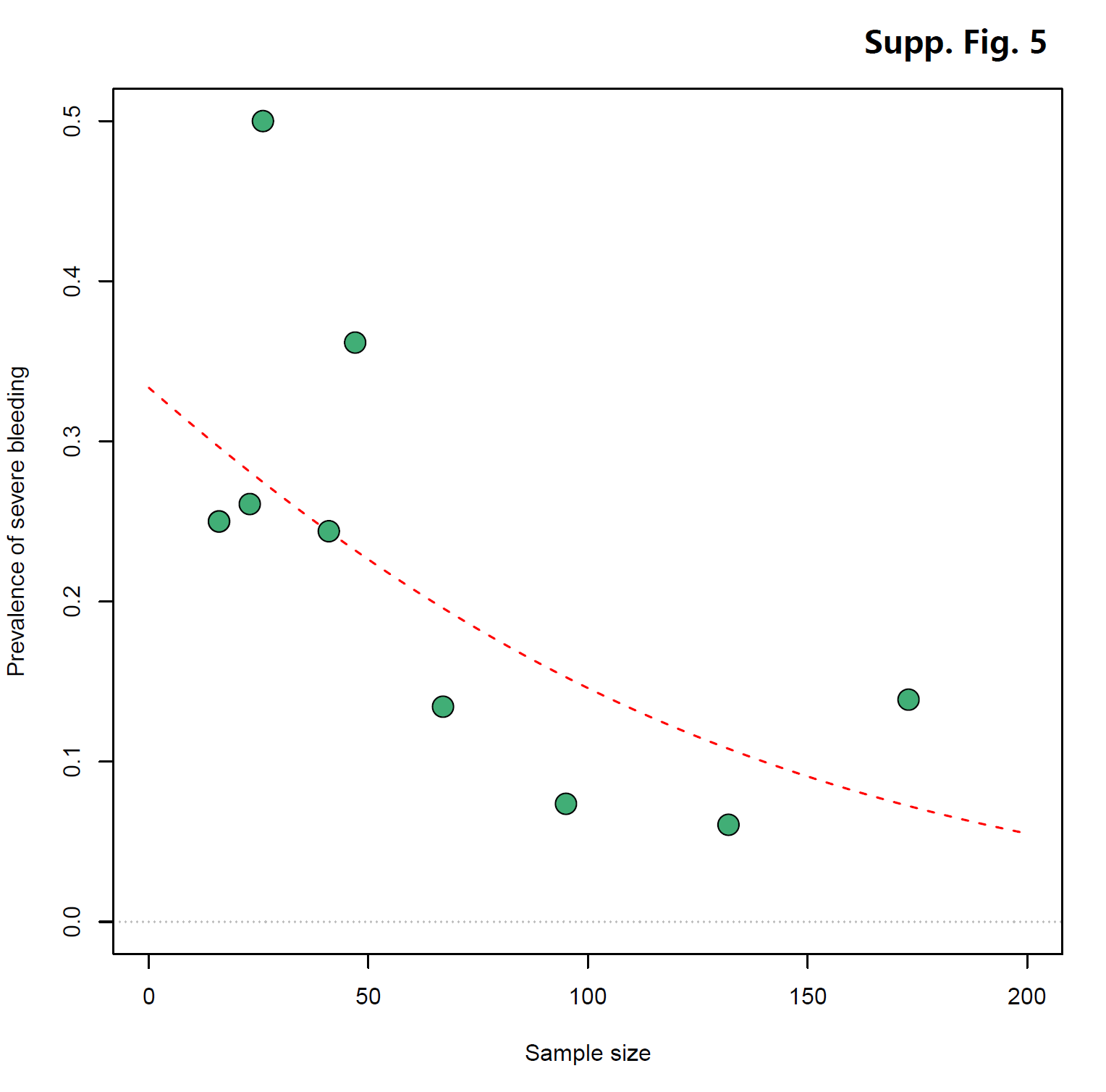


Supplementary Figure 5 Bubble plot showing the association of study sample size with the cumulative incidence of severe bleeding at the end of follow-up.


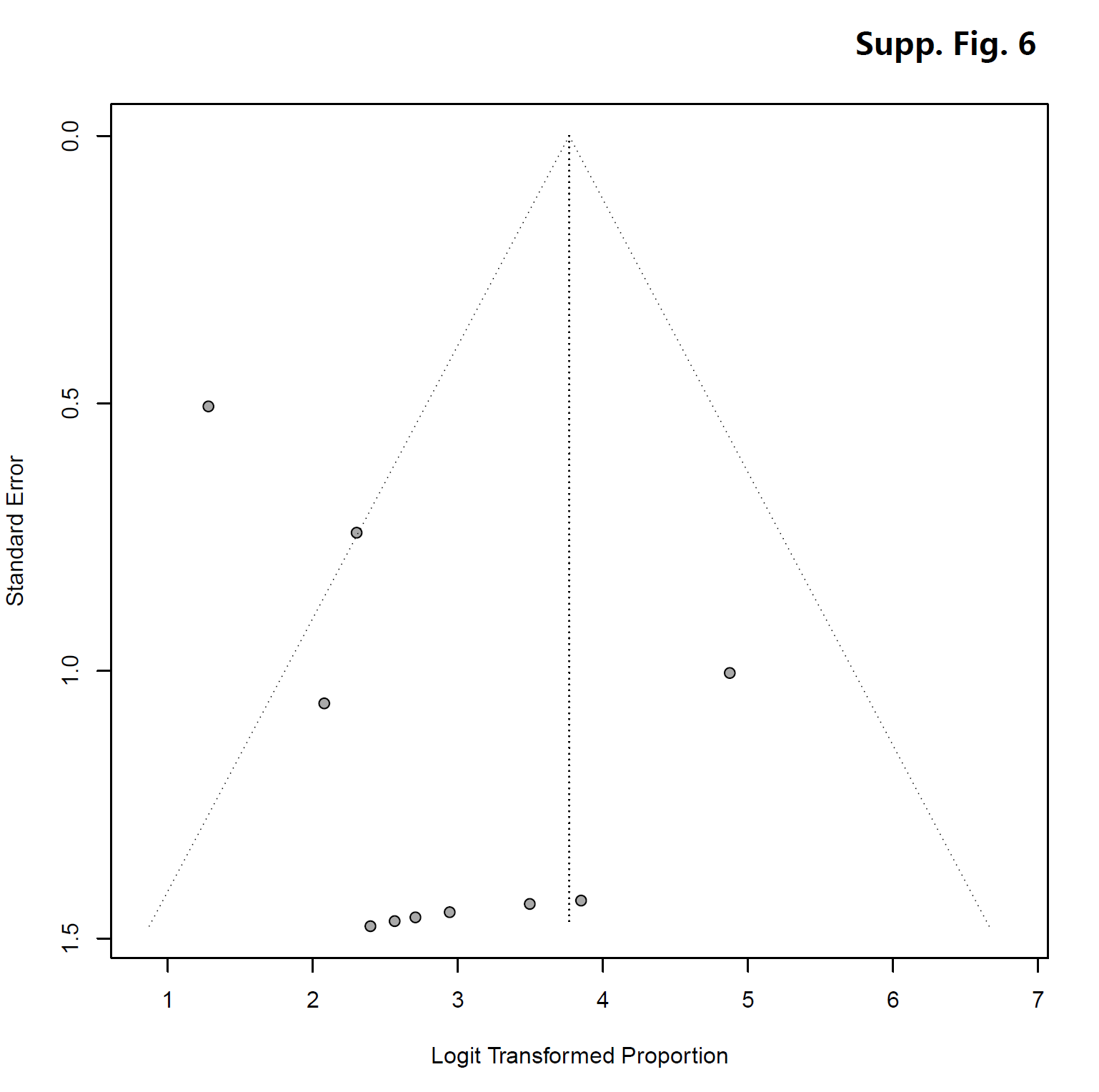


Supplementary Figure 6 Funnel plot of the meta-analysis on the cumulative incidence of overall bleeding at the end of follow-up.


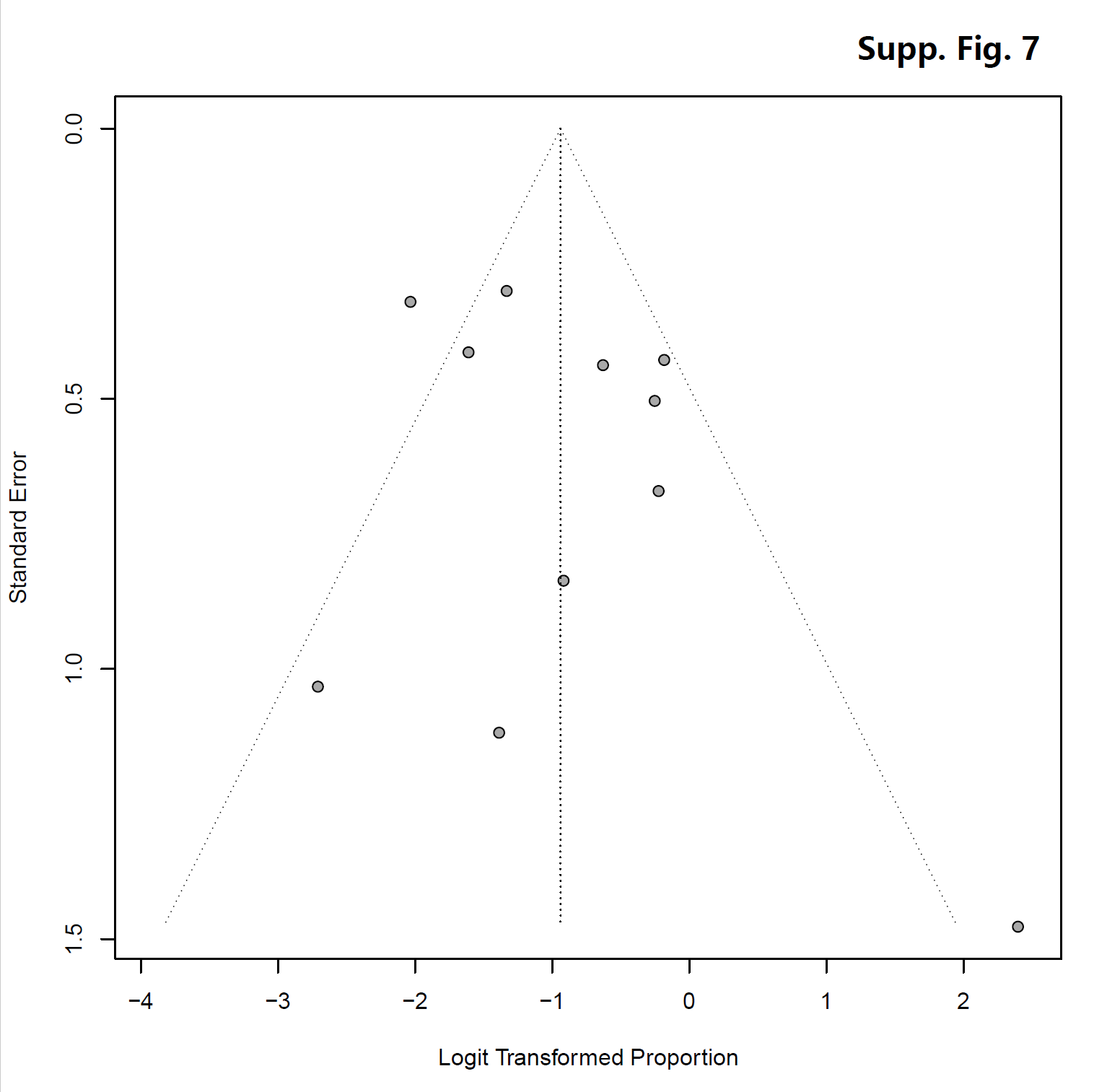


Supplementary Figure 7 Funnel plot of the meta-analysis on the cumulative incidence of epistaxis at the end of follow-up.


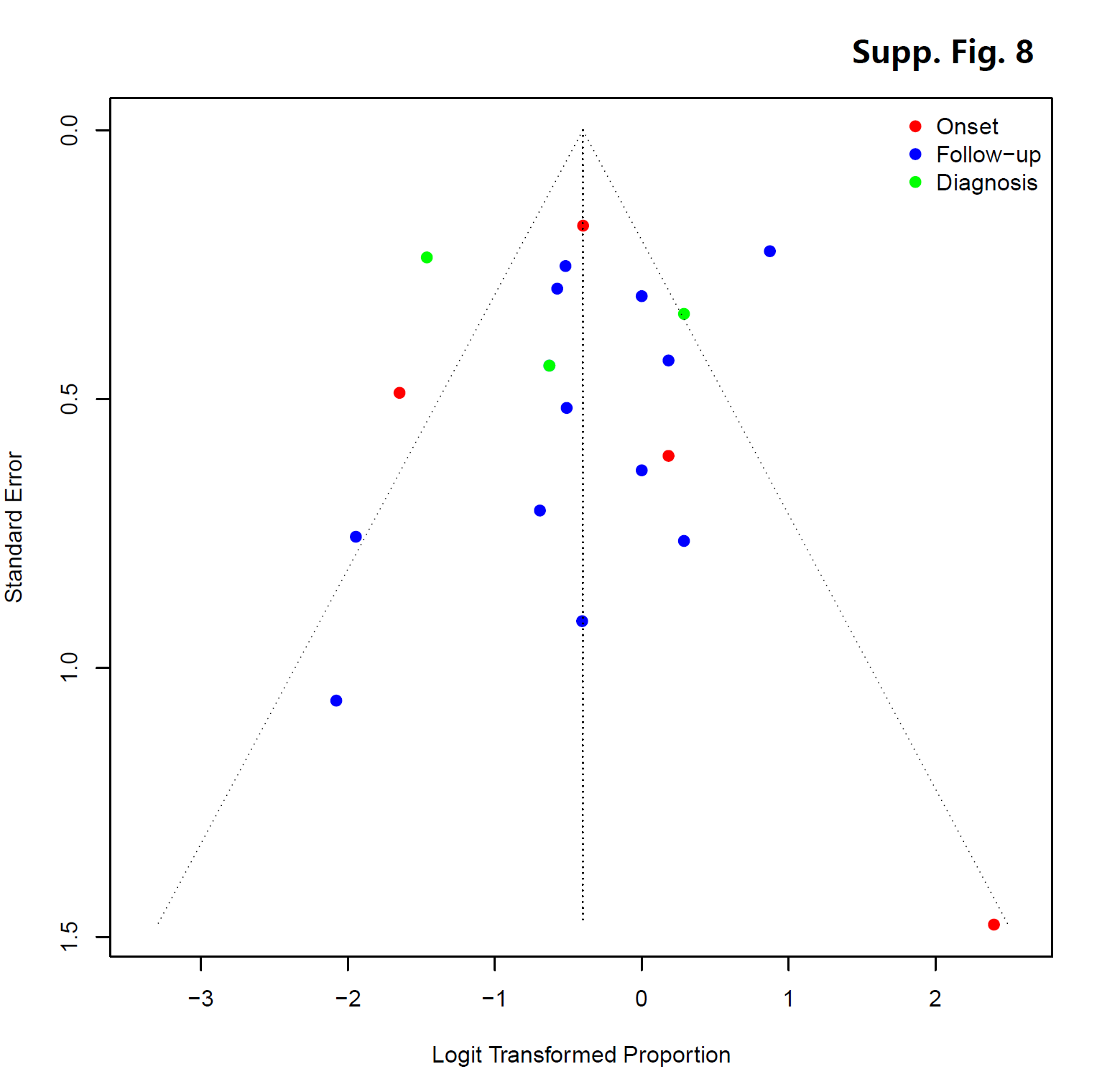


Supplementary Figure 8 Funnel plot of the meta-analysis on the cumulative incidence of gastrointestinal bleeding.


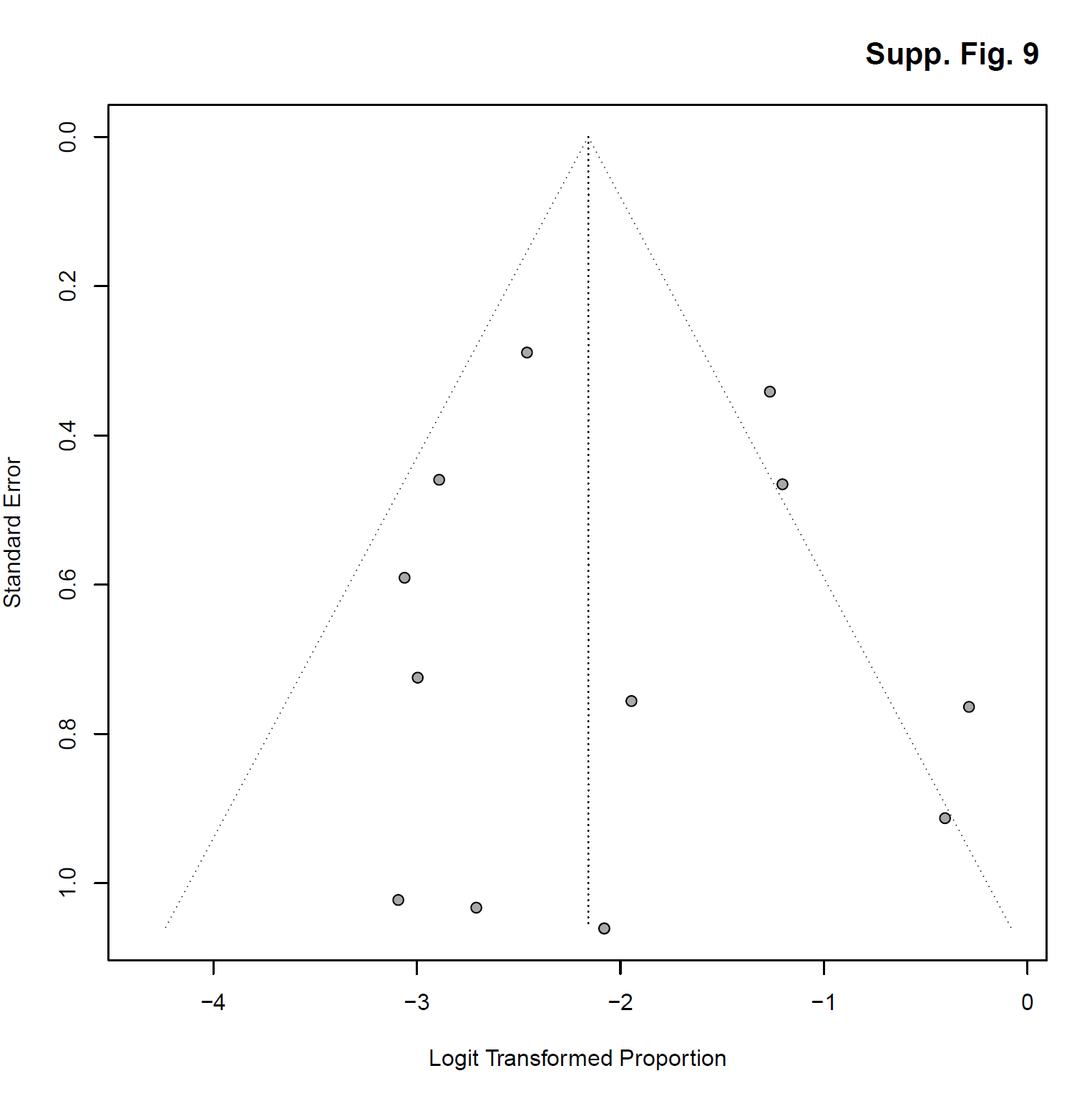


Supplementary Figure 9 Funnel plot of the meta-analysis on the cumulative incidence of intracranial hemorrhage at the end of follow-up.
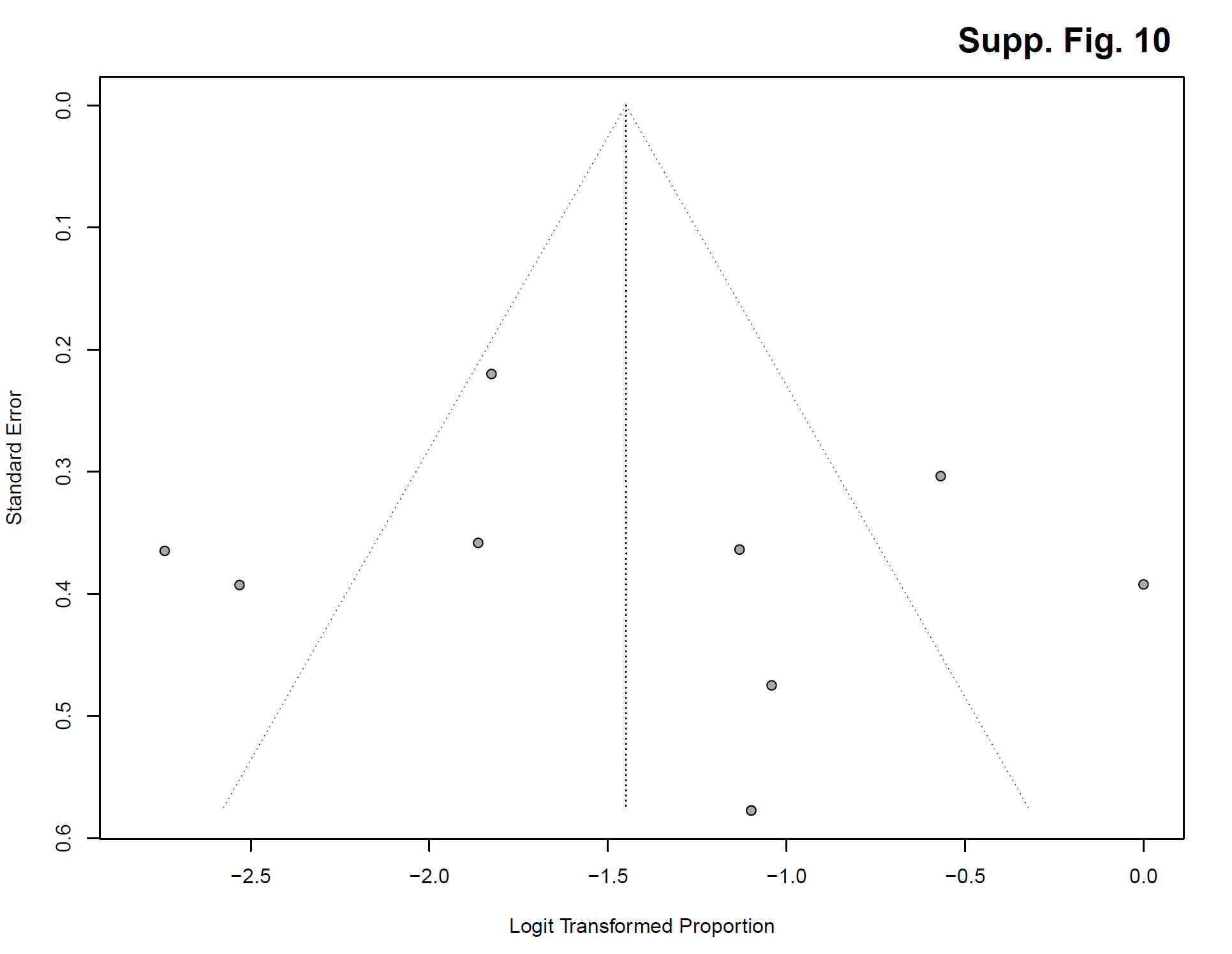


Supplementary Figure 10 Funnel plot of the meta-analysis on the cumulative incidence of severe bleeding at the end of follow-up.
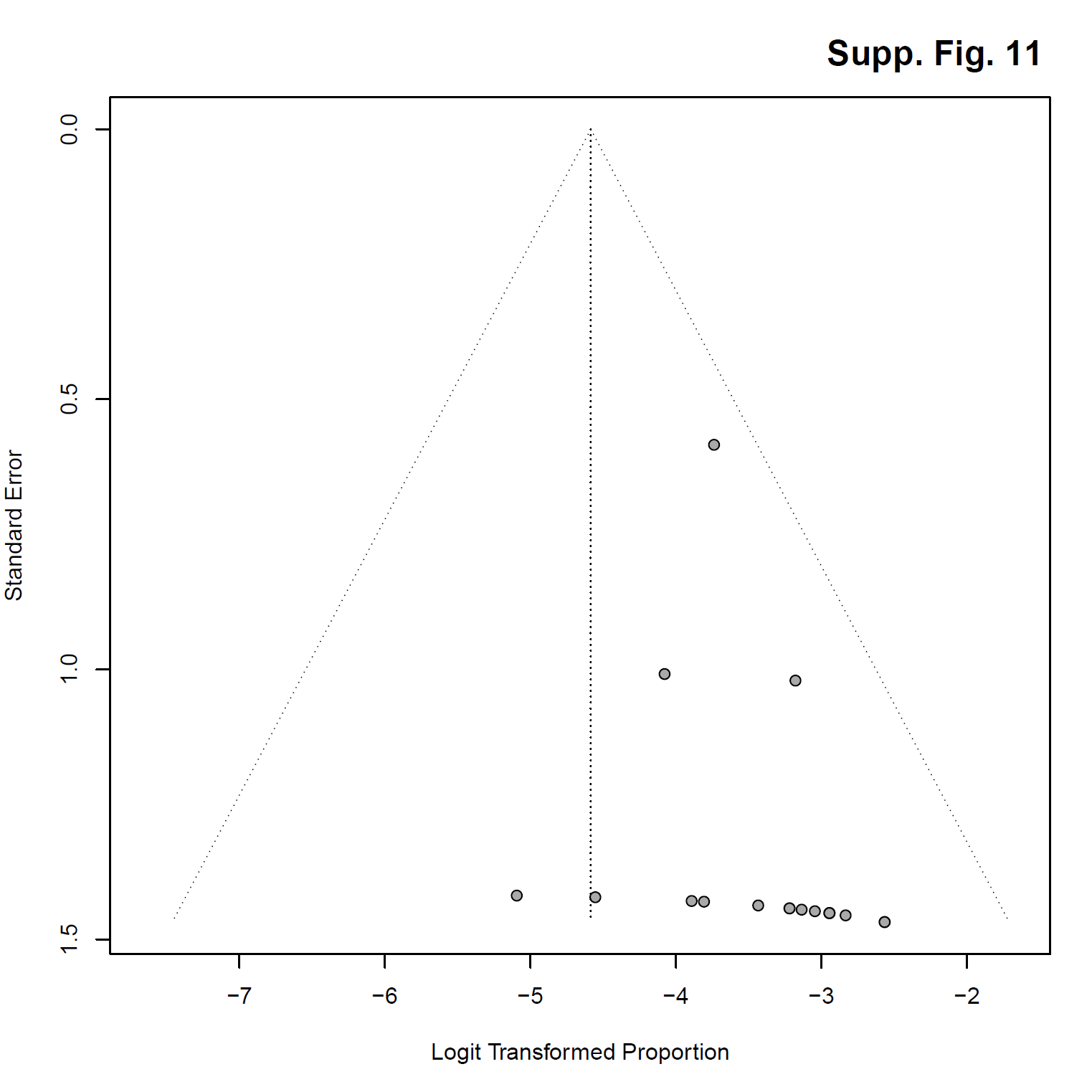


Supplementary Figure 11 Funnel plot of the meta-analysis on cause-specific mortality from hemorrhage in curatively treated patients. The funnel plot’s visual asymmetry stems from logit-scale distortion inherent in studies with zero events, representing a false-positive visual impression. (Egger’s test: t = 1.29, p-value = 0.214; Peter’s test: t = 1.06, p-value = 0.483).


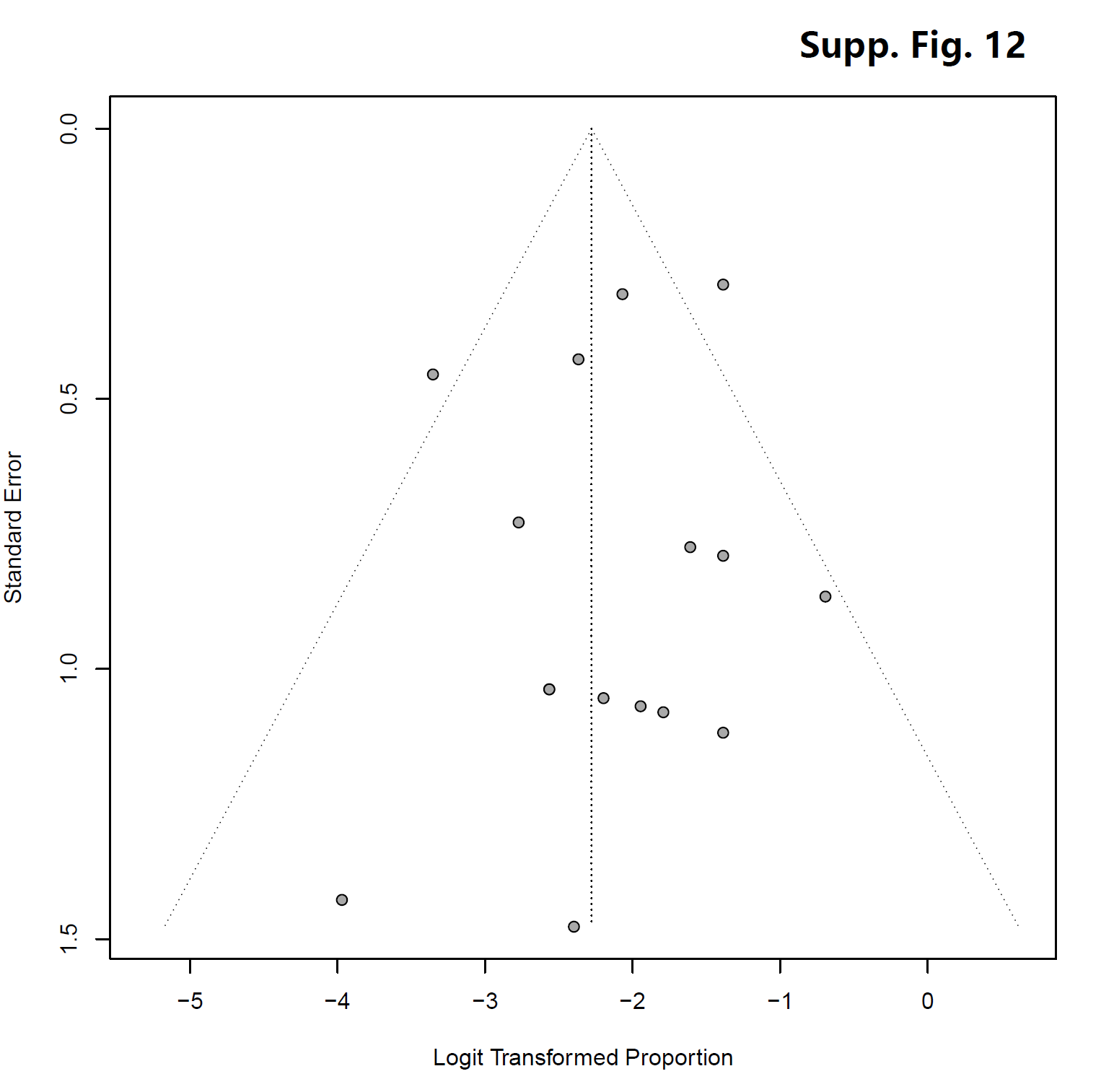


Supplementary Figure 12 Funnel plot of the meta-analysis on cause-specific mortality from hemorrhage in non-curatively treated patients.


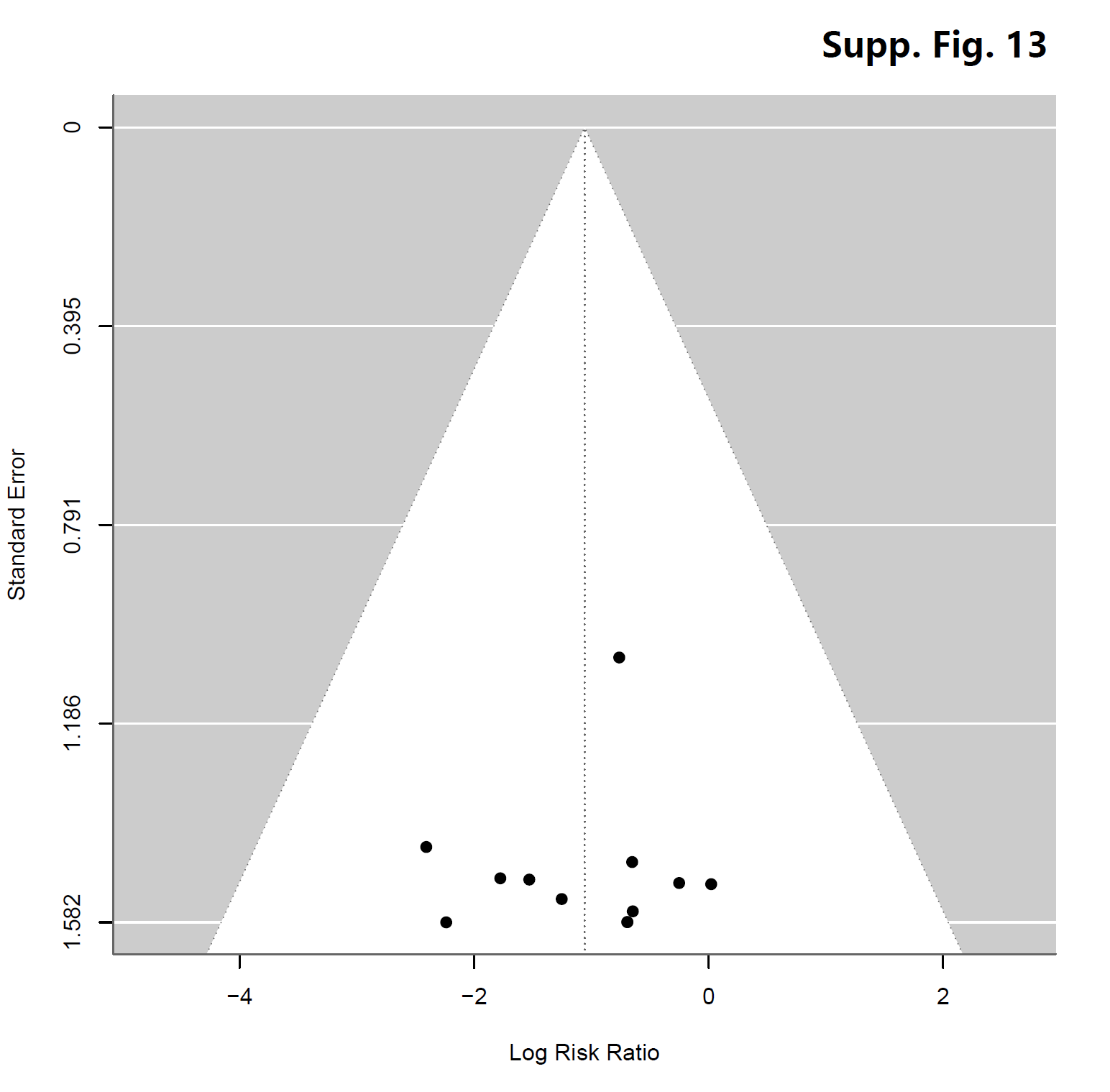


Supplementary Figure 13 Funnel plot of the meta-analysis on the pooled relative risk (RR) for cause-specific mortality between the curative and non-curative groups.


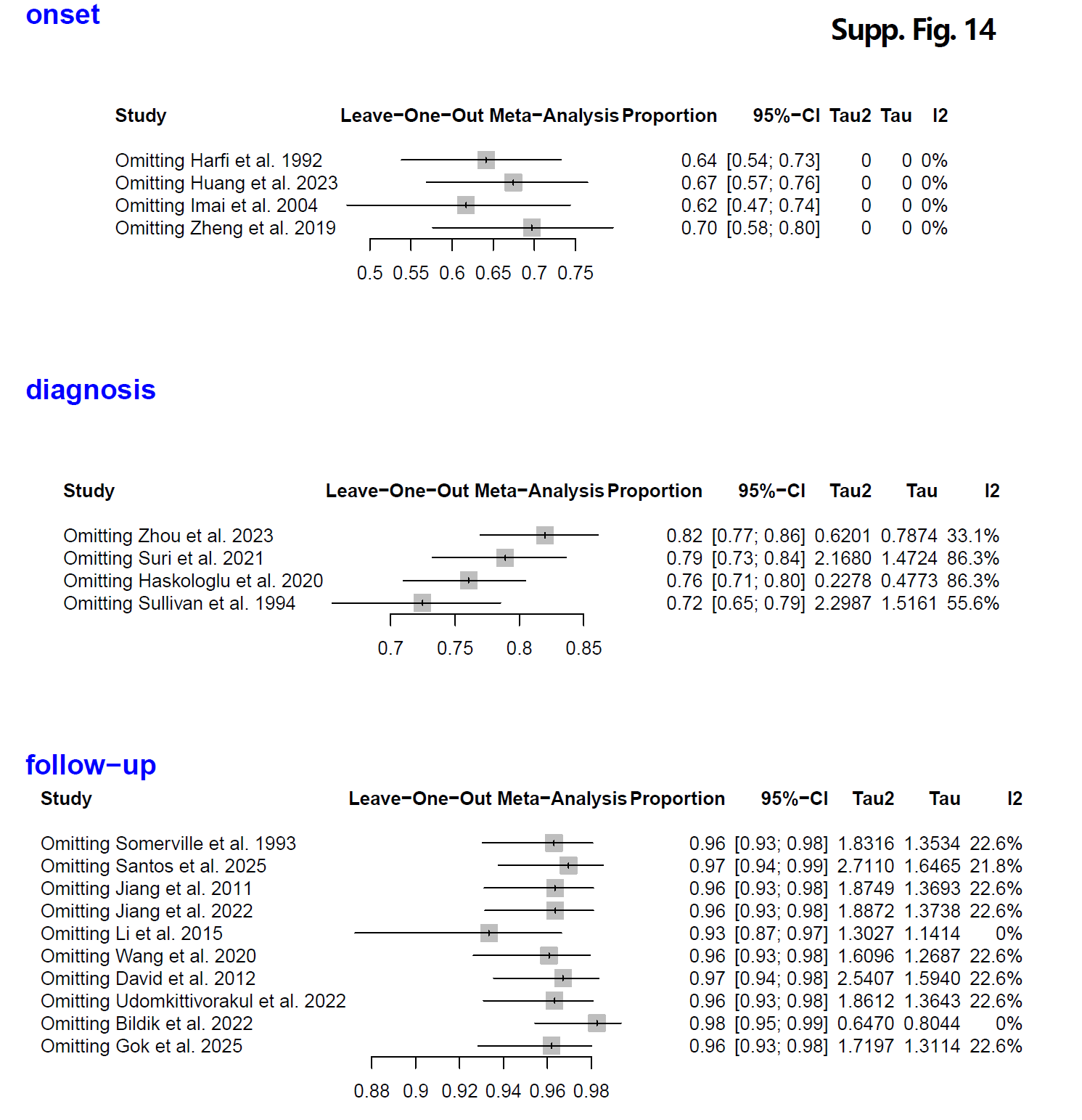


Supplementary Figure 14 Forest plot of leave-one-out sensitivity analysis for the pooled cumulative incidence of overall bleeding.


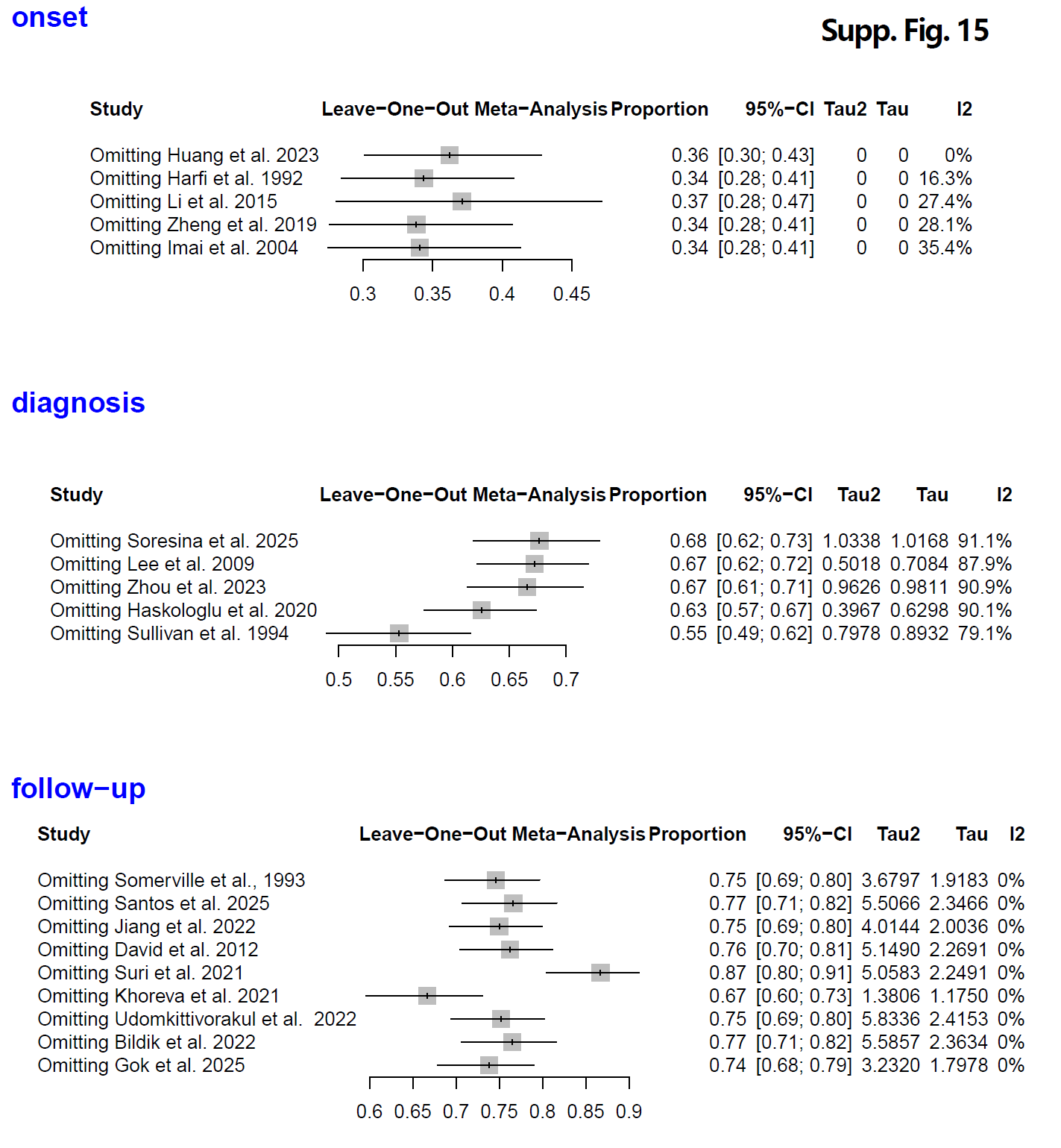


Supplementary Figure 15 Forest plot of leave-one-out sensitivity analysis for the pooled cumulative incidence of cutaneous bleeding.


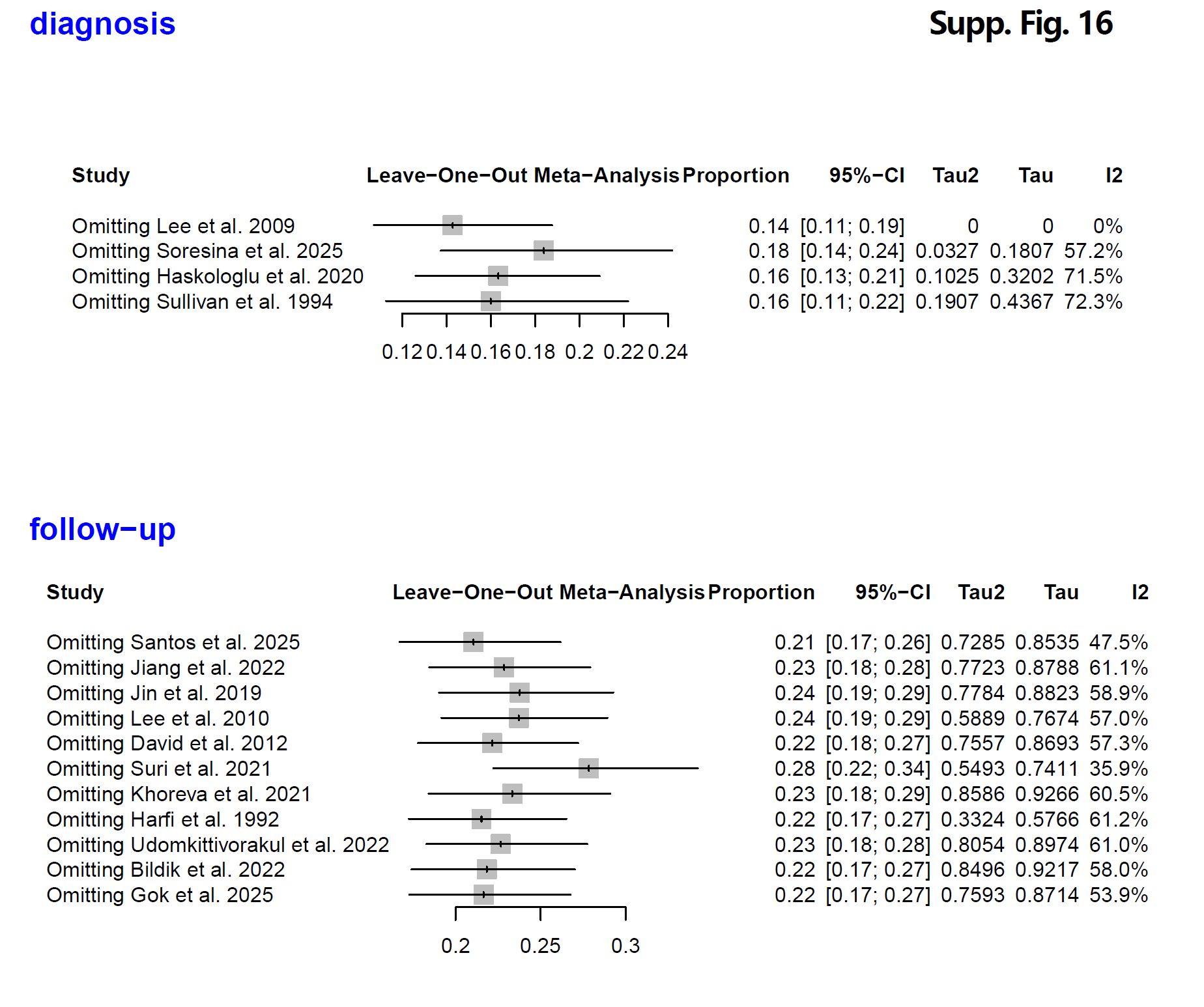


Supplementary Figure 16 Forest plot of leave-one-out sensitivity analysis for the pooled cumulative incidence of epistaxis.


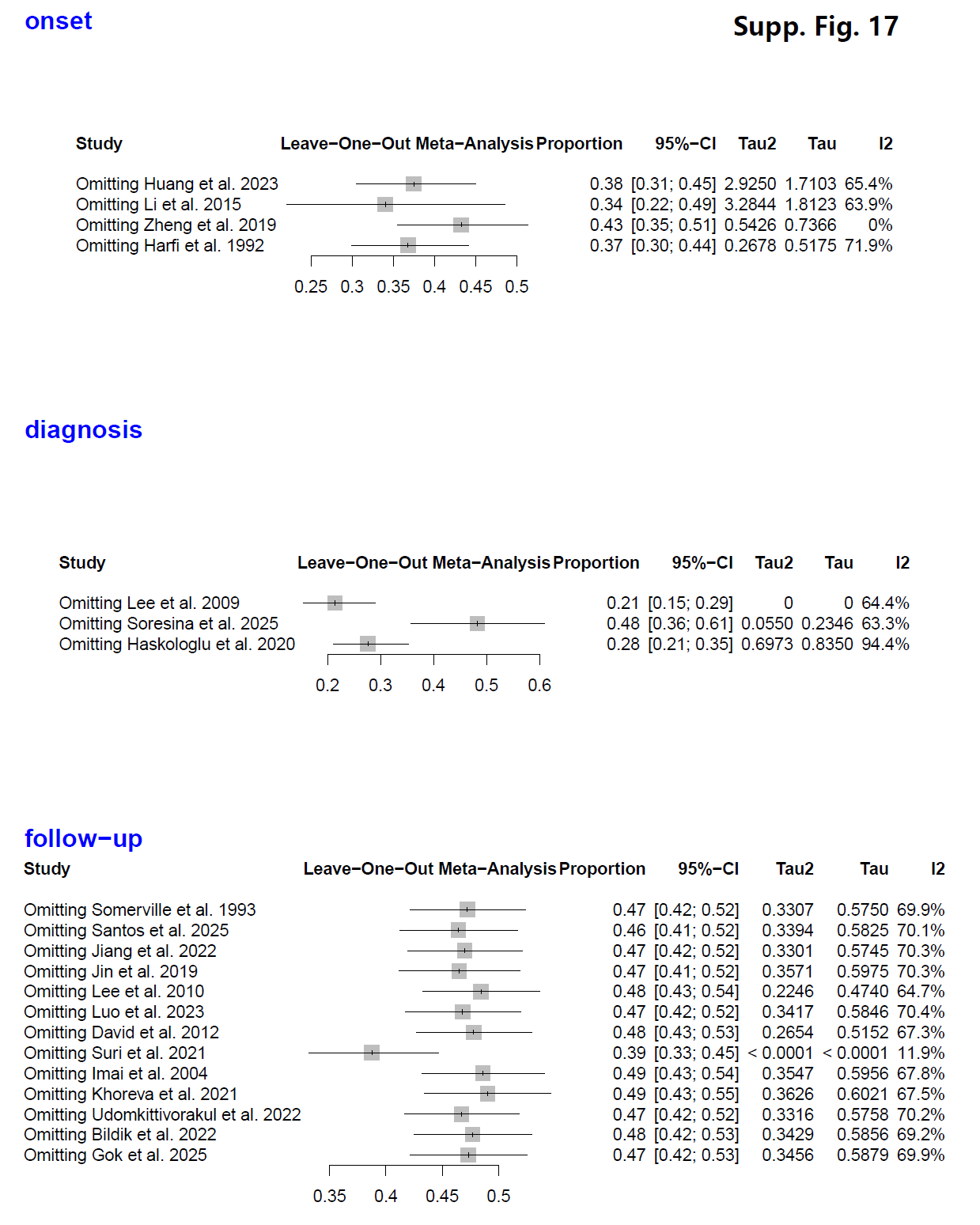


Supplementary Figure 17 Forest plot of leave-one-out sensitivity analysis for the pooled cumulative incidence of gastrointestinal bleeding.


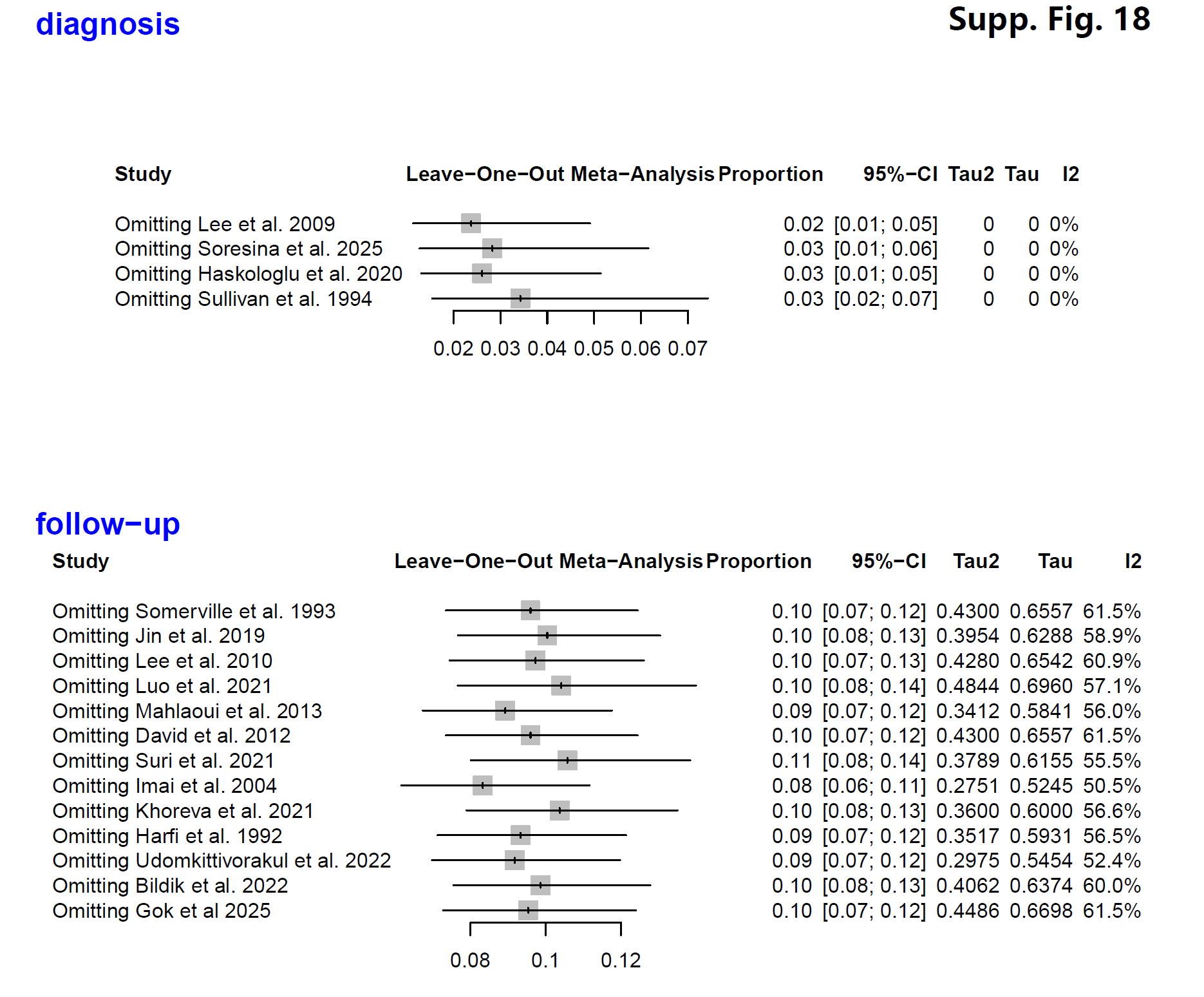


Supplementary Figure 18 Forest plot of leave-one-out sensitivity analysis for the pooled cumulative incidence of intracranial hemorrhage.


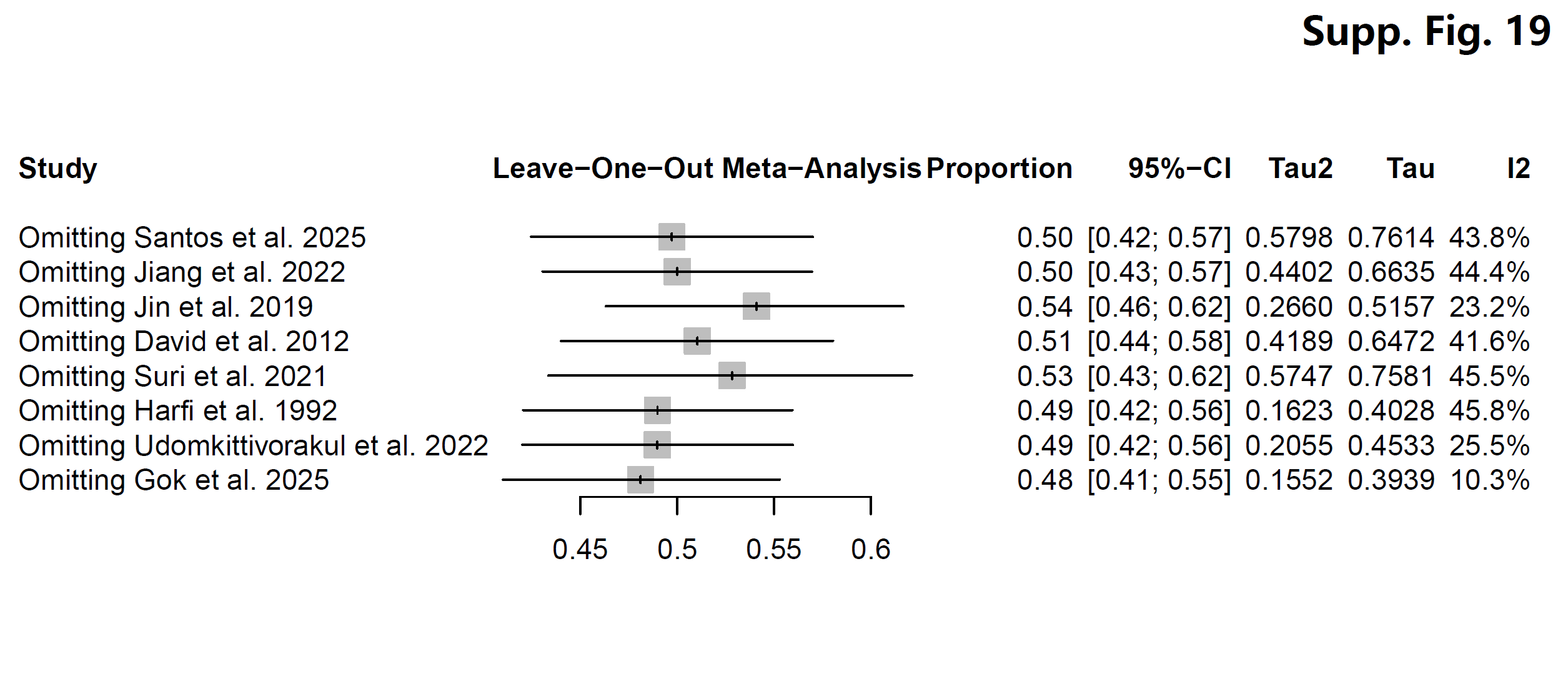


Supplementary Figure 19 Forest plot of leave-one-out sensitivity analysis for the pooled cumulative incidence of multisystem bleeding at the end of follow-up.


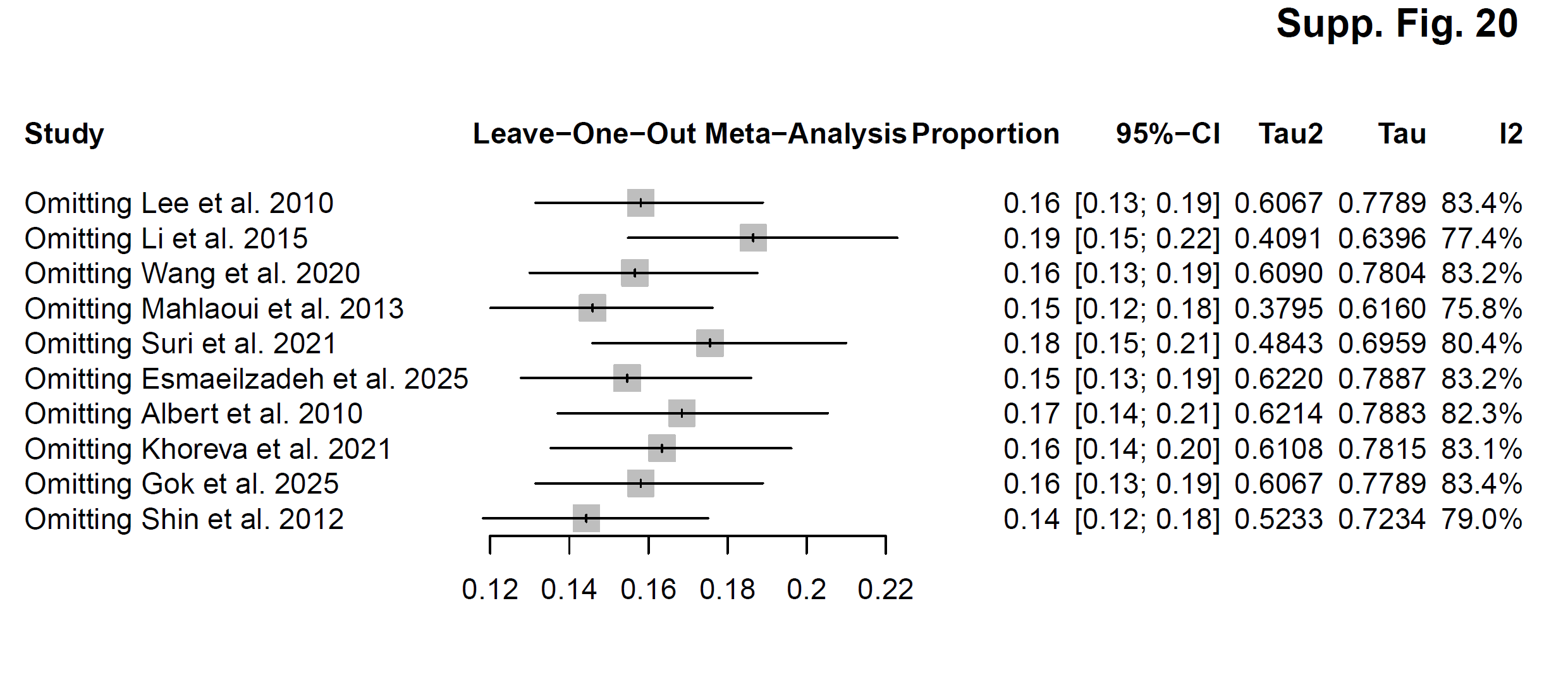


Supplementary Figure 20 Forest plot of leave-one-out sensitivity analysis for the pooled cumulative incidence of severe bleeding at the end of follow-up.
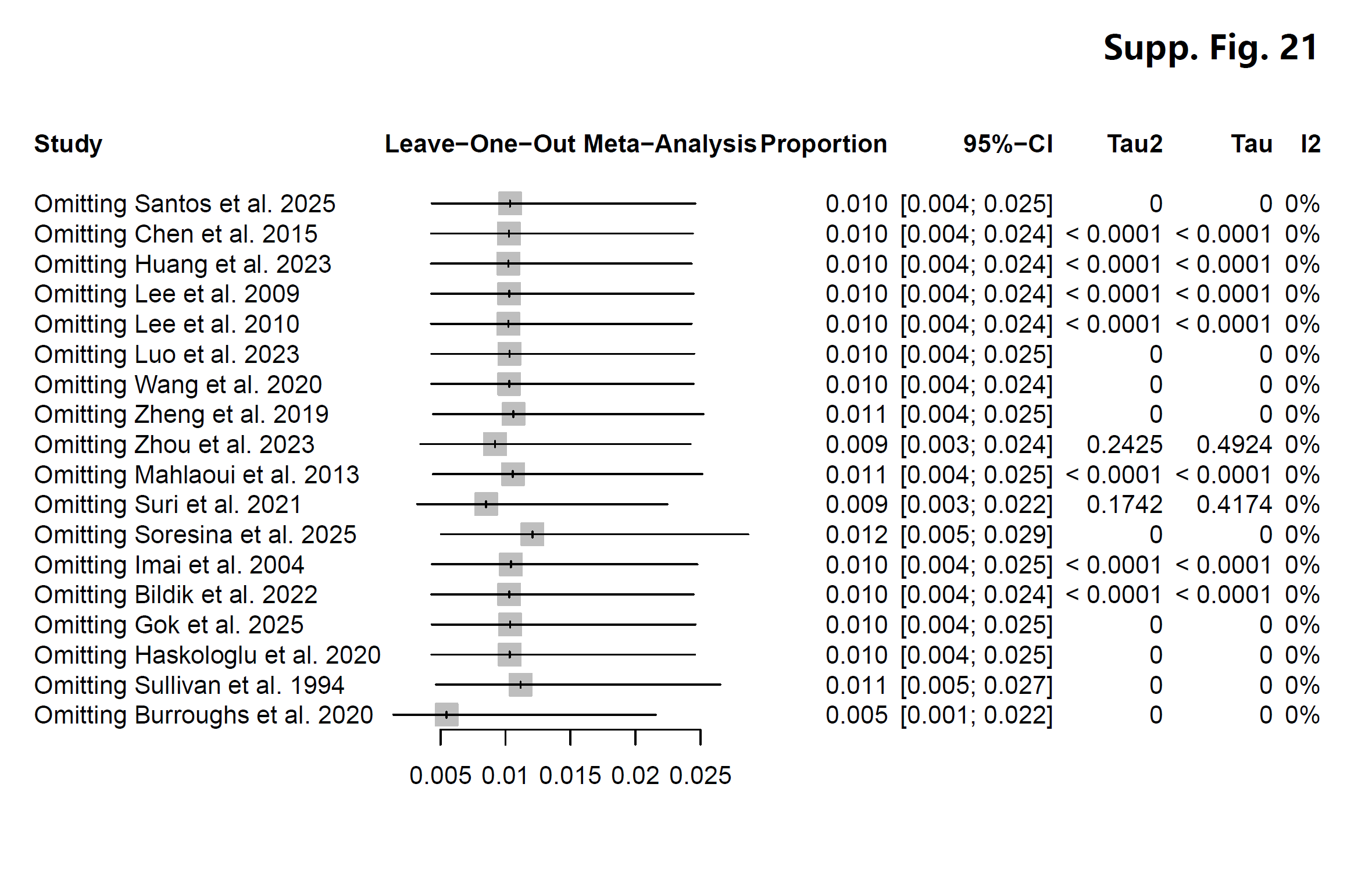


Supplementary Figure 21 Forest plot of leave-one-out sensitivity analysis for cause-specific mortality from hemorrhage in curatively treated patients.


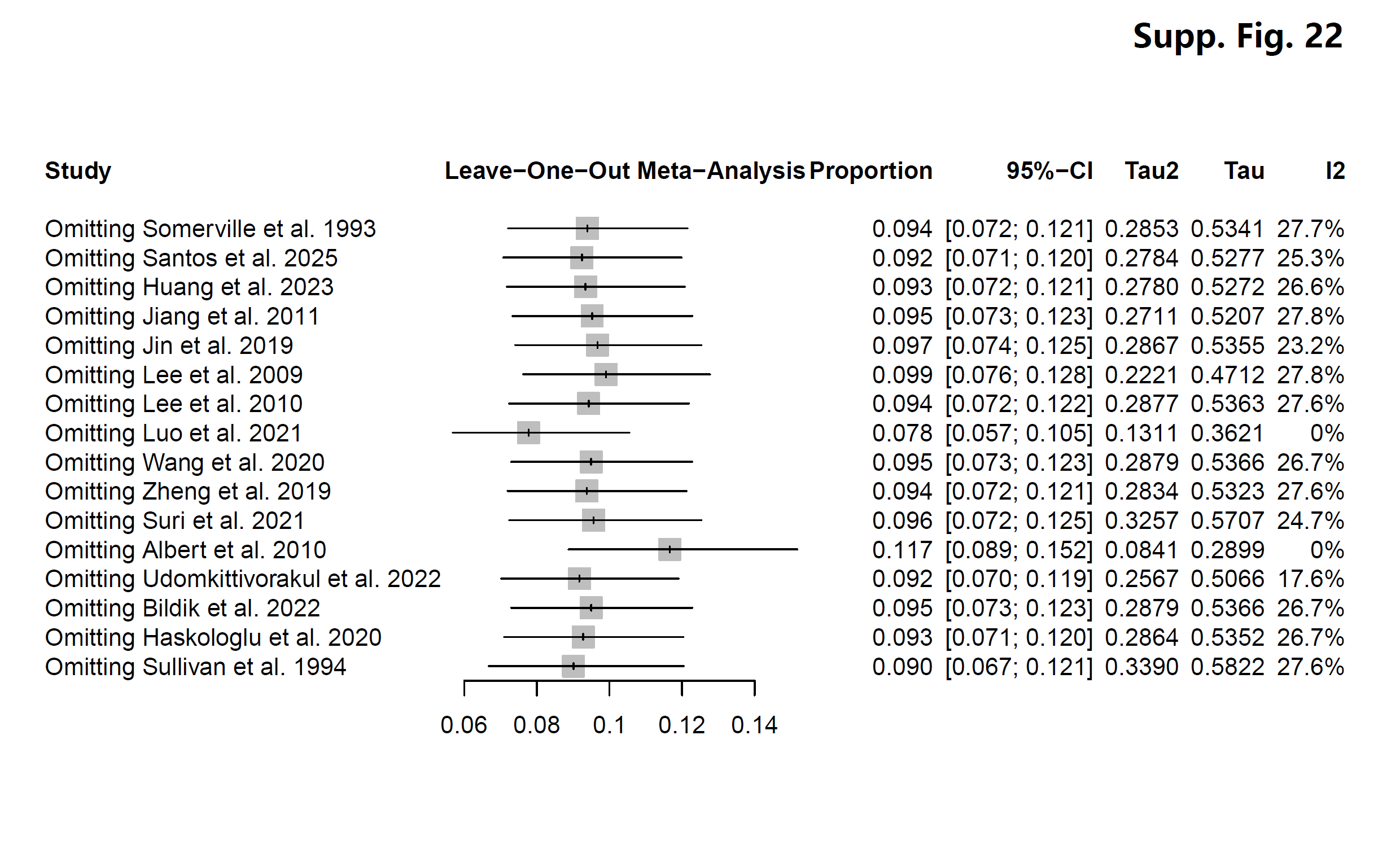


Supplementary Figure 22 Forest plot of leave-one-out sensitivity analysis for cause-specific mortality from hemorrhage in non-curatively treated patients.


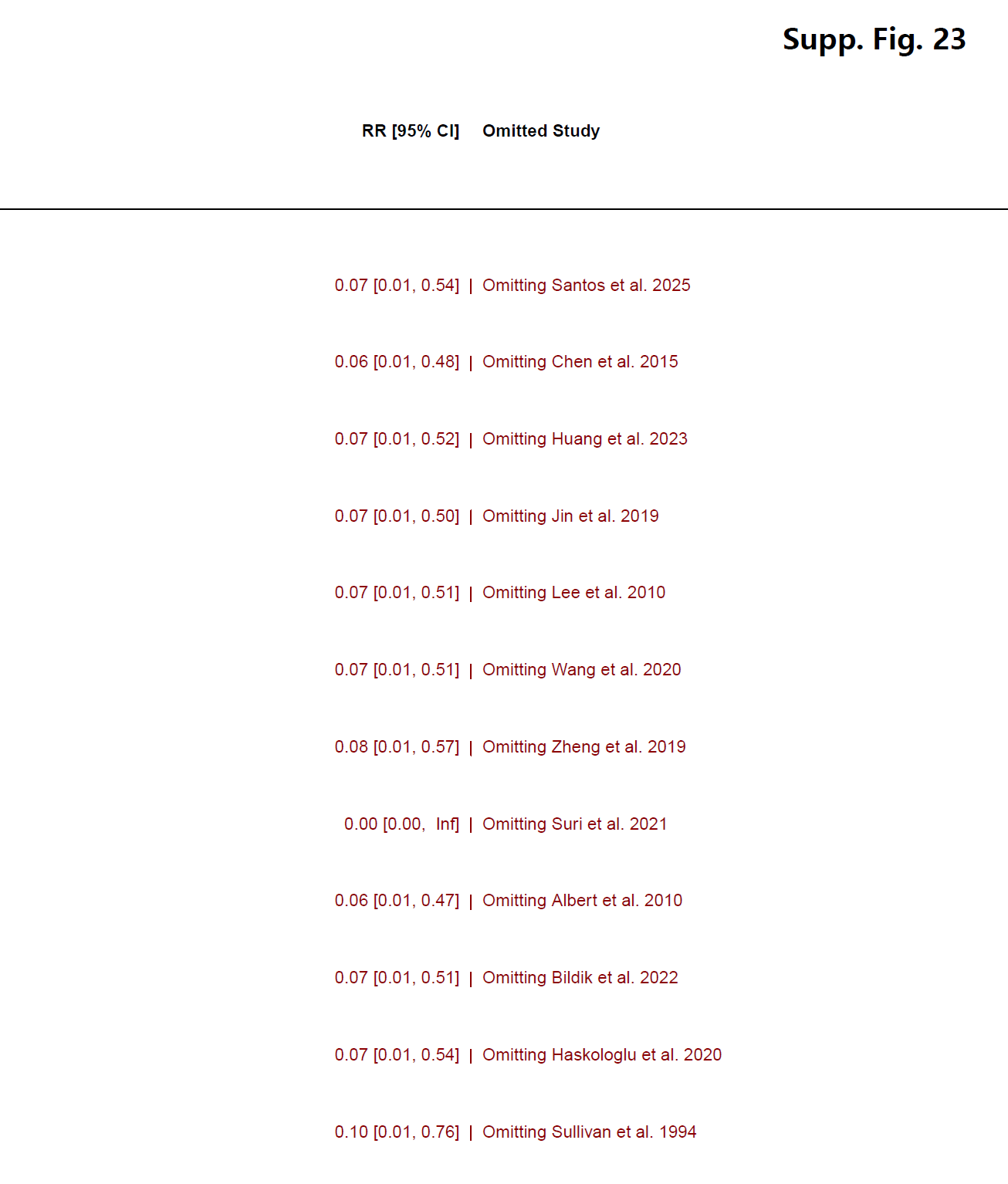


Supplementary Figure 23 Leave-one-out sensitivity analysis for the pooled relative risk (RR) of cause-specific mortality between the curative and non-curative groups.


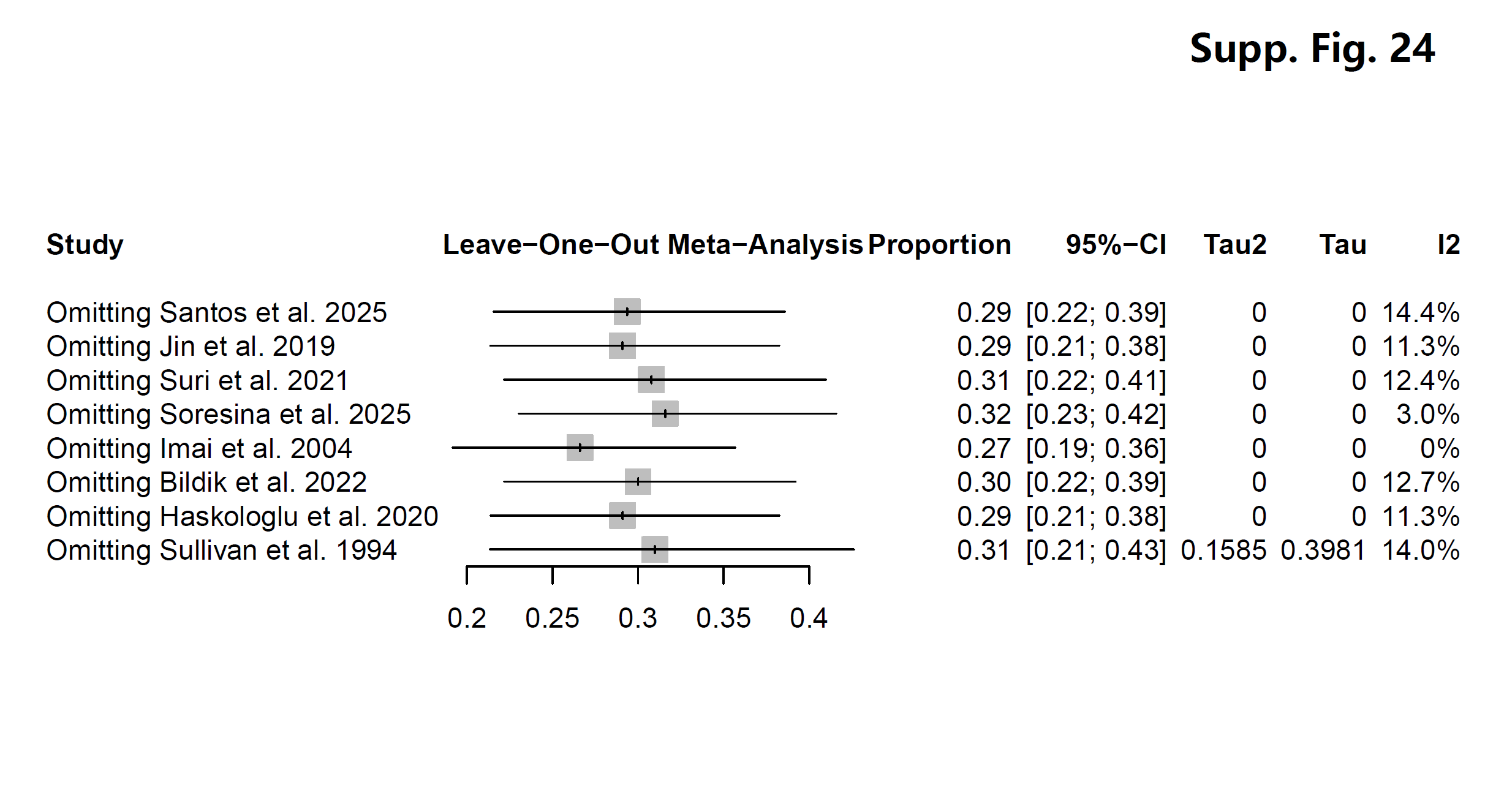


Supplementary Figure 24 Forest plot of leave-one-out sensitivity analysis for the pooled proportional mortality ratio due to hemorrhage in non-curatively treated patients.

# References

1. Somerville C, Forsyth KD. Wiskott aldrich syndrome: An immunodeficiency syndrome not rare in western australia. *Pediatr Allergy Immunol : Off Publ Eur Soc Pediatr Allergy Immunol* (1993) 4:65–72. doi: 10.1111/j.1399-3038.1993.tb00069.x

2. Santos LW, Medina SS, Frade-Guanaes JO, Siqueira LH, de Lima LGR, Chati B, Nolasco da Silva MT, Riccetto AGL, Lyra P, Falcão ACAM, et al. New insights into Wiskott-Aldrich syndrome: Ten novel WAS mutations and their clinical impact in a Brazilian cohort. *Front Immunol* (2025) 16:1585594. doi: 10.3389/fimmu.2025.1585594

3. Chen N, Zhang Z-Y, Liu D-W, Liu W, Tang X-M, Zhao X-D. The clinical features of autoimmunity in 53 patients with Wiskott-Aldrich syndrome in China: a single-center study. *Eur J Pediatr* (2015) 174:1311–1318. doi: 10.1007/s00431-015-2527-3

4. Huang W, Chen L, Wei J, Yang Y, Zhao Y. Clinical and genetic characteristics of 11 neonates with Wiskott-Aldrich syndrome. *Chin J Neonatol* (2023) 38:215–219. doi: 10.3760/cma.j.issn.2096-2932.2023.04.005

5. Jiang M, Wang Z, Su J, Cao L, Li J, Sun X, Bai X, Wang G, Ruan C. Analysis of clinical features and gene mutations in 6 patients with Wiskott-Aldrich syndrome. *Chin J Hemato* (2011) 32:577–582.

6. Jiang J, Zhou J, Wei M, Singh S, Nikuze L, Huang L, Li Y, Jiang J, Wei H. Clinical and molecular characteristics of Wiskott-Aldrich syndrome in five unrelated chinese families. *Scand J Immunol* (2022) 95:e13115. doi: 10.1111/sji.13115

7. Jin Y-Y, Wu J, Chen T-X, Chen J. When WAS gene diagnosis is needed: seeking clues through comparison between patients with Wiskott-Aldrich syndrome and idiopathic thrombocytopenic purpura. *Front Immunol* (2019) 10:1549. doi: 10.3389/fimmu.2019.01549

8. Lee W-I, Yang C-Y, Jaing T-H, Huang J-L, Chien Y-H, Chang K-W. Clinical aspects and molecular analysis of Chinese patients with Wiskott-Aldrich syndrome in taiwan. *Int Arch Allergy Immunol* (2008) 145:15–23. doi: 10.1159/000107462

9. Lee PPW, Chen T-X, Jiang L-P, Chen J, Chan K-W, Lee T-L, Ho MHK, Nong S-H, Yang Y, Fang Y-J, et al. Clinical and molecular characteristics of 35 Chinese children with Wiskott-Aldrich syndrome. *J Clin Immunol* (2009) 29:490–500. doi: 10.1007/s10875-009-9285-9

10. Lee W-I, Huang J-L, Jaing T-H, Wu K-H, Chien Y-H, Chang K-W. Clinical aspects and genetic analysis of taiwanese patients with Wiskott-Aldrich syndrome protein mutation: the first identification of X-linked thrombocytopenia in the Chinese with novel mutations. *J Clin Immunol* (2010) 30:593–601. doi: 10.1007/s10875-010-9381-x

11. Li W, Liu D, Zhang X, Ding Y, Zhao X. Clinical features and genotype analysis of 132 patients with Wiskott-Aldrich syndrome. *Chin J Pediatr* (2015) 53:925–930.

12. Luo XZ, Du X, Li WY, Zhao Q, Liu DW, Zhou LN, Wu JF, Tang XM, Zhao XD, Du HQ. Clinical characteristics and risk factors of deaths in patients with Wiskott-Aldrich syndrome. *Chin J Pediatr* (2021) 59:576–581. doi: 10.3760/cma.j.cn112140-20201224-01128

13. Luo J, Peng J, Zhao P-Q, Fei P. CMV retinitis in Wiskott-Aldrich syndrome. *Ocul Immunol Inflamm* (2023) 31:134–141. doi: 10.1080/09273948.2021.1995762

14. Wang R, Zhang R, Zhu G, Yan Y, Ma J, Wu R. Application of clinical parameters and genetic mutation for guiding the individualized diagnosis and treatment of children patients with Wiskott-Aldrich syndrome: analysis of 23 cases in a single-center. *J Clin Hematol (China)* (2020) 33:176–181. doi: 10.13201/j.issn.1004-2806.2020.03.006

15. Zheng Y, Lu Q, Yao Y, He H, Li J, Xiao P, Hu S. Analysis of Correlation of WAS Gene Mutations with Clinical Phenotype. *Journal of experimental hematology (China)* (2019) 27:246–252.

16. Zhou C, Luo CY, Wang JM, Luo CJ, Qin X, Huang XH, Chen J. Therapeutic efficacy of hematopoietic stem cell transplantation for Wiskott-Aldrich syndrome in 60 children. *Chin J Pediatr* (2023) 61:351–356. doi: 10.3760/cma.j.cn112140-20220810-00720

17. Sun Y, Song X, Pan H, Li X, Sun L, Song L, Ma F, Hao J. Wiskott-Aldrich syndrome: A new synonym mutation in the WAS gene. *Intractable Rare Dis Res* (2024) 13:69–72. doi: 10.5582/irdr.2023.01102

18. Mahlaoui N, Pellier I, Mignot C, Jais J-P, Bilhou-Nabéra C, Moshous D, Neven B, Picard C, de Saint-Basile G, Cavazzana-Calvo M, et al. Characteristics and outcome of early-onset, severe forms of wiskott-aldrich syndrome. *Blood* (2013) 121:1511–1516. doi: 10.1182/blood-2012-08-448118

19. Suri D, Rikhi R, Jindal AK, Rawat A, Sudhakar M, Vignesh P, Gupta A, Kaur A, Sharma J, Ahluwalia J, et al. Wiskott-Aldrich syndrome: a multi-institutional experience from India. *Front Immunol* (2021) 12:627651. doi: 10.3389/fimmu.2021.627651

20. David S, Jayandharan GR, Abraham A, Jacob RR, Devi GS, Patkar N, Shaji RV, Nair SC, Viswabandya A, Ahmed R, et al. Molecular basis of Wiskott-Aldrich syndrome in patients from India. *Eur J Haematol* (2012) 89:356–360. doi: 10.1111/j.1600-0609.2012.01818.x

21. Esmaeilzadeh H, Gholami MA, Dehghani SS, Nabavizadeh H, Alyasin S, Rezaei N, Delavari S, Abolhassani H, DorriMoghaddam F, Ghasemi F. Comprehensive clinical and immunologic characterization of Wiskott-Aldrich syndrome in Iran: a 10-year cohort study. *BMC Immunol* (2025) 26:97–111. doi: 10.1186/s12865-025-00779-4

22. Palevski D, Simon A, Lev A, Somech R, Lee YN. Inadequate activation of γδT- and B-cells in patient with Wiskott-Aldrich syndrome (WAS) portrayed by TRG and IGH repertoire analyses. *J Clin Immunol* (2023) 43:109–122. doi: 10.1007/s10875-022-01349-8

23. Soresina A, Rondelli R, Notarangelo LD, Locatelli F, Aiuti A, Biffi A, Rabusin M, Pignata C, Menna G, Prete A, et al. Long-term outcome in Wiskott-Aldrich syndrome and X-linked thrombocytopenia patients: An observational -prospective multi-center study of the italian primary immune deficiency network (IPINET). *EClinicalMedicine* (2025) 84:103271. doi: 10.1016/j.eclinm.2025.103271

24. Imai K, Morio T, Zhu Y, Jin Y, Itoh S, Kajiwara M, Yata J-I, Mizutani S, Ochs HD, Nonoyama S. Clinical course of patients with WASP gene mutations. *Blood* (2004) 103:456–464. doi: 10.1182/blood-2003-05-1480

25. Albert MH, Bittner TC, Nonoyama S, Notarangelo LD, Burns S, Imai K, Espanol T, Fasth A, Pellier I, Strauss G, et al. X-linked thrombocytopenia (XLT) due to WAS mutations: clinical characteristics, long-term outcome, and treatment options. *Blood* (2010) 115:3231–3238. doi: 10.1182/blood-2009-09-239087

26. Khoreva A, Abramova I, Deripapa E, Rodina Y, Roppelt A, Pershin D, Larin S, Voronin K, Maschan A, Novichkova G, et al. Efficacy of romiplostim in treatment of thrombocytopenia in children with Wiskott-Aldrich syndrome. *Br J Haematol* (2021) 192:366–374. doi: 10.1111/bjh.17174

27. Harfi HA, Al-Malik S, Tulba A. Wiskott-Aldrich syndrome. *Ann Saudi Med* (1992) 12:355–361. doi: 10.5144/0256-4947.1992.355

28. Lee EK, Eem Y-J, Chung N-G, Kim MS, Jeong DC. A case of familial X-linked thrombocytopenia with a novel WAS gene mutation. *Korean J Pediatr* (2013) 56:265–268. doi: 10.3345/kjp.2013.56.6.265

29. Udomkittivorakul N, Wattanasirichaigoon D, Manuyakorn W, Pongphitcha P, Khongkraparn A, Tunlayadechanont P, Sirachainan N. Report of clinical presentations and two novel mutations in patients with Wiskott-Aldrich syndrome/X-linked thrombocytopenia. *Platelets* (2022) 33:792–796. doi: 10.1080/09537104.2021.1988549

30. Radl J, Dooren LH, Morell A, Skvaril F, Vossen JM, Uittenbogaart CH. Immunoglobulins and transient paraproteins in sera of patients with the Wiskott-Aldrich syndrome: a follow-up study. *Clin Exp Immunol* (1976) 25:256–263.

31. Gök V, Ozcan A, Mutlu FT, Yılmaz E, Kocak Göl D, Ozay M, Demir B, Taskiran H, Bas H, Mutlu MB, et al. Clinical and laboratory aspects of patients diagnosed with various inherited platelet disorders. *Res Pract Thromb Haemost* (2025) 9:102873. doi: 10.1016/j.rpth.2025.102873

32. Bildik HN, Cagdas D, Ozturk Kura A, Oskay Halacli S, Sanal O, Tezcan I. Clinical, laboratory features and clinical courses of patients with Wiskott-Aldrich syndrome and X-linked thrombocytopenia-a single center study. *Immunol Invest* (2022) 51:1272–1283. doi: 10.1080/08820139.2021.1933516

33. Haskoloğlu Ş, Öztürk A, Öztürk G, Kostel Bal S, İslamoğlu C, Baskın K, Ceylaner S, Tufan Satıroğlu L, Doğu F, İkincioğulları A. Clinical features and outcomes of 23 patients with Wiskott-Aldrich syndrome: a single-center experience. *Turk J Haematol* (2020) 37:271–281. doi: 10.4274/tjh.galenos.2020.2020.0334

34. Faganello G, Hamilton M, Wilde P, Turner MS. Percutaneous closure of false aneurysms of the aorta in Wiskott-Aldrich syndrome. *Eur Heart J* (2008) 29:6. doi: 10.1093/eurheartj/ehm349

35. Sullivan KE, Mullen CA, Blaese RM, Winkelstein JA. A multiinstitutional survey of the Wiskott-Aldrich syndrome. *J Pediatr* (1994) 125:876–885. doi: 10.1016/s0022-3476(05)82002-5

36. Mathew P, Conley ME. Effect of intravenous gammaglobulin (IVIG) on the platelet count in patients with Wiskott-Aldrich syndrome. *Pediatr Allergy Immunol* (1995) 6:91–94. doi: 10.1111/j.1399-3038.1995.tb00265.x

37. Lum LG, Tubergen DG, Corash L, Blaese RM. Splenectomy in the management of the thrombocytopenia of the Wiskott-Aldrich syndrome. *N Engl J Med* (1980) 302:892–896. doi: 10.1056/NEJM198004173021604

38. Shin CR, Kim M-O, Li D, Bleesing JJ, Harris R, Mehta P, Jodele S, Jordan MB, Marsh RA, Davies SM, et al. Outcomes following hematopoietic cell transplantation for Wiskott-Aldrich syndrome. *Bone Marrow Transplant* (2012) 47:1428–1435. doi: 10.1038/bmt.2012.31

39. Perry GS 3rd, Spector BD, Schuman LM, Mandel JS, Anderson VE, McHugh RB, Hanson MR, Fahlstrom SM, Krivit W, Kersey JH. The Wiskott-Aldrich syndrome in the United States and Canada (1892-1979). *J Pediatr* (1980) 97:72–78. doi: 10.1016/s0022-3476(80)80133-8

40. Burroughs LM, Petrovic A, Brazauskas R, Liu X, Griffith LM, Ochs HD, Bleesing JJ, Edwards S, Dvorak CC, Chaudhury S, et al. Excellent outcomes following hematopoietic cell transplantation for Wiskott-Aldrich syndrome: a PIDTC report. *Blood* (2020) 135:2094–2105. doi: 10.1182/blood.2019002939
